# LiO*^t^*Bu-Promoted Intramolecular 1,3-Dipolar Cycloaddition of the 2′-Alkynyl-biaryl-2-aldehyde *N*-Tosylhydrazones Approach to 3-Substituted 1*H*-Dibenzo[*e*,*g*]indazoles

**DOI:** 10.3390/molecules28248061

**Published:** 2023-12-13

**Authors:** Jiaying Lv, Ruimao Hua

**Affiliations:** 1 Key Laboratory of Organic Optoelectronics & Molecular Engineering of Ministry of Education, Department of Chemistry, Tsinghua University, Beijing 100084, China; jy-lv18@mails.tsinghua.edu.cn; 2 State Key Laboratory of Chemistry and Utilization of Carbon-Based Energy Resources, College of Chemistry, Xinjiang University, Urumqi 830017, China

**Keywords:** *N*-tosylhydrazones, 1*H*-dibenzo[*e*,*g*]indazoles, intramolecular cycloaddition, lithium *tert*-butoxide, hydrogen bonds

## Abstract

A two-step, one-pot synthesis of 3-substituted 1*H*-dibenzo[*e*,*g*]indazoles in good to high yields via a LiO*^t^*Bu-promoted intramolecular 1,3-dipolar cyclization of 2′-alkynyl-biaryl-2-aldehyde *N*-tosylhydrazones was developed. The *N*-Ts-hydrazones used were prepared in situ via the reactions of 2′-alkynyl-biaryl-2-aldehydes and TsNHNH_2_ *(p*-methylbenzenesulfonohydrazide). Two types of signals related to the hydrogen bonds, forming in several products, were observed in the ^1^H NMR spectra recorded in DMSO-*d*_6_, assigned to N-H bonds in their dimeric species of product and tautomer.

## 1. Introduction

1,3-dipolar cycloadditions of azides to alkynes, as the most important representative reactions in click chemistry and bio-orthogonal chemistry, have attracted enormous attention in the past decades [1,2,3,4,5]. Besides azides, diazo compounds’ 1,3-dipoles could also be used in 1,3-dipolar cycloadditions to react with alkynes, providing diverse pyrazole-based skeletons [6,7]. Recently, numerous elegant works, involving cycloadditions between diazo compounds (or their *N*-tosylhydrazone precursors) and alkynes, were reported [8,9,10,11,12,13,14,15,16,17,18,19,20]. However, the design of *N*-tosylhydrazones for intramolecular 1,3-dipolar cycloadditions to construct π-extended pyrazole-based skeletons is rarely reported.

Nowadays, indazole derivatives comprising a pyrazole ring represent one of the most important heterocyclic scaffolds in the pharmaceutical industry [21,22,23], possessing a variety of biological activities, such as antimicrobial [24], anti-inflammatory [25], and anti-HIV [26] properties. 1*H*-indazoles, as one of the tautomeric forms of indazole, have more thermodynamic stability than 2*H*-indazoles [27].

Since the synthesis of 2*H*-dibenzo[*e*,*g*]indazoles has already been reported [28], in the present work, we opted for a synthetic method towards 1*H*-dibenzo[*e*,*g*]indazoles, providing more possibilities of indazole-based derivatives in a further exploration of pharmaceutical molecules or larger polycyclic aromatic compounds (PACs).

**Scheme 1:**
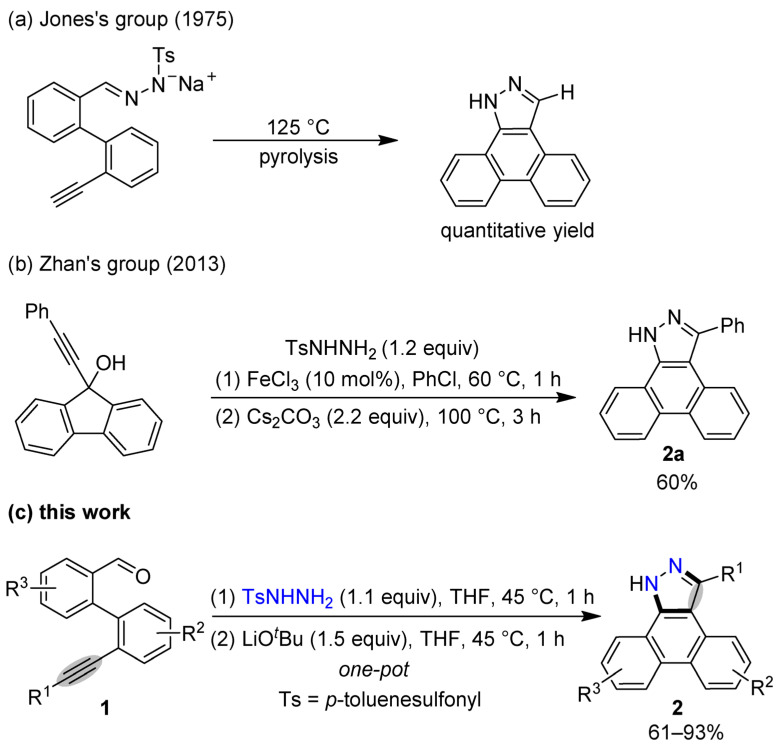
The syntheses of some 1*H*-dibenzo[*e*,*g*]indazoles from different starting materials [29,30].

In 1975, Jones’s group reported a pyrolytic method towards 1*H*-dibenzo[*e*,*g*]indazole from 2′-ethynyl-biaryl-2-aldehyde *N*-tosylhydrazone sodium-salt in a quantitative yield [29] (Scheme 1a). In 2013, Zhan’s group synthesized 3-phenyl-substituted 1*H*-dibenzo[*e*,*g*]indazole (**2a**) in a 60% yield from a ring-expansion strategy of 9-(phenylethynyl)-9*H*-fluoren-9-ol [30] (Scheme 1b). We note that only one example was given in each of the cited references, and either high temperatures or complicated starting materials were required.

Based on our previous studies on the use of *N*-tosylhydrazones in cyclizations [31,32,33], herein we report a one-pot synthetic method towards 3-substituted 1*H*-dibenzo[*e*,*g*]indazoles (**2**) from 2′-alkynyl-biaryl-2-aldehyde *N*-tosylhydrazones, which was then optimized to a one-pot two-steps manner starting from 2′-alkynyl-biaryl-2-aldehydes (**1**) (Scheme 1c). In addition, in order to explain the observed two types of N-H signals in the ^1^H NMR spectra in DMSO-*d*_6_, the formation of dimeric species is proposed, which is supported by the X-ray structure of one product with the studies of DFT (density functional theory) calculations using the Gaussian 09 program [34] with an SMD solvation model [35].

## 2. Results and Discussion

### 2.1. Synthesis

#### 2.1.1. Optimization of Model Reaction Conditions

Our investigations started from hydrazone **1a′**, easily available from biarylaldehyde **1a** and TsNHNH_2_ (*p*-methylbenzenesulfonohydrazide) in methanol at room temperature. When the reaction of hydrazone **1a′** (1.0 equiv.) and LiO*^t^*Bu (1.5 equiv.) in tetrahydrofuran (THF) was heated at 100 °C for 2 h, 3-phenyl-1*H*-dibenzo[*e*,*g*]indazole (**2a**) could be isolated in an 89% yield (Table 1, entry 1). When the temperature was progressively diminished at 50 °C, 45 °C, 35 °C, or 25 °C, the yields of **2a** did not significantly decrease except for 25 °C (Table 1, entries 2–5). Repeating the reaction in THF at 45 °C for 1 h, the yield of **2a** could be maintained in an 88% yield (Table 1, entry 6). Since hydrazone **1a′** was prepared in methanol, we then examined the reaction of hydrazone **1a′** in this solvent, rather than in THF, but the yield of **2a** decreased to 68% (Table 1, entry 7).

Therefore, the condensation between biarylaldehyde **1a** and TsNHNH_2_ in THF at 45 °C was further examined to explore the possibility of developing a two-step, one-pot procedure **1a** → **2a**. It was found that **1a** could be totally converted into hydrazone **1a′** after 1 h (TLC monitoring). Moreover, when LiO*^t^*Bu (1.5 equiv.) and 2.5 mL of THF were added to the reaction mixture of entry 6, **2a** could be obtained in an 88% yield after additional heating for 1 h. We also tried a one-step process at 45 °C: by adding TsNHNH_2_ and LiO*^t^*Bu at the same time, 79% of **2a** could be acquired. Thus, the one-pot/two-step process in entry 6 was considered as the optimized condition.

**Scheme 2:**
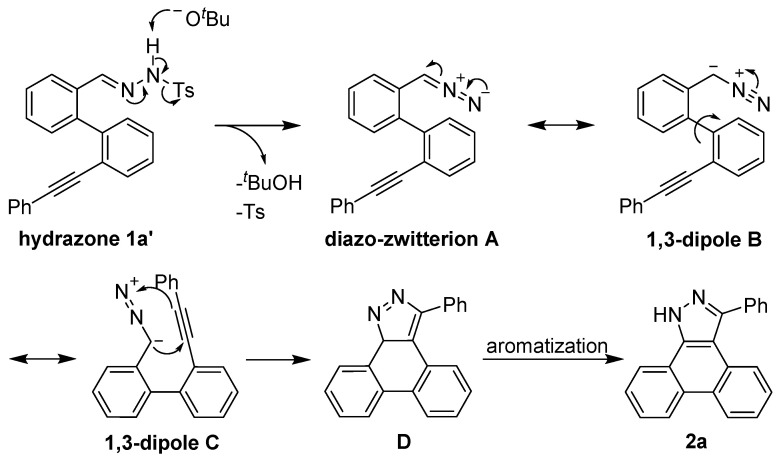
Proposed mechanism of **2a** formation.

We also examined the formation of **2a** from **1a** using other inorganic alkali, such as NaO*^t^*Bu, KO*^t^*Bu, Li_2_CO_3_, Na_2_CO_3_, K_2_CO_3_, and Cs_2_CO_3_. As shown in Table 2, the use of NaO*^t^*Bu and KO*^t^*Bu resulted in the formation of **2a** in 81% and 85% yields, respectively (entries 2 and 3), similar to the yield of LiO*^t^*Bu (entry 1). However, in the presence of Li_2_CO_3_, Na_2_CO_3_, K_2_CO_3_, and Cs_2_CO_3_, **2a** only formed in 9–14% yields (entries 4–7). These results support the proposed mechanism depicted in Scheme 2 (vide infra), in which a *tert*-butoxide anion (*^t^*BuO^−^) made a main contribution to the intramolecular cyclization by promoting the formation of the diazo zwitterion **A**.

#### 2.1.2. Substrates’ Expansion under Optimized Conditions

**Chart 1:**
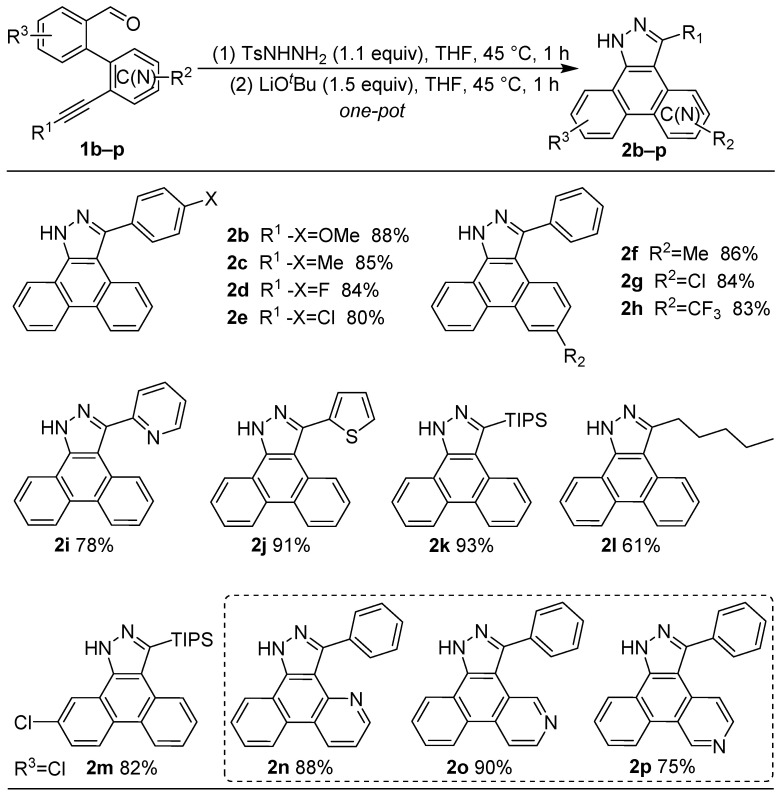
Extension of the optimized methodology as one-pot/two-step synthesis of 3-substituted-1*H*-dibenzo[*e*,*g*]indazoles **2b**–**p** *^a^*. *^a^* Reaction conditions: **1** (1.0 mmol), TsNHNH_2_ (1.1 equiv.) in 5.0 mL of THF at 45 °C for 1 h, then LiO*^t^*Bu (1.5 equiv.) and additional 2.5 mL of THF at 45 °C for 1 h. In each case, the yield refers to the effective amount of isolated compound.

Thus, the extension of the above optimized methodology as a one-pot/two-step synthesis of 3-substituted-1*H*-dibenzo[*e*,*g*]indazoles **2b**–**p** starting from 2′-alkynyl-biaryl-2-aldehydes **1b**–**p** via an intramolecular 1,3-dipolar cycloaddition is depicted in Chart 1. The corresponding *p*-Ts-hydrazones of **1b**–**p**, as crucial transformations, exhibited their quantitative feasibility by being modulated with the influence of alkyne-substituents R^1^ and Ar-substituents R^2^ and R^3^.

Aromatic alkynyl substrates bearing either electron-donating groups (R^1^−X = *p*-methoxy (**1b**), R^1^−X = *p*-methyl (**1c**)) or electron-withdrawing groups (R^1^−X = *p*-fluoro (**1d**), R^1^−X = *p*-chloro (**1e**)) underwent the condensation reactions smoothly to give **2b**–**e** in 80–88% yields. Moreover, pyridyl-(**1i**), thienyl-(**1j**), and silyl-(**1k**) substituted substrates showed a good tolerance, providing **2i**–**k** in 78–93% yields. However, the substrate having an alkyl alkynyl group (**1l**) showed a slightly lower reactivity, giving **2l** in a 61% yield.

In addition, the introduction of methyl (**1f**), chloro (**1g**), and trifluoromethyl (**1h**) groups at the position of R^2^ showed a similar reactivity to **1a** in producing **2f**–**h** in 83–86% yields. In the case of the substrate having chloro and silyl groups (**1m**), the corresponding product **2m** could be also obtained in an 82% yield. More interestingly, three pyridyl-fused analogues of **2a**, **2n**–**p** were also successfully synthesized in 88%, 90%, and 75% yields, respectively.

### 2.2. Proposed Mechanism

The proposed mechanism of 3-phenyl-1*H*-dibenzo[*e*,*g*]indazole (**2a**) formation is depicted in Scheme 2. In the presence of *^t^*BuO^−^, diazo zwitterion **A** forms from hydrazone **1a′**; then, an intramolecular nucleophilic cycloaddition occurs in the **1,3-dipole C** to afford **D**, which, then, allows aromatization to take place to give the final product **2a**.

### 2.3. Structural Analysis

#### 2.3.1. X-ray Data of Compound **2a**

**Figure 1:**
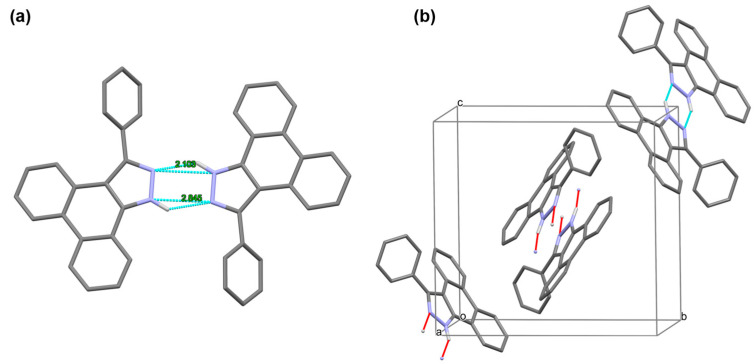
X-ray crystal structure of compound **2a**. Carbon atoms are shown in gray, nitrogen atoms in purple, and N-H hydrogen atoms in white. The hydrogen atoms on the benzene rings are omitted for clarity. (**a**) View face-on to the aromatic rings showing the hydrogen bonds in the **2a** dimer. Annotated with the NH–N hydrogen bonds’ length and N-N distance. (**b**) View of a unit cell. The blue lines show the NH–N hydrogen bonds in the **2a** dimer. The red lines show the NH–N hydrogen bonds between **2a** and another omitted molecule.

A suitable single crystal of compound **2a** was obtained through a slow evaporation from its petroleum ether/dichloromethane (5:1 *v*/*v*) solution [36]. The X-ray crystal data (Figure 1) indicate that the existence of intermolecular NH-N hydrogen bonds promotes the formation of a **2a** dimer. The length of the NH–N hydrogen bond in the **2a** dimer is 2.103 Å, and the N-N distance of the NH–N hydrogen bond is 2.845 Å.

#### 2.3.2. DFT Calculation of Dimeric Species of **2a** and Its Tautomer

**Chart 2:**
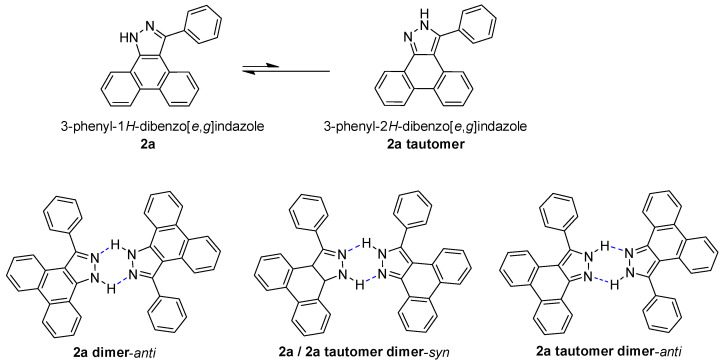
Structures of the expected three types of dimeric species in solution. The blue dashed lines show the NH–N hydrogen bonds.

**Figure 2:**
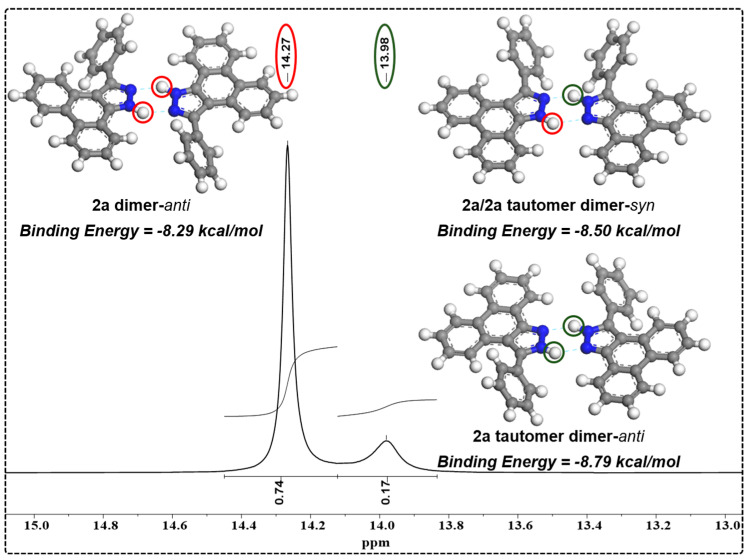
The calculation results of the binding energies of two types of dimeric species of **2a** in DMSO-*d*_6_. The calculations were performed using the Gaussian 09 program at a B3LYP-D3(BJ)/ma-TZVP level. The blue dashed lines show the NH–N hydrogen bonds. The red circles show N-H hydrogen atoms and peak of **2a**, when green circles show that of **2a** tautomer.

In a DMSO-*d*_6_ solvent, two types of proton peaks assigned to the N-H bond were observed in the ^1^H NMR spectra of **2a**, **2d**, **2f**, **2h**, **2i**, **2j**, **2l**, **2n**, **2o**, and **2p**. On the basis of a DFT calculation, it is favorable to form the dimeric species with a definitely lower energy than that of the sum of the two isolated monomers resulted from the hydrogen bond in the solution; thus, there are expected to be three types of dimers, as shown in Chart 2, including a **2a** dimer-*anti*, a **2a**/**2a** tautomer dimer-*syn*, and a tautomer dimer-*anti*, taking two phenyl groups as reference, centered on a six-membered H-bonding chelate. We selected **2a** as the representative sample for calculating the binding energies of these three dimers with DFT using the Gaussian 09 program at a B3LYP-D3(BJ)/ma-TZVP [37,38,39] level. An SMD solvation model was employed with the default settings. The basis set superposition error (BSSE) was corrected using the counterpoise (CP) method of Boys and Bernardi [40]. The calculation results disclose that the binding energies for the formation of the **2a** dimer-*anti*, **2a**/**2a** tautomer dimer-*syn*, and tautomer dimer-*anti* are −8.29 kcal/mol, −8.50 kcal/mol, and −8.79 kcal/mol, respectively (Figure 2). Considering the slight differences of binding energies among these three types of dimers, the observed two types of N-H signals in the ^1^H NMR spectra in DMSO-*d*_6_ are assigned to N-H hydrogen atoms in **2a** (14.27 ppm) and **2a** tautomer (13.98 ppm) in dimeric species, respectively

#### 2.3.3. Temperature Gradient Experiment of Compound **2a** in DMSO-*d*_6_

**Figure 3:**
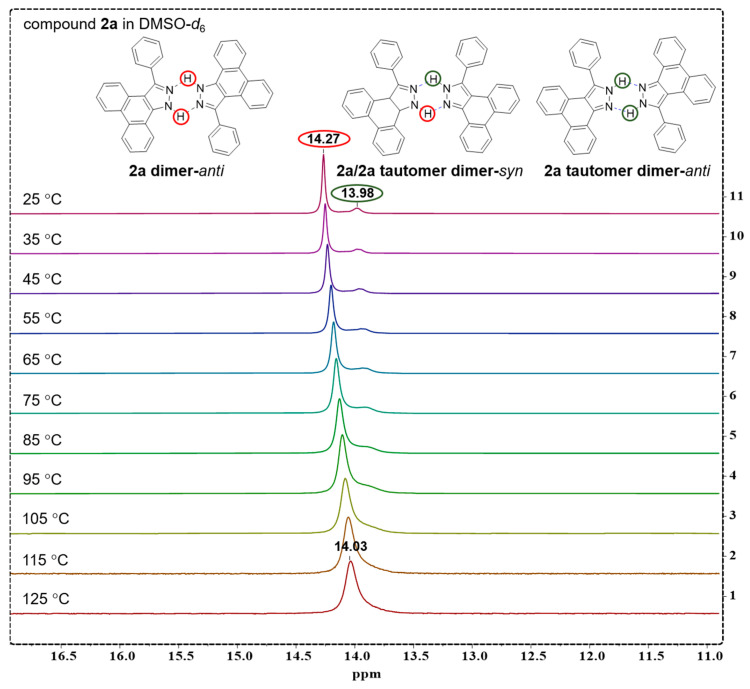
^1^H NMR spectra of compound **2a** recorded in DMSO-*d*_6_ with increasing temperature from 25 °C to 125 °C. The red circles show N-H hydrogen atoms and peak of **2a**, when green circles show that of **2a** tautomer.

A temperature gradient experiment of compound **2a** in DMSO-*d*_6_ was performed from 25 °C to 125 °C; the corresponding ^1^H NMR spectra are recorded in Figure 3. With the temperature increasing, it is shown that two ^1^H sharp signals gradually coalesced into a unique, broad signal at 14.03 ppm at 125 °C, which results from the fast chemical exchange of **2a** and **2a** tautomer at relatively higher temperature.

## 3. Materials and Methods

### 3.1. Materials

All commercially available reagents, solvents, and metal salts are analytically pure and were used without further purification. 1-bromo-2-iodobenzene (CAS 583-55-1), 2-bromo-1-iodo-4-methylbenzene (CAS 71838-16-9), 2-bromo-4-chloro-1-iodobenzene (CAS 31928-44-6), 2-bromo-1-iodo-4-(trifluoromethyl)benzene (CAS 481075-58-5), 3-bromo-2-iodopyridine (CAS 408502-43-2), 4-bromo-3-iodopyridine (CAS 917969-51-8), 3-bromo-4-iodopyridine (CAS 89167-19-1), ethynylbenzene (CAS 536-74-3), 1-ethynyl-4-methoxybenzene (CAS 768-60-5), 1-ethynyl-4-methylbenzene (CAS 766-47-2), 1-ethynyl-4-fluorobenzene (CAS 766-98-3), 1-chloro-4-ethynylbenzene (CAS 873-73-4), 2-ethynylpyridine (CAS 1945-84-2), 2-ethynylthiophene (CAS 4298-52-6), ethynyltriisopropylsilane (CAS 89343-06-6), (2-formylphenyl)boronic acid (CAS 40138-16-7), (4-chloro-2-formylphenyl)boronic acid (CAS 913835-76-4), 4-methylbenzenesulfonohydrazide (CAS 1576-35-8), and dichloroditriphenylphosphor palladium (CAS 13965-03-2) were purchased from Bidepharm; tetrahydrofuran (CAS 109-99-9), triethylamine (CAS 121-44-8), and methyl alcohol (CAS 67-56-1) were purchased from Aladdin; hept-1-yne (CAS 628-71-7), potassium fluoride (CAS 7789-23-3), and 1,4-dioxane (CAS 123-91-1) were purchased from Meryer (Shanghai, China); cuprous iodide (CAS 7681-65-4) and lithium t-butoxide (CAS 1907-33-1) were purchased from Macklin (Shanghai, China); the H_2_O used was ultrapure water.

### 3.2. General Methods

Column chromatography was performed on silica gel (300–400 mesh). Thin-layer chromatography (TLC) was performed on 0.2 mm silica gel-coated glass sheets. The NMR spectra were recorded on a JEOL ECS-400 instrument operating at 400 and 100 MHz for the ^1^H and ^13^C nuclei, respectively. All chemical shifts (*δ*_H_, *δ*_C_, *δ*_F_) are given in parts per million (ppm); all homocoupling patterns (^n^*J*_H,H_) are given in hertz (Hz). No TMS was added; the chemical shifts were measured against the solvent peak taken as a reference signal; CDCl_3_, *δ*_H_ = 7.26 ppm, and *δ*_C_ = 77.16 ppm; DMSO-*d*_6_, *δ*_H_ = 2.50 ppm, and *δ*_C_ = 39.52 ppm. The high-resolution mass spectroscopy (HRMS) spectra were obtained using high-resolution mass spectrometers with an electrospray ionization (ESI) source. The single-crystal X-ray diffraction data were obtained using a SuperNova (Agilent Technologies, Oxfordshire, UK) diffractometer with a Cu K_α_ radiation at a low temperature (173.15 K). All the NMR charts for the prepared starting materials and the products are reported in the Supplementary Materials.

### 3.3. General Procedure for the Preparation of 2′-Alkynyl-biaryl-2-aldehydes ***1a***–***p***

(1) A THF (5.0 mL) and Et_3_N (5.0 mL) solution containing 1-bromo-2-iodobenzenes (2.0 mmol), CuI (5.0 mol%, 19.0 mg, 0.1 mmol), and PdCl_2_(PPh_3_)_2_ (5.0 mol%, 70.2 mg, 0.1 mmol) in a 25 mL screw-capped thick-walled Pyrex tube with stirring under N_2_ was dropwise added to terminal alkynes (2.4 mmol) at room temperature over 5 min. The obtained mixture was then stirred at room temperature under N_2_ for 12 h. After the reaction was completed (TLC monitoring, eluent pure petroleum ether), the reaction mixture was filtrated through a short pad of celite. The solution was then concentrated under reduced pressure to remove the volatiles, and the crude residue was purified using column chromatography on silica gel (eluent pure petroleum ether) to obtain the desired compounds **S_a_**–**S_p_** (checked with GC-MS) in 75–95% yields.

(2) A 1,4-dioxane (10.0 mL) and H_2_O (1.0 mL) solution containing **S** (1.5 mmol), phenylboronic acids (1.65 mmol), PdCl_2_(PPh_3_)_2_ (5.0 mol%, 52.7 mg, 0.075 mmol), and KF (261.0 mg, 4.5 mmol) in a 25 mL screw-capped thick-walled Pyrex tube was stirred under N_2_ at 100 °C in an oil bath for 12 h. After the reaction was completed, the reaction mixture was cooled to room temperature (TLC monitoring, eluent petroleum ether/ethyl acetate, 15/1 *v*/*v*) and filtrated through a short pad of celite. The solution was then concentrated under reduced pressure to remove the volatiles, and the crude residue was purified using column chromatography on silica gel (eluent petroleum ether/ethyl acetate, gradient mixture ratio from 30/1 to 15/1 *v*/*v*) to afford product **1a**–**p** in 24–94% yields.

Compounds **1a**, **1c**, **1e**, **1f**, **1g** are known compounds, which were confirmed by their ^1^H NMR and ^13^C NMR spectroscopic data [41].

### 3.4. Analytical Data of Compound ***1a***–***p***

2′-(Phenylethynyl)-(1,1′-biphenyl)-2-carbaldehyde (**1a**). Pale yellow oil (355 mg, 1.26 mmol, 84% yield). Rf = 0.40 (petroleum ether/ethyl acetate, 10/1 *v*/*v*). 1H NMR (400 MHz, CDCl_3_) *δ*_H_ 9.94 (s, 1H), 8.09 (dd, ^3^*J*_H,H_ = 7.8 Hz, ^4^*J*_H,H_ = 1.5 Hz, 1H), 7.68–7.64 (m, 2H), 7.54 (t, ^3^*J*_H,H_ = 7.6 Hz, 1H), 7.46–7.38 (m, 4H), 7.25–7.22 (m, 4H), 7.17–7.15 (m, 2H) ppm. ^13^C NMR (100 MHz, CDCl_3_) *δ*_C_ 192.0, 144.4, 140.4, 134.3, 133.6, 132.1, 131.4, 130.4, 128.6, 128.4, 128.3, 127.0, 123.8, 122.8, 93.9, 88.3 ppm.

2′-[(4-Methoxyphenyl)ethynyl]-(1,1′-biphenyl)-2-carbaldehyde (**1b**). Yellow oil (332 mg, 1.07 mmol, 71% yield). *R*_f_ = 0.55 (petroleum ether/ethyl acetate, 10/1 *v*/*v*). ^1^H NMR (400 MHz, CDCl_3_) *δ*_H_ 9.93 (s, 1H), 8.08 (dd, ^3^*J*_H,H_ = 7.9 Hz, ^4^*J*_H,H_ = 1.5 Hz, 1H), 7.66–7.59 (m, 2H), 7.52 (dd app. t, ^3^*J*_H,H_ = 7.5 Hz, 1H), 7.43–7.36 (m, 4H), 7.12–7.08 (m, 2H), 6.77–6.74 (m, 2H), 3.74 (s, 3H) ppm. ^13^C NMR (100 MHz, CDCl_3_) *δ*_C_ 191.9, 159.8, 144.5, 140.1, 134.3, 133.5, 132.8, 131.8, 131.4, 130.3, 128.3, 128.2, 128.2, 126.8, 124.1, 114.9, 114.0, 94.0, 87.1, 55.3 ppm. HRMS (ESI IT-TOF) *m*/*z* [M + H]^+^ Calcd. for C_22_H_17_O_2_ 313.1223, found 313.1223.

2′-(p-Tolylethynyl)-(1,1′-biphenyl)-2-carbaldehyde (**1c**). Pale yellow oil (417 mg, 1.41 mmol, 94% yield). *R*_f_ = 0.50 (petroleum ether/ethyl acetate, 10/1 *v*/*v*). ^1^H NMR (400 MHz, CDCl_3_) *δ*_H_ 9.93 (s, 1H), 8.08 (dd, ^3^*J*_H,H_ = 7.7 Hz, ^4^*J*_H,H_ = 1.5 Hz, 1H), 7.66–7.61 (m, 2H), 7.52 (t, ^3^*J*_H,H_ = 7.6 Hz, 1H), 7.44–7.36 (m, 4H), 7.07–7.02 (m, 4H), 2.29 (s, 3H) ppm. ^13^C NMR (100 MHz, CDCl_3_) *δ*_C_ 191.9, 144.4, 140.3, 138.7, 134.3, 133.5, 132.0, 131.4, 131.3, 130.3, 129.1, 128.3, 128.3, 126.9, 124.0, 119.7, 94.1, 87.7, 21.6 ppm.

2′-[(4-Fluorophenyl)ethynyl]-(1,1′-biphenyl)-2-carbaldehyde (**1d**). Pale yellow oil (333 mg, 1.11 mmol, 74% yield). *R*_f_ = 0.40 (petroleum ether/ethyl acetate, 10/1 *v*/*v*). ^1^H NMR (400 MHz, CDCl_3_) *δ*_H_ 9.93 (s, 1H), 8.08 (dd, ^3^*J*_H,H_ = 7.8 Hz, ^4^*J*_H,H_ = 1.5 Hz, 1H), 7.67–7.61 (m, 2H), 7.53 (t, ^3^*J*_H,H_ = 7.6 Hz, 1H), 7.46–7.38 (m, 4H), 7.16–7.11 (m, 2H), 6.95–6.90 (m, 2H) ppm. ^13^C NMR (100 MHz, CDCl_3_) *δ*_C_ 191.9, 162.7 (d, *J* = 249.6 Hz), 144.3, 140.3, 134.3, 133.6, 133.3 (d, *J* = 8.3 Hz), 132.0, 131.4, 130.3, 128.6, 128.3, 126.9, 123.6, 118.8 (d, *J* = 3.6 Hz), 115.7 (d, *J* = 22.2 Hz), 92.8, 88.0. HRMS (ESI IT-TOF) *m*/*z* [M + H]^+^ Calcd. for C_21_H_14_FO 301.1023, found 301.1023.

2′-[(4-Chlorophenyl)ethynyl]-(1,1′-biphenyl)-2-carbaldehyde (**1e**). Pale yellow oil (436 mg, 1.38 mmol, 92% yield). *R*_f_ = 0.40 (petroleum ether/ethyl acetate, 10/1 *v*/*v*). ^1^H NMR (400 MHz, CDCl_3_) *δ*_H_ 9.92 (s, 1H), 8.08 (dd, ^3^*J*_H,H_ = 7.8 Hz, ^4^*J*_H,H_ = 1.5 Hz, 1H), 7.69–7.62 (m, 2H), 7.55 (t, ^3^*J*_H,H_ = 7.5 Hz, 1H), 7.49–7.40 (m, 4H), 7.22–7.19 (m, 2H), 7.09–7.07 (m, 2H) ppm. ^13^C NMR (100 MHz, CDCl_3_) *δ*_C_ 191.9, 144.3, 140.5, 134.6, 134.3, 133.6, 132.6, 132.1, 131.4, 130.4, 128.8, 128.8, 128.4, 128.4, 127.0, 123.5, 121.3, 92.7, 89.3 ppm.

5′-Methyl-2′-(phenylethynyl)-(1,1′-biphenyl)-2-carbaldehyde (**1f**). Pale yellow oil (404 mg, 1.36 mmol, 91% yield). *R*_f_ = 0.40 (petroleum ether/ethyl acetate, 10/1 *v*/*v*). ^1^H NMR (400 MHz, CDCl_3_) *δ*_H_ 9.94 (s, 1H), 8.08 (dd, ^3^*J*_H,H_ = 7.9 Hz, ^4^*J*_H,H_ = 1.6 Hz, 1H), 7.66–7.61 (m, 1H), 7.53–7.49 (m, 2H), 7.42 (d, ^3^*J*_H,H_ = 7.5 Hz, 1H), 7.23–7.20 (m, 5H), 7.16–7.14 (m, 2H), 2.41 (s, 3H) ppm. ^13^C NMR (100 MHz, CDCl_3_) *δ*_C_ 192.0, 144.5, 140.3, 138.8, 134.3, 133.5, 131.9, 131.3, 131.3, 131.1, 129.1, 128.3, 128.2, 126.9, 123.0, 120.8, 93.1, 88.5, 21.6 ppm.

5′-Chloro-2′-(phenylethynyl)-(1,1′-biphenyl)-2-carbaldehyde (**1g**). Pale yellow solid (374 mg, 1.18 mmol, 79% yield). *R*_f_ = 0.40 (petroleum ether/ethyl acetate, 10/1 *v*/*v*). m.p. 83.7–84.2 °C. ^1^H NMR (400 MHz, CDCl_3_) *δ*_H_ 9.93 (s, 1H), 8.09 (dd, ^3^*J*_H,H_ = 7.8 Hz, ^4^*J*_H,H_ = 1.5 Hz, 1H), 7.67 (td, ^3^*J*_H,H_ = 7.4 Hz, ^4^*J*_H,H_ = 1.5 Hz, 1H), 7.58–7.54 (m, 2H), 7.42–7.39 (m, 3H), 7.26–7.21(m, 4H), 7.16–7.13 (m, 2H) ppm. ^13^C NMR (100 MHz, CDCl_3_) *δ*_C_ 191.4, 142.9, 142.1, 134.5, 134.3, 133.8, 133.1, 131.4, 131.2, 130.3, 128.8, 128.6, 128.4, 127.3, 122.5, 94.8, 87.3 ppm.

2′-(Phenylethynyl)-5′-(trifluoromethyl)-(1,1′-biphenyl)-2-carbaldehyde (**1h**). Pale yellow solid (483 mg, 1.38 mmol, 92% yield). *R*_f_ = 0.40 (petroleum ether/ethyl acetate, 10/1 *v*/*v*). m.p. 79.5–80.1 °C. ^1^H NMR (400 MHz, CDCl_3_) *δ*_H_ 9.92 (s, 1H), 8.11 (dd, ^3^*J*_H,H_ = 7.8 Hz, ^4^*J*_H,H_ = 1.5 Hz, 1H), 7.75–7.67 (m, 4H), 7.58 (t, ^3^*J*_H,H_ = 7.6 Hz, 1H), 7.41 (d, ^3^*J*_H,H_ = 7.8 Hz, 1H), 7.28–7.26 (m, 3H), 7.18–7.15 (m, 2H) ppm. ^13^C NMR (100 MHz, CDCl_3_) *δ*_C_ 191.1, 142.7, 141.2, 134.3, 133.9, 132.4, 131.6, 131.3, 130.3 (q, *J* = 32.7 Hz), 129.1, 129.0, 128.5, 127.6, 127.5, 126.9 (q, *J* = 3.8 Hz), 125.1 (q, *J* = 3.5 Hz), 123.9 (q, *J* = 273.7 Hz), 122.1, 96.4, 87.1 ppm. ^19^F NMR (376 MHz, Chloroform-*d*) *δ* −62.53. HRMS (ESI IT-TOF) *m*/*z* [M + H]^+^ Calcd. for C_22_H_14_F_3_O 351.0991, found 351.0991.

2′-(Pyridin-2-ylethynyl)-(1,1′-biphenyl)-2-carbaldehyde (**1i**). Pale yellow solid (378 mg, 1.34 mmol, 89% yield). *R*_f_ = 0.40 (petroleum ether/ethyl acetate, 10/1 *v*/*v*). m.p. 104.6–104.9 °C. ^1^H NMR (400 MHz, CDCl_3_) *δ*_H_ 9.95 (s, 1H), 8.50 (d, ^3^*J*_H,H_ = 3.3 Hz, 1H), 8.09 (d, ^3^*J*_H,H_ = 7.7 Hz, 1H), 7.74 (dd, ^3^*J*_H,H_ = 7.3 Hz, ^4^*J*_H,H_ = 1.7 Hz, 1H), 7.66 (td, ^3^*J*_H,H_ = 7.5 Hz, ^4^*J*_H,H_ = 1.4 Hz, 1H), 7.55–7.39 (m, 6H), 7.15–7.11 (m, 1H), 7.00 (d, ^3^*J*_H,H_ = 7.8 Hz, 1H) ppm. ^13^C NMR (100 MHz, CDCl_3_) *δ*_C_ 191.6, 149.9, 143.9, 142.8, 140.6, 136.0, 134.1, 133.5, 132.6, 131.3, 130.3, 129.1, 128.3, 128.3, 127.1, 126.8, 122.8, 122.6, 92.7, 87.8 ppm. HRMS (ESI IT-TOF) *m*/*z* [M + H]^+^ Calcd. for C_20_H_14_NO 284.1070, found 284.1069.

2′-(Thiophen-2-ylethynyl)-(1,1′-biphenyl)-2-carbaldehyde (**1j**). Pale yellow oil (363 mg, 1.26 mmol, 84% yield). *R*_f_ = 0.40 (petroleum ether/ethyl acetate, 10/1 *v*/*v*). ^1^H NMR (400 MHz, CDCl_3_) *δ*_H_ 9.91 (s, 1H), 8.08 (dd, ^3^*J*_H,H_ = 7.7 Hz, ^4^*J*_H,H_ = 1.5 Hz, 1H), 7.67–7.59 (m, 2H), 7.52 (t, ^3^*J*_H,H_ = 7.6 Hz, 1H), 7.45–7.36 (m, 4H), 7.18 (dd, ^3^*J*_H,H_ = 5.1 Hz, ^4^*J*_H,H_ = 1.2 Hz, 1H), 6.98 (dd, ^3^*J*_H,H_ = 3.7 Hz, ^4^*J*_H,H_ = 1.2 Hz, 1H), 6.88 (dd, ^3^*J*_H,H_ = 5.2 Hz, ^3^*J*_H,H_ = 3.6 Hz, 1H) ppm. ^13^C NMR (100 MHz, CDCl_3_) *δ*_C_ 191.7, 144.1, 140.1, 134.2, 133.5, 132.0, 131.6, 131.3, 130.3, 128.5, 128.3, 128.2, 127.7, 127.1, 127.0, 123.4, 122.6, 92.0, 87.3 ppm. HRMS (ESI IT-TOF) *m*/*z* [M + H]^+^ Calcd. for C_19_H_13_OS 289.0682, found 289.0682.

2′-[(Triisopropylsilyl)ethynyl]-(1,1′-biphenyl)-2-carbaldehyde (**1k**). White solid (391 mg, 1.08 mmol, 72% yield). *R*_f_ = 0.50 (petroleum ether/ethyl acetate, 10/1 *v*/*v*). m.p. 68.0–68.3 °C. ^1^H NMR (400 MHz, CDCl_3_) *δ*_H_ 9.85 (s, 1H), 8.01 (d, ^3^*J*_H,H_ = 7.7 Hz, 1H), 7.61–7.58 (m, 2H), 7.46 (t, ^3^*J*_H,H_ = 7.6 Hz, 1H), 7.41–7.34 (m, 3H), 7.31–7.28 (m, 1H), 0.91 (s, 21H) ppm. ^13^C NMR (100 MHz, CDCl_3_) *δ*_C_ 191.6, 144.6, 140.7, 134.1, 133.4, 132.7, 131.0, 130.1, 128.3, 128.1, 128.0, 127.0, 123.9, 105.2, 95.7, 18.5, 11.1. HRMS (ESI IT-TOF) *m*/*z* [M + H]^+^ Calcd. for C_24_H_31_OSi 363.2139, found 363.2138.

2′-(Hept-1-yn-1-yl)-(1,1′-biphenyl)-2-carbaldehyde (**1l**). Pale yellow oil (99 mg, 0.36 mmol, 24% yield). *R*_f_ = 0.50 (petroleum ether/ethyl acetate, 10/1 *v*/*v*). ^1^H NMR (400 MHz, CDCl_3_) *δ*_H_ 9.85 (s, 1H), 8.03 (d, ^3^*J*_H,H_ = 7.7 Hz, 1H), 7.64–7.60 (m, 1H), 7.50–7.49 (m, 2H), 7.35–7.32 (m, 4H), 7.25 (s, 1H), 2.17–2.13 (s, 2H), 1.33–1.26 (m, 2H), 1.21–1.16 (m, 2H), 1.11–1.05 (m, 2H), 0.83–0.79 (m, 3H) ppm. ^13^C NMR (100 MHz, CDCl_3_) *δ*_C_ 192.0, 144.7, 140.2, 134.1, 133.4, 132.0, 131.2, 130.1, 128.1, 128.0, 127.7, 126.7, 124.5, 95.5, 79.5, 30.8, 27.8, 22.2, 19.3, 14.0 ppm. HRMS (ESI IT-TOF) *m*/*z* [M + H]^+^ Calcd. for C_20_H_21_O 277.1587, found 277.1587.

4-Chloro-2′-[(triisopropylsilyl)ethynyl]-(1,1′-biphenyl)-2-carbaldehyde (**1m**). Pale yellow oil (422 mg, 1.07 mmol, 71% yield). *R*_f_ = 0.50 (petroleum ether/ethyl acetate, 10/1 *v*/*v*). ^1^H NMR (400 MHz, CDCl_3_) *δ*_H_ 9.77 (s, 1H), 7.98 (d, ^3^*J*_H,H_ = 2.6 Hz, 1H), 7.62–7.56 (m, 2H), 7.44–7.37 (m, 2H), 7.34 (d, ^3^*J*_H,H_ = 8.2 Hz, 1H), 7.30–7.28 (m, 1H), 0.92 (s, 21H) ppm. ^13^C NMR (100 MHz, CDCl_3_) *δ*_C_ 190.4, 142.8, 139.5, 135.3, 134.7, 133.3, 132.9, 132.6, 130.0, 128.5, 128.4, 126.9, 124.0, 104.9, 96.4, 18.5, 11.1 ppm. HRMS (ESI IT-TOF) *m*/*z* [M + H]^+^ Calcd. for C_21_H_24_ClOSi 355.1279, found 355.1278.

2-[2-(Phenylethynyl)pyridin-3-yl]benzaldehyde (**1n**). Pale yellow oil (365 mg, 1.29 mmol, 86% yield). *R*_f_ = 0.40 (petroleum ether/ethyl acetate, 10/1 *v*/*v*). ^1^H NMR (400 MHz, CDCl_3_) *δ*_H_ 9.96 (s, 1H), 8.69 (dd, ^3^*J*_H,H_ = 4.7, ^4^*J*_H,H_ = 1.8 Hz, 1H), 8.11 (dd, ^3^*J*_H,H_ = 7.8 Hz, ^4^*J*_H,H_ = 1.4 Hz, 1H), 7.73–7.67 (m, 2H), 7.59 (t, ^3^*J*_H,H_ = 7.5 Hz, 1H), 7.43–7.36 (m, 2H), 7.30–7.19 (m, 5H) ppm. ^13^C NMR (100 MHz, CDCl_3_) *δ*_C_ 190.8, 149.7, 142.8, 141.4, 137.5, 136.6, 134.2, 133.7, 131.7, 131.3, 129.1, 128.9, 128.3, 127.7, 122.6, 121.6, 93.7, 87.7 ppm. HRMS (ESI IT-TOF) *m*/*z* [M + H]^+^ Calcd. for C_20_H_14_NO 284.1070, found 284.1069.

2-[3-(Phenylethynyl)pyridin-4-yl]benzaldehyde (**1o**). Pale yellow oil (386 mg, 1.36 mmol, 91% yield). *R*_f_ = 0.40 (petroleum ether/ethyl acetate, 10/1 *v*/*v*). ^1^H NMR (400 MHz, CDCl_3_) *δ*_H_ 9.93 (s, 1H), 8.86 (s, 1H), 8.65 (d, ^3^*J*_H,H_ = 5.1 Hz, 1H), 8.11 (dd, ^3^*J*_H,H_ = 7.9, 1.5 Hz, 1H), 7.71 (td, ^3^*J*_H,H_ = 7.5 Hz, ^4^*J*_H,H_ = 1.5 Hz, 1H), 7.61 (t, ^3^*J*_H,H_ = 7.7 Hz, 1H), 7.43–7.40 (m, 1H), 7.34 (d, ^3^*J*_H,H_ = 5.1 Hz, 1H), 7.31–7.20 (m, 6H) ppm. ^13^C NMR (100 MHz, CDCl_3_) *δ*_C_ 190.6, 152.5, 148.7, 147.8, 141.1, 133.8, 131.4, 130.7, 129.3, 129.0, 128.4, 127.8, 124.2, 122.0, 120.6, 96.7, 84.9 ppm. HRMS (ESI IT-TOF) *m*/*z* [M + H]^+^ Calcd. for C_20_H_14_NO 284.1070, found 284.1069.

2-[4-(Phenylethynyl)pyridin-3-yl]benzaldehyde (**1p**). Pale yellow oil (378 mg, 1.33 mmol, 89% yield). *R*_f_ = 0.40 (petroleum ether/ethyl acetate, 10/1 *v*/*v*). ^1^H NMR (400 MHz, CDCl_3_) *δ*_H_ 9.95 (s, 1H), 8.67–8.66 (m, 2H), 8.12 (d, ^3^*J*_H,H_ = 7.8 Hz, 1H), 7.71 (td, ^3^*J*_H,H_ = 7.4, ^4^*J*_H,H_ = 1.4 Hz, 1H), 7.60 (t, ^3^*J*_H,H_ = 7.6 Hz, 1H), 7.49 (d, ^3^*J*_H,H_ = 5.1 Hz, 1H), 7.44 (d, ^3^*J*_H,H_ = 7.6 Hz, 1H), 7.32–7.19 (m, 5H) ppm. ^13^C NMR (100 MHz, CDCl_3_) *δ*_C_ 190.8, 150.2, 149.3, 140.1, 135.0, 134.4, 133.8, 131.6, 131.6, 131.4, 129.4, 129.0, 128.4, 127.7, 125.1, 121.5, 98.2, 85.7 ppm. HRMS (ESI IT-TOF) *m*/*z* [M + H]^+^ Calcd. for C_20_H_14_NO 284.1070, found 284.1069.

### 3.5. General Procedure for the Preparation of 1H-Dibenzo[e,g]indazoles ***2a***–***p***

A THF (5.0 mL) solution containing 2′-alkynyl-biaryl-2-aldehydes (**1**, 282.0 mg, 1.0 mmol) and *p*-toluenesulfonhydrazide (204.9 mg, 1.1 mmol) in a 25 mL screw-capped thick-walled Pyrex tube was stirred at 45 °C for 1 h. After the reaction was completed (TLC monitoring, eluent petroleum ether/ethyl acetate, 1/1 *v*/*v*), LiO*^t^*Bu (120.0 mg, 1.5 mmol) and additional THF (2.5 mL) were added, and then the mixture was stirred at 45 °C for 1 h. After the reaction was completed (TLC monitoring, eluent petroleum ether/ethyl acetate, 1/1 *v*/*v*), the crude residue was directly purified using column chromatography on silica gel (eluent petroleum ether/ethyl acetate, gradient mixture ratio from 5/1 to 2/1 *v*/*v*) to afford products **2a**–**p** in 61–93% yields.

### 3.6. Analytical Data of Compound ***2a***–***p***

3-Phenyl-1*H*-dibenzo[*e*,*g*]indazole (**2a**) [30]. White solid (259 mg, 0.88 mmol, 88% yield). *R*_f_ = 0.40 (petroleum ether/ethyl acetate, 1/1 *v*/*v*). m.p. 260.4–260.8 °C. ^1^H NMR (400 MHz, DMSO-*d*_6_) *δ*_H_ 14.24–13.95 (s, 1H), 8.78–8.71 (m, 2H), 8.55 (d, ^3^*J*_H,H_ = 7.8 Hz, 1H), 8.02 (d, ^3^*J*_H,H_ = 8.1 Hz, 1H), 7.74–7.69 (m, 4H), 7.59–7.54 (m, 3H), 7.50–7.40 (m, 2H) ppm. ^13^C NMR (100 MHz, DMSO-*d*_6_) *δ*_C_ 147.3, 137.2, 135.4, 129.6, 128.6, 128.3, 127.5, 127.4, 127.1, 124.9, 124.1, 124.0, 122.6, 122.3, 121.0, 112.5 ppm.

3-(4-Methoxyphenyl)-1*H*-dibenzo[*e*,*g*]indazole (**2b**). White solid (285 mg, 0.88 mmol, 88% yield). *R*_f_ = 0.40 (petroleum ether/ethyl acetate, 1/1 *v*/*v*). m.p. 204.2–204.7 °C. ^1^H NMR (400 MHz, DMSO-*d*_6_) *δ*_H_ 13.97 (s, 1H), 8.71 (dd, ^2^*J*_H,H_ = 16.9 Hz, ^3^*J*_H,H_ = 8.1 Hz, 2H), 8.56 (d, ^3^*J*_H,H_ = 7.7 Hz, 1H), 8.05 (d, ^3^*J*_H,H_ = 7.3 Hz, 1H), 7.74–7.63 (m, 4H), 7.49–7.40 (m, 2H), 7.14 (d, ^3^*J*_H,H_ = 8.4 Hz, 2H), 3.84 (s, 3H) ppm. ^13^C NMR (100 MHz, DMSO-*d*_6_) *δ*_C_ 159.3, 147.2, 137.2, 130.9, 129.6, 127.4, 127.3, 127.1, 124.8, 124.0, 123.9, 122.6, 122.4, 121.1, 114.0, 112.6, 55.1 ppm. HRMS (ESI IT-TOF) *m*/*z* [M + H]^+^ Calcd. for C_22_H_17_N_2_O 325.1335, found 325.1334.

3-(*p*-Tolyl)-1*H*-dibenzo[*e*,*g*]indazole (**2c**). White solid (262 mg, 0.85 mmol, 85% yield). *R*_f_ = 0.40 (petroleum ether/ethyl acetate, 1/1 *v*/*v*). m.p. 199.6–200.0 °C. ^1^H NMR (400 MHz, DMSO-*d*_6_) *δ*_H_ 11.79 (s, 1H), 8.71 (dd, ^2^*J*_H,H_ = 17.1 Hz, ^3^*J*_H,H_ = 8.1 Hz, 2H), 8.57 (d, ^3^*J*_H,H_ = 7.7 Hz, 1H), 8.06 (d, ^3^*J*_H,H_ = 7.7 Hz, 1H), 7.73 (t, ^3^*J*_H,H_ = 7.5 Hz, 1H), 7.66 (t, ^3^*J*_H,H_ = 7.5 Hz, 1H), 7.61 (d, ^3^*J*_H,H_ = 7.8 Hz, 2H), 7.48–7.36 (m, 4H), 2.40 (s, 3H) ppm. ^13^C NMR (100 MHz, DMSO-*d*_6_) *δ*_C_ 145.1, 139.0, 137.8, 131.4, 129.7, 129.4, 129.2, 127.5, 127.4, 127.3, 127.1, 125.6, 124.9, 124.1, 123.9, 122.6, 122.3, 112.3, 20.9 ppm. HRMS (ESI IT-TOF) *m*/*z* [M + H]^+^ Calcd. for C_22_H_17_N_2_ 309.1386, found 309.1385.

3-(4-Fluorophenyl)-1*H*-dibenzo[*e*,*g*]indazole (**2d**). White solid (262 mg, 0.84 mmol, 84% yield). *R*_f_ = 0.40 (petroleum ether/ethyl acetate, 1/1 *v*/*v*). m.p. 235.7–236.2 °C. ^1^H NMR (400 MHz, DMSO-*d*_6_) *δ*_H_ 14.31–14.03 (s, 1H), 8.70–8.58 (m, 3H), 8.00 (s, 1H), 7.80–7.62 (m, 4H), 7.43–7.39 (m, 4H) ppm. ^13^C NMR (100 MHz, DMSO-*d*_6_) *δ*_C_ 162.2 (d, *J* = 245.1 Hz), 146.4, 137.3, 131.8, 131.7, 131.6, 129.6, 127.4, 127.3, 127.1, 124.9, 124.1, 124.0, 122.6, 122.4, 121.0, 115.5 (d, *J* = 21.5 Hz), 112.6 ppm. HRMS (ESI IT-TOF) *m*/*z* [M + H]^+^ Calcd. for C_21_H_14_FN_2_ 313.1136, found 313.1134.

3-(4-Chlorophenyl)-1*H*-dibenzo[*e*,*g*]indazole (**2e**). White solid (262 mg, 0.80 mmol, 80% yield). *R*_f_ = 0.40 (petroleum ether/ethyl acetate, 1/1 *v*/*v*). m.p. 265.3–265.9 °C. ^1^H NMR (400 MHz, DMSO-*d*_6_) *δ*_H_ 14.18 (s, 1H), 8.76 (dd, ^2^*J*_H,H_ = 17.0 Hz, ^3^*J*_H,H_ = 8.0 Hz, 2H), 8.53 (dd, ^3^*J*_H,H_ = 7.7 Hz, ^4^*J*_H,H_ = 1.7 Hz, 1H), 7.96 (d, ^3^*J*_H,H_ = 7.5 Hz, 1H), 7.76–7.68 (m, 4H), 7.66–7.64 (m, 2H), 7.53–7.44 (m, 2H) ppm. ^13^C NMR (100 MHz, DMSO-*d*_6_) *δ*_C_ 144.8, 138.5, 133.6, 133.3, 131.3, 129.6, 128.7, 127.6, 127.4, 127.4, 127.1, 127.1, 125.0, 124.1, 123.9, 122.6, 122.4, 121.7, 112.5 ppm. HRMS (ESI IT-TOF) *m*/*z* [M + H]^+^ Calcd. for C_21_H_14_ClN_2_ 329.0840, found 329.0838.

6-Methyl-3-phenyl-1*H*-dibenzo[*e*,*g*]indazole (**2f**). White solid (265 mg, 0.86 mmol, 86% yield). *R*_f_ = 0.40 (petroleum ether/ethyl acetate, 1/1 *v*/*v*). m.p. 235.6–235.9 °C. ^1^H NMR (400 MHz, DMSO-*d*_6_) *δ*_H_ 14.22–13.93 (s, 1H), 8.76–8.52 (m, 3H), 7.94 (d, ^3^*J*_H,H_ = 8.3 Hz, 1H), 7.76–7.52 (m, 7H), 7.19 (d, ^3^*J*_H,H_ = 8.4 Hz, 1H), 2.44 (s, 3H) ppm. ^13^C NMR (100 MHz, DMSO-*d*_6_) *δ*_C_ 147.1, 137.0, 135.6, 134.0, 129.6, 129.5, 128.5, 128.4, 128.2, 127.5, 127.3, 127.1, 124.8, 123.9, 122.5, 122.3, 121.1, 112.6, 21.2 ppm. HRMS (ESI IT-TOF) *m*/*z* [M + H]^+^ Calcd. for C_22_H_17_N_2_ 309.1386, found 309.1385.

6-Chloro-3-phenyl-1*H*-dibenzo[*e*,*g*]indazole (**2g**). White solid (276 mg, 0.84 mmol, 84% yield). *R*_f_ = 0.40 (petroleum ether/ethyl acetate, 1/1 *v*/*v*). m.p. 286.9–287.3 °C. ^1^H NMR (400 MHz, DMSO-*d*_6_) *δ*_H_ 13.77 (s, 1H), 8.73–8.69 (m, 2H), 8.51 (d, ^3^*J*_H,H_ = 7.6 Hz, 1H), 7.92 (d, ^3^*J*_H,H_ = 8.6 Hz, 1H), 7.75–7.64 (m, 4H), 7.60–7.52 (m, 3H), 7.40 (dd, ^3^*J*_H,H_ = 8.6 Hz, ^4^*J*_H,H_ = 2.1 Hz, 1H) ppm. ^13^C NMR (100 MHz, DMSO-*d*_6_) *δ*_C_ 129.8, 129.5, 129.2, 128.7, 128.5, 128.1, 127.4, 127.0, 125.8, 124.3, 124.2, 123.6, 122.3, 111.7. HRMS (ESI IT-TOF) *m*/*z* [M + H]^+^ Calcd. for C_21_H_14_ClN_2_ 329.0840, found 329.0838.

3-Phenyl-6-(trifluoromethyl)-1*H*-dibenzo[*e*,*g*]indazole (**2h**). White solid (300 mg, 0.83 mmol, 83% yield). *R*_f_ = 0.40 (petroleum ether/ethyl acetate, 1/1 *v*/*v*). m.p. 276.9–277.3 °C. ^1^H NMR (400 MHz, DMSO-*d*_6_) *δ*_H_ 14.29–14.10 (s, 1H), 8.88–8.84 (m, 1H), 8.72–8.65 (m, 1H), 8.55–8.47 (m, 1H), 8.07–8.02 (m, 1H), 7.71–7.51 (m, 8H) ppm. ^13^C NMR (100 MHz, DMSO-*d*_6_) *δ*_C_ 147.7, 138.0, 135.0, 129.8, 129.5, 128.8, 128.6, 128.4, 128.1, 127.6, 127.0, 126.0, 125.0 (q, *J* = 31.7 Hz), 124.1, 123.3 (d, *J* = 6.7 Hz), 122.8, 122.3, 121.1 (d, *J* = 15.7 Hz), 111.7 ppm. ^19^F NMR (376 MHz, DMSO-*d*_6_) *δ* −60.08. HRMS (ESI IT-TOF) *m*/*z* [M + H]^+^ Calcd. for C_22_H_14_F_3_N_2_ 363.1104, found 363.1102.

3-(Pyridin-2-yl)-1*H*-dibenzo[*e*,*g*]indazole (**2i**). White solid (230 mg, 0.78 mmol, 78% yield). *R*_f_ = 0.40 (petroleum ether/ethyl acetate, 1/1 *v*/*v*). m.p. 209.3–209.7 °C. ^1^H NMR (400 MHz, DMSO-*d*_6_) *δ*_H_ 14.44–14.28 (s, 1H), 9.04–9.02 (m, 1H), 8.86 (d, ^3^*J*_H,H_ = 4.9 Hz, 1H), 8.79–8.58 (m, 3H), 8.06–7.97 (m, 2H), 7.78–7.67 (m, 2H), 7.52–7.49 (m, 3H) ppm. ^13^C NMR (100 MHz, DMSO-*d*_6_) *δ*_C_ 154.3, 148.8, 147.1, 137.8, 137.0, 129.7, 127.5, 127.5, 127.4, 127.3, 127.0, 125.6, 125.2, 124.3, 124.0, 123.7, 123.1, 122.3, 120.9, 113.5. HRMS (ESI IT-TOF) *m*/*z* [M + H]^+^ Calcd. for C_20_H_14_N_3_ 296.1182, found 296.1181.

3-(Thiophen-2-yl)-1*H*-dibenzo[*e*,*g*]indazole (**2j**). White solid (273 mg, 0.91 mmol, 91% yield). *R*_f_ = 0.40 (petroleum ether/ethyl acetate, 1/1 *v*/*v*). m.p. 255.4–255.9 °C. ^1^H NMR (400 MHz, DMSO-*d*_6_) *δ*_H_ 14.40–14.17 (s, 1H), 8.71–8.56 (m, 3H), 8.34–8.31 (m, 1H), 7.76–7.73 (m, 2H), 7.66 (t, ^3^*J*_H,H_ = 7.7 Hz, 1H), 7.56 (d, ^3^*J*_H,H_ = 3.6 Hz, 1H), 7.52–7.46 (m, 2H), 7.32–7.30 (m, 1H) ppm. ^13^C NMR (100 MHz, DMSO-*d*_6_) *δ*_C_ 140.4, 137.4, 136.1, 129.6, 128.0, 127.7, 127.5, 127.3, 127.1, 126.9, 125.1, 124.1, 124.0, 122.6, 122.3, 120.8, 113.2. HRMS (ESI IT-TOF) *m*/*z* [M + H]^+^ Calcd. for C_19_H_13_N_2_S 301.0794, found 301.0793.

3-(Triisopropylsilyl)-1*H*-dibenzo[*e*,*g*]indazole (**2k**). White solid (348 mg, 0.93 mmol, 93% yield). *R*_f_ = 0.40 (petroleum ether/ethyl acetate, 1/1 *v*/*v*). m.p. 87.8–88.3 °C. ^1^H NMR (400 MHz, CDCl_3_) *δ*_H_ 12.12 (s, 1H), 8.75 (d, ^3^*J*_H,H_ = 7.6 Hz, 1H), 8.58–8.53 (m, 2H), 8.27 (d, ^3^*J*_H,H_ = 7.8 Hz, 1H), 7.64–7.46 (m, 4H), 1.78 (hept, ^3^*J*_H,H_ = 7.5 Hz, 3H), 1.12 (d, ^3^*J*_H,H_ = 7.7 Hz, 18H) ppm. ^13^C NMR (100 MHz, CDCl_3_) *δ*_C_ 130.5, 129.1, 128.7, 127.3, 127.2, 126.5, 126.0, 125.3, 124.0, 123.6, 123.4, 123.2, 18.8, 12.7 ppm. HRMS (ESI IT-TOF) *m*/*z* [M + H]^+^ Calcd. for C_24_H_31_N_2_Si 375.2251, found 375.2251.

3-Pentyl-1*H*-dibenzo[*e*,*g*]indazole (**2l**). White solid (176 mg, 0.61 mmol, 61% yield). *R*_f_ = 0.40 (petroleum ether/ethyl acetate, 1/1 *v*/*v*). m.p. 188.5–188.9 °C. ^1^H NMR (400 MHz, DMSO-*d*_6_) *δ*_H_ 13.69–13.49 (s, 1H), 8.78–8.67 (m, 2H), 8.44 (d, ^3^*J*_H,H_ = 7.5 Hz, 1H), 8.24–8.15 (m, 1H), 7.69–7.52 (m, 4H), 3.19 (t, ^3^*J*_H,H_ = 7.6 Hz, 2H), 1.82–1.80 (m, 2H), 1.42–1.32 (m, 4H), 0.87 (t, ^3^*J*_H,H_ = 7.1 Hz, 3H) ppm. ^13^C NMR (100 MHz, DMSO-*d*_6_) *δ*_C_ 147.3, 137.1, 129.4, 127.6, 127.3, 127.1, 124.4, 124.0, 123.1, 122.2, 121.1, 112.4, 31.2, 28.9, 27.7, 21.9, 13.9 ppm. HRMS (ESI IT-TOF) *m*/*z* [M + H]^+^ Calcd. for C_20_H_21_N_2_ 289.1699, found 289.1698.

10-Chloro-3-(triisopropylsilyl)-1*H*-dibenzo[*e*,*g*]indazole (**2m**). White solid (335 mg, 0.82 mmol, 82% yield). *R*_f_ = 0.40 (petroleum ether/ethyl acetate, 1/1 *v*/*v*). m.p. 191.1–191.7 °C. ^1^H NMR (400 MHz, CDCl_3_) *δ*_H_ 11.96 (s, 1H), 8.68 (s, 1H), 8.52 (d, ^3^*J*_H,H_ = 7.9 Hz, 1H), 8.47 (d, ^3^*J*_H,H_ = 9.0 Hz, 1H), 8.23 (d, ^3^*J*_H,H_ = 7.7 Hz, 1H), 7.58–7.50 (m, 3H), 1.76 (hept, ^3^*J*_H,H_ = 7.5 Hz, 3H), 1.15 (d, ^3^*J*_H,H_ = 7.6 Hz, 18H) ppm. ^13^C NMR (100 MHz, DMSO-*d*_6_) *δ*_C_ 133.3, 129.0, 128.7, 128.5, 127.6, 126.9, 125.7, 125.5, 125.1, 124.0, 123.9, 122.8, 18.9, 12.7 ppm. HRMS (ESI IT-TOF) *m*/*z* [M + H]^+^ Calcd. for C_24_H_30_ClN_2_Si 409.1861, found 409.1860.

3-Phenyl-1*H*-benzo[*f*]pyrazolo[3,4-*h*]quinoline (**2n**). White solid (260 mg, 0.88 mmol, 88% yield). *R*_f_ = 0.40 (petroleum ether/ethyl acetate, 1/1 *v*/*v*). m.p. 265.7–266.2 °C. ^1^H NMR (400 MHz, DMSO-*d*_6_) *δ*_H_ 14.39–14.16 (s, 1H), 9.02–8.92 (m, 1H), 8.78–8.56 (m, 3H), 8.29–8.14 (m, 2H), 7.79–7.62 (m, 2H), 7.55–7.42 (m, 4H) ppm. ^13^C NMR (100 MHz, DMSO-*d*_6_) *δ*_C_ 148.6, 148.2, 147.4, 145.9, 144.6, 139.9, 139.7, 134.6, 131.7, 130.1, 129.9, 129.6, 128.9, 128.5, 128.0, 127.9, 127.6, 127.4, 126.0, 124.1, 123.8, 123.4, 122.4, 122.3, 120.8, 120.5, 120.1, 113.4, 112.5 ppm. HRMS (ESI IT-TOF) *m*/*z* [M + H]^+^ Calcd. for C_20_H_14_N_3_ 296.1182, found 296.1181.

3-Phenyl-1*H*-benzo[*f*]pyrazolo[3,4-*h*]isoquinoline (**2o**). White solid (266 mg, 0.90 mmol, 90% yield). *R*_f_ = 0.40 (petroleum ether/ethyl acetate, 1/1 *v*/*v*). m.p. 277.9–278.4 °C. ^1^H NMR (400 MHz, DMSO-*d*_6_) *δ*_H_ 14.37–14.12 (s, 1H), 9.22 (s, 1H), 8.82–8.72 (m, 1H), 8.56–8.52 (m, 3H), 7.85–7.59 (m, 7H) ppm. ^13^C NMR (100 MHz, DMSO-*d*_6_) *δ*_C_ 146.9, 145.1, 144.1, 137.7, 135.1, 132.5, 129.6, 129.4, 128.7, 128.5, 127.7, 127.5, 124.7, 122.4, 117.5, 110.6. HRMS (ESI IT-TOF) *m*/*z* [M + H]^+^ Calcd. for C_20_H_14_N_3_ 296.1182, found 296.1181.

3-Phenyl-1*H*-benzo[*h*]pyrazolo[4,3-*f*]isoquinoline (**2p**). White solid (221 mg, 0.75 mmol, 75% yield). *R*_f_ = 0.40 (petroleum ether/ethyl acetate, 1/1 *v*/*v*). m.p. 324.3–324.8 °C. ^1^H NMR (400 MHz, DMSO-*d*_6_) *δ*_H_ 14.46–14.20 (s, 1H), 10.06–9.95 (m, 1H), 9.04–8.87 (m, 1H), 8.56–8.48 (m, 2H), 7.86–7.55 (m, 8H). HRMS (ESI IT-TOF) *m*/*z* [M + H]^+^ Calcd for C_20_H_14_N_3_ 296.1182, found 296.1181. The ^13^C NMR spectroscopic data could not be recorded due to the poor solubility in deuterated solvents, such as DMSO-*d*_6_, CDCl_3_.

## 4. Conclusions

In conclusion, we have developed a one-pot two-steps synthetic method towards 3-substituted 1*H*-dibenzo[*e*,*g*]indazoles in good to high yields via a LiO*^t^*Bu-promoted intramolecular cyclization of 2′-alkynyl-biaryl-2-aldehyde *N*-tosylhydrazones under mild conditions. The starting 2′-alkynyl-biaryl-2-aldehyde *N*-tosylhydrazones were prepared in situ through the reactions of 2′-alkynyl-biaryl-2-aldehydes with TsNHNH_2_ (*p*-methylbenzenesulfonohydrazide). In addition, two types of N-H signals were observed in the ^1^H-NMR spectra in DMSO-*d*_6_, which are assigned to hydrogen atoms of N-H in **2a** and **2a** tautomer in their dimeric species, respectively. 

## Figures and Tables

**Table 1:** Optimization of the model’s reaction conditions in the case of compound **2a**.

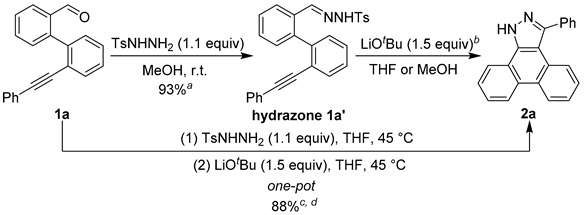			
Entry *^b^*	Solvent	°C/h	Yield of 2a (%) *^d^*
1	THF	100/2	89
2	THF	50/2	88
3	THF	45/2	88
4	THF	35/2	85
5	THF	25/2	77
6	THF	45/1	88
7	MeOH	45/1	68

*^a^* Reaction conditions: **1a** (1.5 mmol), TsNHNH_2_ (1.1 equiv.) in 5.0 mL of MeOH at room temperature. *^b^* Reaction conditions: **1a′** (1.0 mmol), LiO*^t^*Bu (1.5 equiv.) in 5.0 mL of THF or MeOH. *^c^* Reaction conditions: **1a** (1.0 mmol), TsNHNH_2_ (1.1 equiv.) in 5.0 mL of THF at 45 °C for 1 h, then LiO*^t^*Bu (1.5 equiv.) and additional 2.5 mL of THF at 45 °C for 1 h. *^d^* Isolated yields. The green background highlights the optimal conditions.

**Table 2:** Optimal alkali bases used in the synthesis of compound **2a**.

Entry *^a^*	Base	Yield of 2a (%)
1	LiO*^t^*Bu	88
2	NaO*^t^*Bu	81
3	KO*^t^*Bu	85
4	Li_2_CO_3_	9
5	Na_2_CO_3_	9
6	K_2_CO_3_	11
7	Cs_2_CO_3_	14

*^a^* Reaction conditions: **1a** (1.0 mmol), TsNHNH_2_ (1.1 equiv.) in 5.0 mL of THF at 45 °C for 1 h, then base (1.5 equiv.) and additional 2.5 mL of THF at 45 °C for 1 h. The yields were isolated yields. The green background highlights the optimal conditions.

## Data Availability

Data are contained within the article and supplementary materials.

## Supplementary Materials

The following supporting information can be downloaded at https://www.mdpi.com/article/10.3390/molecules28248061/s1: the general procedure for the synthesis of starting materials, the copies of NMR charts of new starting materials, and all products, as well as X-ray structural details of **2a**. References [42,43,44] are cited in the supplementary materials.
