# Supplementary material for: LiOtBu-Promoted Intramolecular 1,3-Dipolar Cycloaddition of the 2′-Alkynyl-biaryl-2-aldehyde N-Tosylhydrazones Approach to 3-Substituted 1H-Dibenzo[e,g]indazoles
Source: Molecules. 2023 Dec 13;28(24):8061. doi: 10.3390/molecules28248061 (PMC10745680; doi:10.3390/molecules28248061)

Supplementary Material

LiO^{t}Bu-promoted Intramolecular 1,3-Dipolar Cycloaddition of the 2'-Alkynyl-biaryl-2-aldehyde *N*-tosylhydrazones Approach to 3-Substituted 1*H*-Dibenzo[*e,g*]indazoles

Jiaying Lv,[†] Ruimao Hua *,[†,‡]

[†] *Department of Chemistry, Tsinghua University, Key Laboratory of Organic Optoelectronics & Molecular Engineering of Ministry of Education, Beijing 100084, China*

[‡] *State Key Laboratory of Chemistry and Utilization of Carbon-Based Energy Resources, College of Chemistry, Xinjiang University, Urumqi 830017, China*

E-mail: ruimao@mail.tsinghua.edu.cn

A. Experimental procedures and characterization data S2

B.1 Preparation of 2'-alkynyl-biaryl-2-aldehydes **1a-1p** S2

B.2 Preparation of 2'-alkynyl-biaryl-2-aldehyde *N*-tosylhydrazone **1a'** S14

B.3 Preparation of 3-substituted 1*H*-dibenzo[*e*,*g*]indazoles **2a-2p** S16

B. Single crystal structures S26

C. Computational details S34

D. NMR charts S43

A. Experimental procedures and characterization data

B-1. Preparation of 2'-alkynyl-biaryl-2-aldehydes 1a-p

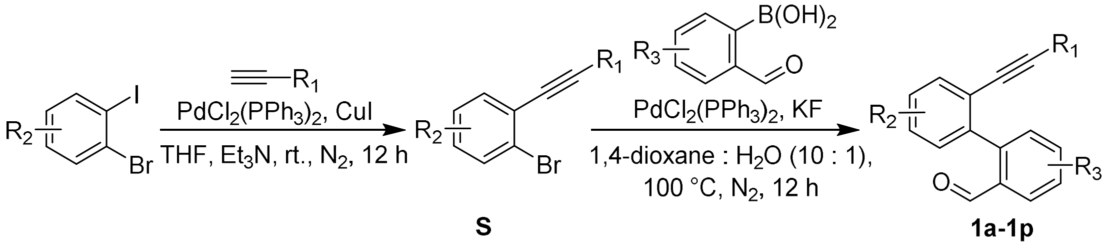

General procedures for the preparation of 2'-cyano-biaryl-2-aldehyde 1a-p:

(1) A THF (5.0 mL) and Et_3N (5.0 mL) solution containing 1-bromo-2-iodobenzenes (2.0 mmol), CuI (5.0 mol%, 19.0mg, 0.1 mmol) and $PdCl_2(PPh_3)_2$ (5.0 mol%, 70.2 mg, 0.1 mmol) in a 25 mL screw-capped thick-walled Pyrex tube with stirring under N_2 was dropwise added terminal alkynes (2.4 mmol) at room temperature over 5 minutes. The obtained mixture was then stirred at room temperature under N_2 for 12 h. After the reaction was completed (TLC monitoring, eluent pure petroleum ether), the reaction mixture was filtrated through a short pad of celite. The solution was then concentrated under reduced pressure to remove the volatiles, and the crude residue was purified by column chromatography on silica gel (eluent pure petroleum ether) to obtain the desired compounds $\mathbf{S_a}$-$\mathbf{S_p}$ (checked by GC-MS) in 75-95% yields.

(2) A 1,4-dioxane (10.0 mL) and H_2O (1.0 mL) solution containing **S** (1.5 mmol), phenylboronic acids (1.65 mmol), $PdCl_2(PPh_3)_2$ (5.0 mol%, 52.7 mg, 0.075 mmol) and KF (261.0 mg, 4.5 mmol) in a 25 mL screw-capped thick-walled Pyrex tube was stirred under N_2 at 100 °C in an oil bath for 12 h. After the reaction was completed, the reaction mixture was cooled to room temperature (TLC monitoring, eluent petroleum ether/ethyl acetate, 15/1 v/v), and filtrated through a short pad of celite. The solution was then concentrated under reduced pressure to remove the volatiles, and the crude residue was

purified by column chromatography on silica gel (eluent petroleum ether/ethyl acetate, gradient mixture ratio from 30 / 1 to 15 / 1 v/v) to afford product **1a-p** in 24-94% yields.

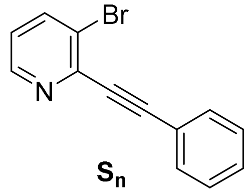

3-Bromo-2-(phenylethynyl)pyridine (**S_n**). Pale yellow oil (478 mg, 1.85 mmol, 93% yield). R_f = 0.70 (pure PE). ^{1}H NMR (400 MHz, $CDCl_3$) δ 8.52 (dd, $^3J_{H,H}$ = 4.8 Hz, $^4J_{H,H}$ = 1.5 Hz, 1H), 7.89 (dd, $^3J_{H,H}$ = 8.2 Hz, $^4J_{H,H}$ = 1.6 Hz, 1H), 7.66 – 7.63 (m, 2H), 7.39 – 7.34 (m, 3H), 7.09 (dd, $^3J_{H,H}$ = 8.2 Hz, $^4J_{H,H}$ = 4.6 Hz, 1H) ppm. ^{13}C NMR (100 MHz, $CDCl_3$) δ 148.2, 143.6, 139.8, 132.1, 129.4, 128.4, 123.8, 123.5, 121.9, 94.0, 87.5 ppm. HRMS (ESI IT-TOF) *m/z* $[M + H]^+$ Calcd for $C_{13}H_9BrN$ 257.9913, found 257.9913.

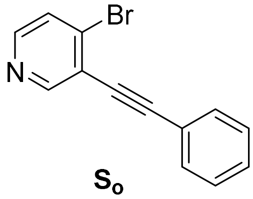

4-Bromo-3-(phenylethynyl)pyridine (**S_o**). Pale yellow oil (488 mg, 1.89 mmol, 95% yiled). R_f = 0.70 (pure PE). ^{1}H NMR (400 MHz, $CDCl_3$) δ 8.70 (s, 1H), 8.29 (d, $^3J_{H,H}$ = 5.2 Hz, 1H), 7.60 – 7.57 (m, 2H), 7.53 (d, $^3J_{H,H}$ = 5.4 Hz, 1H), 7.38 – 7.35 (m, 3H) ppm. ^{13}C NMR (100 MHz, $CDCl_3$) δ 152.9, 148.6, 135.2, 131.8, 129.2, 128.5, 127.2, 123.1, 122.2, 97.0, 84.8 ppm. HRMS (ESI IT-TOF) *m/z* $[M + H]^+$ Calcd for $C_{13}H_9BrN$ 257.9913, found 257.9913.

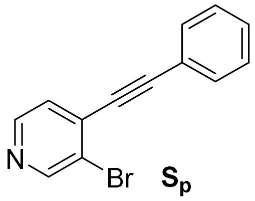

S_p

3-Bromo-4-(phenylethynyl)pyridine (**S_p**). Pale yellow oil (406 mg, 1.57 mmol, 79% yield). R_f = 0.70 (pure PE). ^{1}H NMR (400 MHz, $CDCl_3$) δ 8.77 (s, 1H), 8.47 (d, $^3J_{H,H}$ = 5.0 Hz, 1H), 7.61 – 7.58 (m, 2H), 7.43 – 7.35 (m, 4H) ppm. ^{13}C NMR (100 MHz, $CDCl_3$) δ 151.77, 147.79, 133.07, 132.07, 129.67, 128.58, 126.56, 123.11, 121.82, 98.75, 85.70 ppm. HRMS (ESI IT-TOF) *m/z* $[M + H]^+$ Calcd for $C_{13}H_9BrN$ 257.9913, found 257.9913.

Compounds **1a**, **1c**, **1e**, **1f**, **1g** are known compounds, which were confirmed by their ^{1}H NMR and ^{13}C NMR spectroscopic data [41].

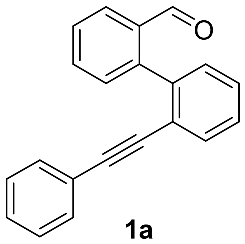

1a

2'-(Phenylethynyl)-(1,1'-biphenyl)-2-carbaldehyde (**1a**). Pale yellow oil (355 mg, 1.26 mmol, 84% yield). Rf = 0.40 (petroleum ether/ethyl acetate, 10/1 v/v). 1H NMR (400 MHz, $CDCl_3$) δ_H 9.94 (s, 1H), 8.09 (dd, $^3J_{H,H}$ = 7.8 Hz, $^4J_{H,H}$ = 1.5 Hz, 1H), 7.68 – 7.64 (m, 2H), 7.54 (t, $^3J_{H,H}$ = 7.6 Hz, 1H), 7.46 – 7.38 (m, 4H), 7.25 – 7.22 (m, 4H), 7.17 – 7.15 (m, 2H) ppm. ^{13}C NMR (100 MHz, $CDCl_3$) δ_C 192.0, 144.4, 140.4, 134.3, 133.6, 132.1, 131.4, 130.4, 128.6, 128.4, 128.3, 127.0, 123.8, 122.8, 93.9, 88.3 ppm.

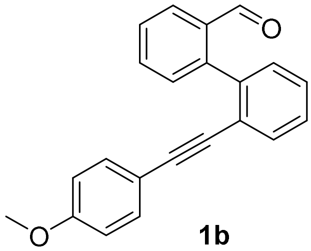

1b

2'-[(4-Methoxyphenyl)ethynyl]-(1,1'-biphenyl)-2-carbaldehyde (**1b**). Yellow oil (332 mg, 1.07 mmol, 71% yield). R_f = 0.55 (petroleum ether/ethyl acetate, 10/1 v/v). ^{1}H NMR (400 MHz, $CDCl_3$) δ_H 9.93 (s, 1H), 8.08 (dd, $^3J_{H,H}$ = 7.9 Hz, $^4J_{H,H}$ = 1.5 Hz, 1H), 7.66-7.59 (m, 2H), 7.52 (dd app. t, $^3J_{H,H}$ = 7.5 Hz, 1H), 7.43-7.36 (m, 4H), 7.12-7.08 (m, 2H), 6.77-6.74 (m, 2H), 3.74 (s, 3H) ppm. ^{13}C NMR (100 MHz, $CDCl_3$) δ_C 191.9, 159.8, 144.5, 140.1, 134.3, 133.5, 132.8, 131.8, 131.4, 130.3, 128.3, 128.2, 128.2, 126.8, 124.1, 114.9, 114.0, 94.0, 87.1, 55.3 ppm. HRMS (ESI IT-TOF) *m/z* $[M + H]^+$ Calcd. for $C_{22}H_{17}O_2$ 313.1223, found 313.1223.

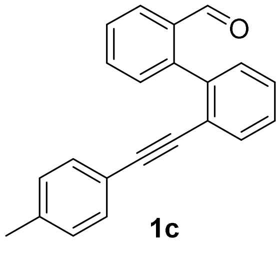

1c

2'-(p-Tolylethynyl)-(1,1'-biphenyl)-2-carbaldehyde (**1c**). Pale yellow oil (417 mg, 1.41 mmol, 94% yield). R_f = 0.50 (petroleum ether/ethyl acetate, 10/1 v/v). ^{1}H NMR (400 MHz, $CDCl_3$) δ_H 9.93 (s, 1H), 8.08 (dd, $^3J_{H,H}$ = 7.7 Hz, $^4J_{H,H}$ = 1.5 Hz, 1H), 7.66 – 7.61 (m, 2H), 7.52 (t, $^3J_{H,H}$ = 7.6 Hz, 1H), 7.44 – 7.36 (m, 4H), 7.07 – 7.02 (m, 4H), 2.29 (s, 3H) ppm. ^{13}C NMR (100 MHz, $CDCl_3$) δ_C 191.9, 144.4, 140.3, 138.7, 134.3, 133.5, 132.0, 131.4, 131.3, 130.3, 129.1, 128.3, 128.3, 126.9, 124.0, 119.7, 94.1, 87.7, 21.6 ppm.

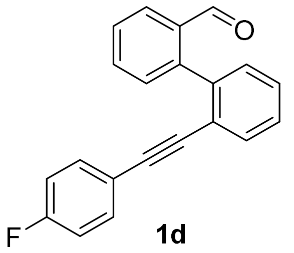

1d

2'-[(4-Fluorophenyl)ethynyl]-(1,1'-biphenyl)-2-carbaldehyde (**1d**). Pale yellow oil (333 mg, 1.11 mmol, 74% yield). R_f = 0.40 (petroleum ether/ethyl acetate, 10/1 v/v). ^{1}H NMR (400 MHz, $CDCl_3$) δ_H 9.93 (s, 1H), 8.08 (dd, $^3J_{H,H}$ = 7.8 Hz, $^4J_{H,H}$ = 1.5 Hz, 1H), 7.67 – 7.61 (m, 2H), 7.53 (t, $^3J_{H,H}$ = 7.6 Hz, 1H), 7.46 – 7.38 (m, 4H), 7.16 – 7.11 (m, 2H), 6.95 – 6.90 (m, 2H) ppm. ^{13}C NMR (100 MHz, $CDCl_3$) δ_C 191.9, 162.7 (d, J = 249.6 Hz), 144.3, 140.3, 134.3, 133.6, 133.3 (d, J = 8.3 Hz), 132.0, 131.4, 130.3, 128.6, 128.3, 126.9, 123.6, 118.8 (d, J = 3.6 Hz), 115.7 (d, J = 22.2 Hz), 92.8, 88.0. HRMS (ESI IT-TOF) m/z [M + H]$^+$ Calcd. for $C_{21}H_{14}FO$ 301.1023, found 301.1023.

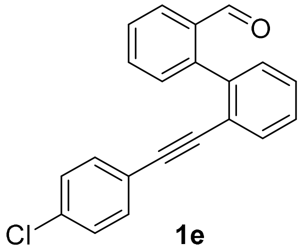

1e

2'-[(4-Chlorophenyl)ethynyl]-(1,1'-biphenyl)-2-carbaldehyde (**1e**). Pale yellow oil (436 mg, 1.38 mmol, 92% yield). R_f = 0.40 (petroleum ether/ethyl acetate, 10/1 v/v). ^{1}H NMR (400 MHz, $CDCl_3$) δ_H 9.92 (s, 1H), 8.08 (dd, $^3J_{H,H}$ = 7.8 Hz, $^4J_{H,H}$ = 1.5 Hz, 1H), 7.69 – 7.62 (m, 2H), 7.55 (t, $^3J_{H,H}$ = 7.5 Hz, 1H), 7.49 – 7.40 (m, 4H), 7.22 – 7.19 (m, 2H), 7.09 – 7.07 (m, 2H) ppm. ^{13}C NMR (100 MHz, $CDCl_3$) δ_C 191.9, 144.3, 140.5,

134.6, 134.3, 133.6, 132.6, 132.1, 131.4, 130.4, 128.8, 128.8, 128.4, 128.4, 127.0, 123.5, 121.3, 92.7, 89.3 ppm.

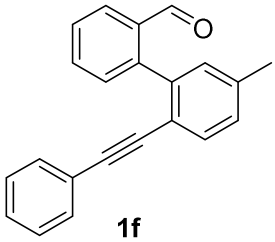

1f

5'-Methyl-2'-(phenylethynyl)-(1,1'-biphenyl)-2-carbaldehyde (**1f**). Pale yellow oil (404 mg, 1.36 mmol, 91% yield). R_f = 0.40 (petroleum ether/ethyl acetate, 10/1 v/v). ^{1}H NMR (400 MHz, $CDCl_3$) δ_H 9.94 (s, 1H), 8.08 (dd, $^3J_{H,H}$ = 7.9 Hz, $^4J_{H,H}$ = 1.6 Hz, 1H), 7.66 – 7.61 (m, 1H), 7.53 – 7.49 (m, 2H), 7.42 (d, $^3J_{H,H}$ = 7.5 Hz, 1H), 7.23 – 7.20 (m, 5H), 7.16 – 7.14 (m, 2H), 2.41 (s, 3H) ppm. ^{13}C NMR (100 MHz, $CDCl_3$) δ_C 192.0, 144.5, 140.3, 138.8, 134.3, 133.5, 131.9, 131.3, 131.3, 131.1, 129.1, 128.3, 128.2, 126.9, 123.0, 120.8, 93.1, 88.5, 21.6 ppm.

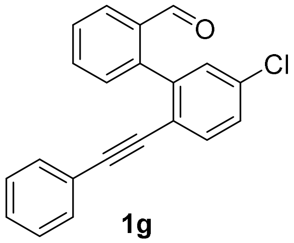

1g

5'-Chloro-2'-(phenylethynyl)-(1,1'-biphenyl)-2-carbaldehyde (**1g**). Pale yellow solid (374 mg, 1.18 mmol, 79% yield). R_f = 0.40 (petroleum ether/ethyl acetate, 10/1 v/v). m.p. 83.7 – 84.2 °C. ^{1}H NMR (400 MHz, $CDCl_3$) δ_H 9.93 (s, 1H), 8.09 (dd, $^3J_{H,H}$ = 7.8 Hz, $^4J_{H,H}$ = 1.5 Hz, 1H), 7.67 (td, $^3J_{H,H}$ = 7.4 Hz, $^4J_{H,H}$ = 1.5 Hz, 1H), 7.58 – 7.54 (m, 2H), 7.42 – 7.39 (m, 3H), 7.26 – 7.21(m, 4H), 7.16 – 7.13 (m, 2H) ppm. ^{13}C NMR (100

MHz, $CDCl_3$) δ_C 191.4, 142.9, 142.1, 134.5, 134.3, 133.8, 133.1, 131.4, 131.2, 130.3, 128.8, 128.6, 128.4, 127.3, 122.5, 94.8, 87.3 ppm.

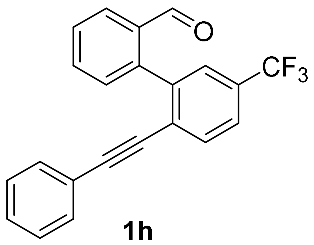

1h

2'-(Phenylethynyl)-5'-(trifluoromethyl)-(1,1'-biphenyl)-2-carbaldehyde (**1h**). Pale yellow solid (483 mg, 1.38 mmol, 92% yield). R_f = 0.40 (petroleum ether/ethyl acetate, 10/1 v/v). m.p. 79.5 – 80.1 °C. ^{1}H NMR (400 MHz, $CDCl_3$) δ_H 9.92 (s, 1H), 8.11 (dd, $^3J_{H,H}$ = 7.8 Hz, $^4J_{H,H}$ = 1.5 Hz, 1H), 7.75 – 7.67 (m, 4H), 7.58 (t, $^3J_{H,H}$ = 7.6 Hz, 1H), 7.41 (d, $^3J_{H,H}$ = 7.8 Hz, 1H), 7.28 – 7.26 (m, 3H), 7.18 – 7.15 (m, 2H) ppm. ^{13}C NMR (100 MHz, $CDCl_3$) δ_C 191.1, 142.7, 141.2, 134.3, 133.9, 132.4, 131.6, 131.3, 130.3 (q, J = 32.7 Hz), 129.1, 129.0, 128.5, 127.6, 127.5, 126.9 (q, J = 3.8 Hz), 125.1 (q, J = 3.5 Hz), 123.9 (q, J = 273.7 Hz), 122.1, 96.4, 87.1 ppm. ^{19}F NMR (376 MHz, Chloroform-*d*) δ -62.53. HRMS (ESI IT-TOF) *m/z* $[M + H]^+$ Calcd. for $C_{22}H_{14}F_3O$ 351.0991, found 351.0991.

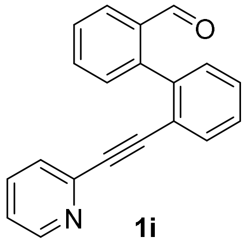

1i

2'-(Pyridin-2-ylethynyl)-(1,1'-biphenyl)-2-carbaldehyde (**1i**). Pale yellow solid (378 mg, 1.34 mmol, 89% yield). R_f = 0.40 (petroleum ether/ethyl acetate, 10/1 v/v). m.p.

104.6 – 104.9 °C. ^{1}H NMR (400 MHz, $CDCl_3$) δ_H 9.95 (s, 1H), 8.50 (d, $^3J_{H,H}$ = 3.3 Hz, 1H), 8.09 (d, $^3J_{H,H}$ = 7.7 Hz, 1H), 7.74 (dd, $^3J_{H,H}$ = 7.3 Hz, $^4J_{H,H}$ = 1.7 Hz, 1H), 7.66 (td, $^3J_{H,H}$ = 7.5 Hz, $^4J_{H,H}$ = 1.4 Hz, 1H), 7.55 – 7.39 (m, 6H), 7.15 – 7.11 (m, 1H), 7.00 (d, $^3J_{H,H}$ = 7.8 Hz, 1H) ppm. ^{13}C NMR (100 MHz, $CDCl_3$) δ_C 191.6, 149.9, 143.9, 142.8, 140.6, 136.0, 134.1, 133.5, 132.6, 131.3, 130.3, 129.1, 128.3, 128.3, 127.1, 126.8, 122.8, 122.6, 92.7, 87.8 ppm. HRMS (ESI IT-TOF) *m/z* $[M + H]^+$ Calcd. for $C_{20}H_{14}NO$ 284.1070, found 284.1069.

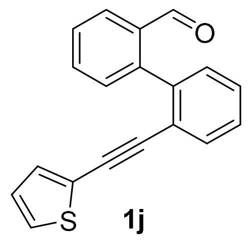

1j

2'-(Thiophen-2-ylethynyl)-(1,1'-biphenyl)-2-carbaldehyde (**1j**). Pale yellow oil (363 mg, 1.26 mmol, 84% yield). R_f = 0.40 (petroleum ether/ethyl acetate, 10/1 v/v). ^{1}H NMR (400 MHz, $CDCl_3$) δ_H 9.91 (s, 1H), 8.08 (dd, $^3J_{H,H}$ = 7.7 Hz, $^4J_{H,H}$ = 1.5 Hz, 1H), 7.67 – 7.59 (m, 2H), 7.52 (t, $^3J_{H,H}$ = 7.6 Hz, 1H), 7.45 – 7.36 (m, 4H), 7.18 (dd, $^3J_{H,H}$ = 5.1 Hz, $^4J_{H,H}$ = 1.2 Hz, 1H), 6.98 (dd, $^3J_{H,H}$ = 3.7 Hz, $^4J_{H,H}$ = 1.2 Hz, 1H), 6.88 (dd, $^3J_{H,H}$ = 5.2 Hz, $^3J_{H,H}$ = 3.6 Hz, 1H) ppm. ^{13}C NMR (100 MHz, $CDCl_3$) δ_C 191.7, 144.1, 140.1, 134.2, 133.5, 132.0, 131.6, 131.3, 130.3, 128.5, 128.3, 128.2, 127.7, 127.1, 127.0, 123.4, 122.6, 92.0, 87.3 ppm. HRMS (ESI IT-TOF) *m/z* $[M + H]^+$ Calcd. for $C_{19}H_{13}OS$ 289.0682, found 289.0682.

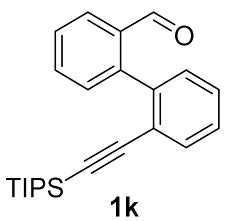

1k

2'-[(Triisopropylsilyl)ethynyl]-(1,1'-biphenyl)-2-carbaldehyde (**1k**). White solid (391 mg, 1.08 mmol, 72% yield). R_f = 0.50 (petroleum ether/ethyl acetate, 10/1 v/v). m.p. 68.0 – 68.3 °C. ^{1}H NMR (400 MHz, $CDCl_3$) δ_H 9.85 (s, 1H), 8.01 (d, $^3J_{H,H}$ = 7.7 Hz, 1H), 7.61 – 7.58 (m, 2H), 7.46 (t, $^3J_{H,H}$ = 7.6 Hz, 1H), 7.41 – 7.34 (m, 3H), 7.31 – 7.28 (m, 1H), 0.91 (s, 21H) ppm. ^{13}C NMR (100 MHz, $CDCl_3$) δ_C 191.6, 144.6, 140.7, 134.1, 133.4, 132.7, 131.0, 130.1, 128.3, 128.1, 128.0, 127.0, 123.9, 105.2, 95.7, 18.5, 11.1. HRMS (ESI IT-TOF) *m/z* $[M + H]^+$ Calcd. for $C_{24}H_{31}OSi$ 363.2139, found 363.2138.

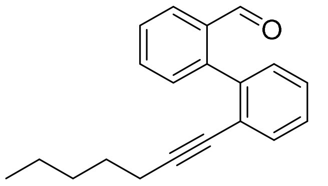

1l

2'-(Hept-1-yn-1-yl)-(1,1'-biphenyl)-2-carbaldehyde (**1l**). Pale yellow oil (99 mg, 0.36 mmol, 24% yield). R_f = 0.50 (petroleum ether/ethyl acetate, 10/1 v/v). ^{1}H NMR (400 MHz, $CDCl_3$) δ_H 9.85 (s, 1H), 8.03 (d, $^3J_{H,H}$ = 7.7 Hz, 1H), 7.64 – 7.60 (m, 1H), 7.50 – 7.49 (m, 2H), 7.35 – 7.32 (m, 4H), 7.25 (s, 1H), 2.17 – 2.13 (s, 2H), 1.33 – 1.26 (m, 2H), 1.21 – 1.16 (m, 2H), 1.11 – 1.05 (m, 2H), 0.83 – 0.79 (m, 3H) ppm. ^{13}C NMR (100 MHz, $CDCl_3$) δ_C 192.0, 144.7, 140.2, 134.1, 133.4, 132.0, 131.2, 130.1, 128.1, 128.0, 127.7, 126.7, 124.5, 95.5, 79.5, 30.8, 27.8, 22.2, 19.3, 14.0 ppm. HRMS (ESI IT-TOF) *m/z* $[M + H]^+$ Calcd. for $C_{20}H_{21}O$ 277.1587, found 277.1587.

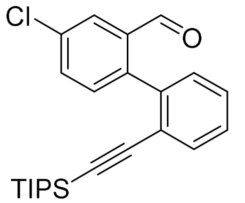

1m

4-Chloro-2'-[(triisopropylsilyl)ethynyl]-(1,1'-biphenyl)-2-carbaldehyde (**1m**). Pale yellow oil (422 mg, 1.07 mmol, 71% yield). R_f = 0.50 (petroleum ether/ethyl acetate, 10/1 v/v). ^{1}H NMR (400 MHz, $CDCl_3$) δ_H 9.77 (s, 1H), 7.98 (d, $^3J_{H,H}$ = 2.6 Hz, 1H), 7.62 – 7.56 (m, 2H), 7.44 – 7.37 (m, 2H), 7.34 (d, $^3J_{H,H}$ = 8.2 Hz, 1H), 7.30 – 7.28 (m, 1H), 0.92 (s, 21H) ppm. ^{13}C NMR (100 MHz, $CDCl_3$) δ_C 190.4, 142.8, 139.5, 135.3, 134.7, 133.3, 132.9, 132.6, 130.0, 128.5, 128.4, 126.9, 124.0, 104.9, 96.4, 18.5, 11.1 ppm. HRMS (ESI IT-TOF) *m*/*z* $[M + H]^+$ Calcd. for $C_{21}H_{24}ClOSi$ 355.1279, found 355.1278.

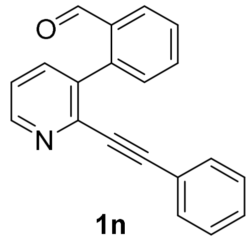

1n

2-[2-(Phenylethynyl)pyridin-3-yl]benzaldehyde (**1n**). Pale yellow oil (365 mg, 1.29 mmol, 86% yield). R_f = 0.40 (petroleum ether/ethyl acetate, 10/1 v/v). ^{1}H NMR (400 MHz, $CDCl_3$) δ_H 9.96 (s, 1H), 8.69 (dd, $^3J_{H,H}$ = 4.7, $^4J_{H,H}$ = 1.8 Hz, 1H), 8.11 (dd, $^3J_{H,H}$ = 7.8 Hz, $^4J_{H,H}$ = 1.4 Hz, 1H), 7.73 – 7.67 (m, 2H), 7.59 (t, $^3J_{H,H}$ = 7.5 Hz, 1H), 7.43 – 7.36 (m, 2H), 7.30 – 7.19 (m, 5H) ppm. ^{13}C NMR (100 MHz, $CDCl_3$) δ_C 190.8, 149.7, 142.8, 141.4, 137.5, 136.6, 134.2, 133.7, 131.7, 131.3, 129.1, 128.9, 128.3, 127.7, 122.6,

121.6, 93.7, 87.7 ppm. HRMS (ESI IT-TOF) *m/z* $[M + H]^+$ Calcd. for $C_{20}H_{14}NO$ 284.1070, found 284.1069.

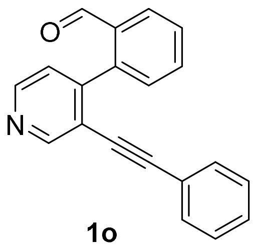

1o

2-[3-(Phenylethynyl)pyridin-4-yl]benzaldehyde (**1o**). Pale yellow oil (386 mg, 1.36 mmol, 91% yield). R_f = 0.40 (petroleum ether/ethyl acetate, 10/1 v/v). ^{1}H NMR (400 MHz, $CDCl_3$) δ_H 9.93 (s, 1H), 8.86 (s, 1H), 8.65 (d, $^3J_{H,H}$ = 5.1 Hz, 1H), 8.11 (dd, $^3J_{H,H}$ = 7.9, 1.5 Hz, 1H), 7.71 (td, $^3J_{H,H}$ = 7.5 Hz, $^4J_{H,H}$ = 1.5 Hz, 1H), 7.61 (t, $^3J_{H,H}$ = 7.7 Hz, 1H), 7.43 – 7.40 (m, 1H), 7.34 (d, $^3J_{H,H}$ = 5.1 Hz, 1H), 7.31 – 7.20 (m, 6H) ppm. ^{13}C NMR (100 MHz, $CDCl_3$) δ_C 190.6, 152.5, 148.7, 147.8, 141.1, 133.8, 131.4, 130.7, 129.3, 129.0, 128.4, 127.8, 124.2, 122.0, 120.6, 96.7, 84.9 ppm. HRMS (ESI IT-TOF) *m/z* $[M + H]^+$ Calcd. for $C_{20}H_{14}NO$ 284.1070, found 284.1069.

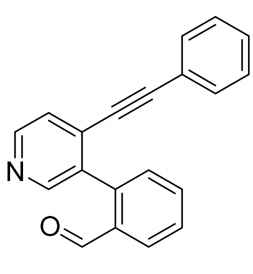

1p

2-[4-(Phenylethynyl)pyridin-3-yl]benzaldehyde (**1p**). Pale yellow oil (378 mg, 1.33 mmol, 89% yield). R_f = 0.40 (petroleum ether/ethyl acetate, 10/1 v/v). ^{1}H NMR (400 MHz, $CDCl_3$) δ_H 9.95 (s, 1H), 8.67 – 8.66 (m, 2H), 8.12 (d, $^3J_{H,H}$ = 7.8 Hz, 1H), 7.71

(td, $^{3}J_{H,H}$ = 7.4, $^{4}J_{H,H}$ = 1.4 Hz, 1H), 7.60 (t, $^{3}J_{H,H}$ = 7.6 Hz, 1H), 7.49 (d, $^{3}J_{H,H}$ = 5.1 Hz, 1H), 7.44 (d, $^{3}J_{H,H}$ = 7.6 Hz, 1H), 7.32 – 7.19 (m, 5H) ppm. ^{13}C NMR (100 MHz, $CDCl_3$) δ_C 190.8, 150.2, 149.3, 140.1, 135.0, 134.4, 133.8, 131.6, 131.6, 131.4, 129.4, 129.0, 128.4, 127.7, 125.1, 121.5, 98.2, 85.7 ppm. HRMS (ESI IT-TOF) *m*/*z* $[M + H]^+$ Calcd. for $C_{20}H_{14}NO$ 284.1070, found 284.1069.

B-2. Preparation of 2'-alkynyl-biaryl-2-aldehyde *N*-tosylhydrazone 1a'

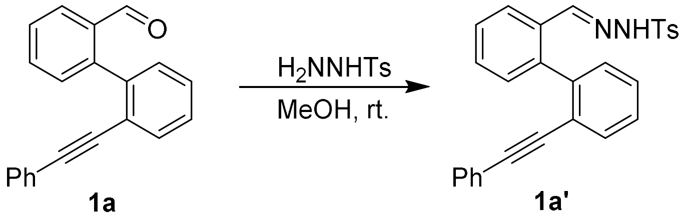

The Synthesis of 2'-alkynyl-biaryl-2-aldehyde *N*-tosylhydrazone 1a':

The mixture of H_2NNHTs (1.1 equiv., 1.10 mmol, 205 mg), 2'-(phenylethynyl)-[1,1'-biphenyl]-2-carbaldehyde (**1a**, 1.00 mmol, 282 mg) and methanol (5.0 mL) in a 25 mL screw-capped thick-walled Pyrex tube was stirred at room temperature for 2 h. After the reaction was completed (checked by TLC), the crude residue was purified by column chromatography on silica gel, eluting with petroleum ether / ethyl acetate (gradient mixture ratio from 5 / 1 to 3 / 1) as eluent to afford product **1a'** in 90% yields.

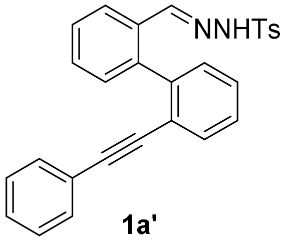

1a'

(E)-4-Methyl-N'-{[2'-(phenylethynyl)-(1,1'-biphenyl)-2-yl]methylene}benzenesulfono-hydrazide (**1a'**). Yellow solid (405 mg, 0.90 mmol, 90% yield). R_f = 0.45 (petroleum ether/ethyl acetate, 2/1 v/v). m.p. 77.3 – 77.8 °C. ^{1}H NMR (400 MHz, $CDCl_3$) δ 8.06 – 8.03 (m, 1H), 7.76 (d, $^3J_{H,H}$ = 8.5 Hz, 3H), 7.59 (s, 1H), 7.56 (dd, $^3J_{H,H}$ = 5.7 Hz, $^4J_{H,H}$ = 3.4 Hz, 1H), 7.43 – 7.38 (m, 2H), 7.34 (dd, $^3J_{H,H}$ = 5.7 Hz, $^4J_{H,H}$ = 3.3 Hz, 2H), 7.31 – 7.29 (m, 1H), 7.22 – 7.16 (m, 6H), 7.08 (d, $^3J_{H,H}$ = 7.2 Hz, 2H), 2.33 (s, 3H) ppm. ^{13}C NMR (100 MHz, $CDCl_3$) δ 146.5, 144.0, 141.6, 141.1, 135.5, 132.0, 131.4, 130.7, 130.3,

129.8, 129.6, 128.3, 128.3, 128.2, 128.0, 127.9, 127.8, 125.6, 123.2, 122.8, 93.6, 88.2, 21.6 ppm. HRMS (ESI IT-TOF) *m*/*z* $[M + H]^+$ Calcd for $C_{21}H_{15}N_2$ 295.1230, found 295.1229.

B-3. Preparation of 3-substituted 1*H*-dibenzo[*e*,*g*]indazoles 2a-p

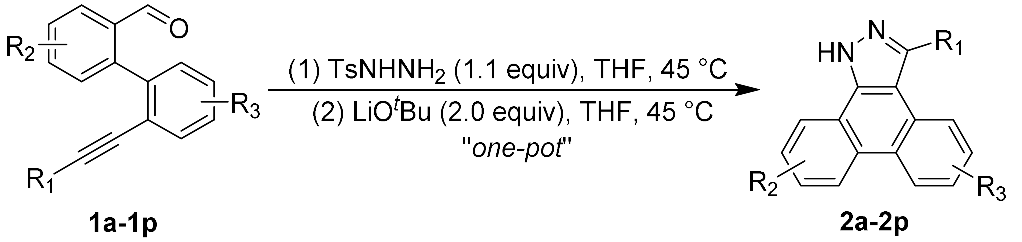

General procedure for the preparation of 1*H*-dibenzo[*e*,*g*]indazoles 2a-p:

A THF (5.0 mL) solution containing 2'-alkynyl-biaryl-2-aldehydes (**1**, 282.0 mg, 1.0 mmol) and *p*-toluenesulfonhydrazide (204.9 mg, 1.1 mmol) in a 25 mL screw-capped thick-walled Pyrex tube was stirred at 45 °C for 1 h. After the reaction was completed (TLC monitoring, eluent petroleum ether/ethyl acetate, 1/1 v/v), LiO^{t}Bu (120.0 mg, 1.5 mmol) and additional THF (2.5 mL) were added and then the mixture was stirred at 45 oC for 1 h. After the reaction was completed (TLC monitoring, eluent petroleum ether/ethyl acetate, 1/1 v/v), the crude residue was directly purified by column chromatography on silica gel (eluent petroleum ether/ethyl acetate, gradient mixture ratio from 5/1 to 2/1 v/v) to afford products **2a-p** in 61-93% yields.

2a was known compounds[38].

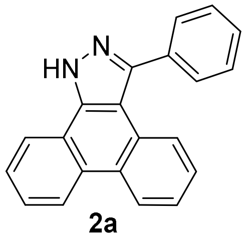

2a

3-Phenyl-1H-dibenzo[e,g]indazole (**2a**). White solid (259 mg, 0.88 mmol, 88% yield). R_f = 0.40 (petroleum ether/ethyl acetate, 1/1 v/v). m.p. 260.4 – 260.8 °C. ^{1}H NMR (400 MHz, DMSO-d_6) δ_H 14.24 – 13.95 (s, 1H), 8.78 – 8.71 (m, 2H), 8.55 (d, $^3J_{H,H}$ = 7.8 Hz,

1H), 8.02 (d, $^3J_{H,H}$ = 8.1 Hz, 1H), 7.74 – 7.69 (m, 4H), 7.59 – 7.54 (m, 3H), 7.50 – 7.40 (m, 2H) ppm. ^{13}C NMR (100 MHz, DMSO-d_6) δ_C 147.3, 137.2, 135.4, 129.6, 128.6, 128.3, 127.5, 127.4, 127.1, 124.9, 124.1, 124.0, 122.6, 122.3, 121.0, 112.5 ppm.

To demonstrate the practical utility of the method as a synthetic tool, a gram-scale reaction of **1a** (5.0 mmol) in 38.0 mL of THF under standard conditions was conducted, and the desired **2a** was obtained in 85% yield (1.25 g).

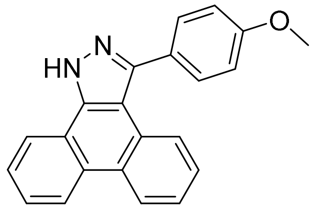

2b

3-(4-Methoxyphenyl)-1H-dibenzo[e,g]indazole (**2b**). White solid (285 mg, 0.88 mmol, 88% yield). R_f = 0.40 (petroleum ether/ethyl acetate, 1/1 v/v). m.p. 204.2 – 204.7 °C. ^{1}H NMR (400 MHz, DMSO-d_6) δ_H 13.97 (s, 1H), 8.71 (dd, $^2J_{H,H}$ = 16.9 Hz, $^3J_{H,H}$ = 8.1 Hz, 2H), 8.56 (d, $^3J_{H,H}$ = 7.7 Hz, 1H), 8.05 (d, $^3J_{H,H}$ = 7.3 Hz, 1H), 7.74 – 7.63 (m, 4H), 7.49 – 7.40 (m, 2H), 7.14 (d, $^3J_{H,H}$ = 8.4 Hz, 2H), 3.84 (s, 3H) ppm. ^{13}C NMR (100 MHz, DMSO-d_6) δ_C 159.3, 147.2, 137.2, 130.9, 129.6, 127.4, 127.3, 127.1, 124.8, 124.0, 123.9, 122.6, 122.4, 121.1, 114.0, 112.6, 55.1 ppm. HRMS (ESI IT-TOF) *m/z* [M + H]$^+$ Calcd. for $C_{22}H_{17}N_2O$ 325.1335, found 325.1334.

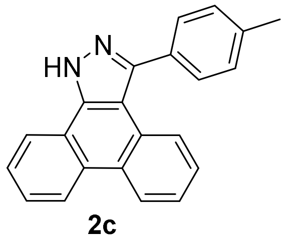

2c

3-(p-Tolyl)-1H-dibenzo[e,g]indazole (**2c**). White solid (262 mg, 0.85 mmol, 85% yield). R_f = 0.40 (petroleum ether/ethyl acetate, 1/1 v/v). m.p. 199.6 – 200.0 °C. ^{1}H NMR (400 MHz, DMSO-d_6) δ_H 11.79 (s, 1H), 8.71 (dd, $^2J_{H,H}$ = 17.1 Hz, $^3J_{H,H}$ = 8.1 Hz, 2H), 8.57 (d, $^3J_{H,H}$ = 7.7 Hz, 1H), 8.06 (d, $^3J_{H,H}$ = 7.7 Hz, 1H), 7.73 (t, $^3J_{H,H}$ = 7.5 Hz, 1H), 7.66 (t, $^3J_{H,H}$ = 7.5 Hz, 1H), 7.61 (d, $^3J_{H,H}$ = 7.8 Hz, 2H), 7.48 – 7.36 (m, 4H), 2.40 (s, 3H) ppm. ^{13}C NMR (100 MHz, DMSO-d_6) δ_C 145.1, 139.0, 137.8, 131.4, 129.7, 129.4, 129.2, 127.5, 127.4, 127.3, 127.1, 125.6, 124.9, 124.1, 123.9, 122.6, 122.3, 112.3, 20.9 ppm. HRMS (ESI IT-TOF) *m/z* $[M + H]^+$ Calcd. for $C_{22}H_{17}N_2$ 309.1386, found 309.1385.

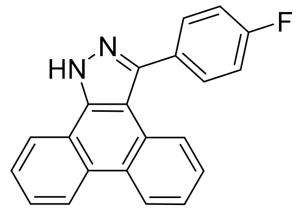

2d

3-(4-Fluorophenyl)-1H-dibenzo[e,g]indazole (**2d**). White solid (262 mg, 0.84 mmol, 84% yield). R_f = 0.40 (petroleum ether/ethyl acetate, 1/1 v/v). m.p. 235.7 – 236.2 °C. ^{1}H NMR (400 MHz, DMSO-d_6) δ_H 14.31 – 14.03 (s, 1H), 8.70 – 8.58 (m, 3H), 8.00 (s, 1H), 7.80 – 7.62 (m, 4H), 7.43 – 7.39 (m, 4H) ppm. ^{13}C NMR (100 MHz, DMSO-d_6) δ_C 162.2 (d, J = 245.1 Hz), 146.4, 137.3, 131.8, 131.7, 131.6, 129.6, 127.4, 127.3, 127.1, 124.9, 124.1, 124.0, 122.6, 122.4, 121.0, 115.5 (d, J = 21.5 Hz), 112.6 ppm. HRMS (ESI IT-TOF) *m/z* $[M + H]^+$ Calcd. for $C_{21}H_{14}FN_2$ 313.1136, found 313.1134.

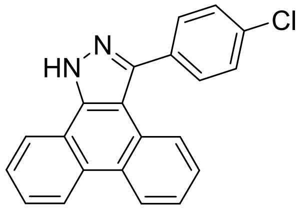

2e

3-(4-Chlorophenyl)-1H-dibenzo[e,g]indazole (**2e**). White solid (262 mg, 0.80 mmol, 80% yield). R_f = 0.40 (petroleum ether/ethyl acetate, 1/1 v/v). m.p. 265.3 – 265.9 °C. ^{1}H NMR (400 MHz, DMSO-d_6) δ_H 14.18 (s, 1H), 8.76 (dd, $^2J_{H,H}$ = 17.0 Hz, $^3J_{H,H}$ = 8.0 Hz, 2H), 8.53 (dd, $^3J_{H,H}$ = 7.7 Hz, $^4J_{H,H}$ = 1.7 Hz, 1H), 7.96 (d, $^3J_{H,H}$ = 7.5 Hz, 1H), 7.76 – 7.68 (m, 4H), 7.66 – 7.64 (m, 2H), 7.53 – 7.44 (m, 2H) ppm. ^{13}C NMR (100 MHz, DMSO-d_6) δ_C 144.8, 138.5, 133.6, 133.3, 131.3, 129.6, 128.7, 127.6, 127.4, 127.4, 127.1, 127.1, 125.0, 124.1, 123.9, 122.6, 122.4, 121.7, 112.5 ppm. HRMS (ESI IT-TOF) *m/z* [M + H]$^+$ Calcd. for $C_{21}H_{14}ClN_2$ 329.0840, found 329.0838.

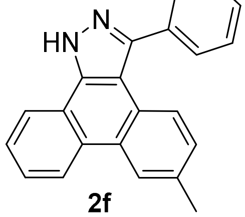

2f

6-Methyl-3-phenyl-1H-dibenzo[e,g]indazole (**2f**). White solid (265 mg, 0.86 mmol, 86% yield). R_f = 0.40 (petroleum ether/ethyl acetate, 1/1 v/v). m.p. 235.6 – 235.9 °C. ^{1}H NMR (400 MHz, DMSO-d_6) δ_H 14.22 – 13.93 (s, 1H), 8.76 – 8.52 (m, 3H), 7.94 (d, $^3J_{H,H}$ = 8.3 Hz, 1H), 7.76 – 7.52 (m, 7H), 7.19 (d, $^3J_{H,H}$ = 8.4 Hz, 1H), 2.44 (s, 3H) ppm. ^{13}C NMR (100 MHz, DMSO-d_6) δ_C 147.1, 137.0, 135.6, 134.0, 129.6, 129.5, 128.5, 128.4, 128.2, 127.5, 127.3, 127.1, 124.8, 123.9, 122.5, 122.3, 121.1, 112.6, 21.2 ppm. HRMS (ESI IT-TOF) *m/z* [M + H]$^+$ Calcd. for $C_{22}H_{17}N_2$ 309.1386, found 309.1385.

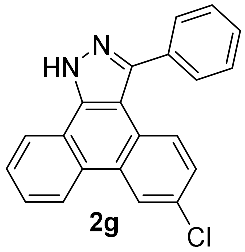

2g

6-Chloro-3-phenyl-1H-dibenzo[e,g]indazole (**2g**). White solid (276 mg, 0.84 mmol, 84% yield). R_f = 0.40 (petroleum ether/ethyl acetate, 1/1 v/v). m.p. 286.9 – 287.3 °C. ^{1}H NMR (400 MHz, DMSO-d_6) δ_H 13.77 (s, 1H), 8.73 – 8.69 (m, 2H), 8.51 (d, $^3J_{H,H}$ = 7.6 Hz, 1H), 7.92 (d, $^3J_{H,H}$ = 8.6 Hz, 1H), 7.75 – 7.64 (m, 4H), 7.60 – 7.52 (m, 3H), 7.40 (dd, $^3J_{H,H}$ = 8.6 Hz, $^4J_{H,H}$ = 2.1 Hz, 1H) ppm. ^{13}C NMR (100 MHz, DMSO-d_6) δ_C 129.8, 129.5, 129.2, 128.7, 128.5, 128.1, 127.4, 127.0, 125.8, 124.3, 124.2, 123.6, 122.3, 111.7. HRMS (ESI IT-TOF) *m/z* $[M + H]^+$ Calcd. for $C_{21}H_{14}ClN_2$ 329.0840, found 329.0838.

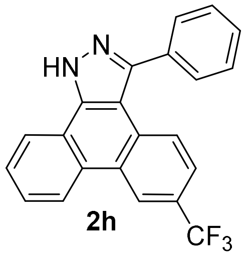

2h

3-Phenyl-6-(trifluoromethyl)-1H-dibenzo[e,g]indazole (**2h**). White solid (300 mg, 0.83 mmol, 83% yield). R_f = 0.40 (petroleum ether/ethyl acetate, 1/1 v/v). m.p. 276.9 – 277.3 °C. ^{1}H NMR (400 MHz, DMSO-d_6) δ_H 14.29 – 14.10 (s, 1H), 8.88 – 8.84 (m, 1H), 8.72 – 8.65 (m, 1H), 8.55 – 8.47 (m, 1H), 8.07 – 8.02 (m, 1H), 7.71 – 7.51 (m, 8H) ppm. ^{13}C NMR (100 MHz, DMSO-d_6) δ_C 147.7, 138.0, 135.0, 129.8, 129.5, 128.8, 128.6, 128.4, 128.1, 127.6, 127.0, 126.0, 125.0 (q, J = 31.7 Hz), 124.1, 123.3 (d, J = 6.7 Hz), 122.8,

122.3, 121.1 (d, J = 15.7 Hz), 111.7 ppm. ^{19}F NMR (376 MHz, DMSO-d_6) δ -60.08. HRMS (ESI IT-TOF) m/z [M + H]$^+$ Calcd. for $C_{22}H_{14}F_3N_2$ 363.1104, found 363.1102.

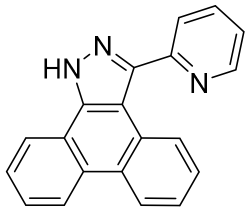

2i

3-(Pyridin-2-yl)-1H-dibenzo[e,g]indazole (**2i**). White solid (230 mg, 0.78 mmol, 78% yield). R_f = 0.40 (petroleum ether/ethyl acetate, 1/1 v/v). m.p. 209.3 – 209.7 °C. ^{1}H NMR (400 MHz, DMSO-d_6) δ_H 14.44 – 14.28 (s, 1H), 9.04 – 9.02 (m, 1H), 8.86 (d, $^3J_{H,H}$ = 4.9 Hz, 1H), 8.79 – 8.58 (m, 3H), 8.06 – 7.97 (m, 2H), 7.78 – 7.67 (m, 2H), 7.52 – 7.49 (m, 3H) ppm. ^{13}C NMR (100 MHz, DMSO-d_6) δ_C 154.3, 148.8, 147.1, 137.8, 137.0, 129.7, 127.5, 127.5, 127.4, 127.3, 127.0, 125.6, 125.2, 124.3, 124.0, 123.7, 123.1, 122.3, 120.9, 113.5. HRMS (ESI IT-TOF) m/z [M + H]$^+$ Calcd. for $C_{20}H_{14}N_3$ 296.1182, found 296.1181.

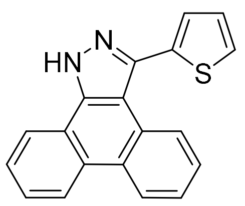

2j

3-(Thiophen-2-yl)-1H-dibenzo[e,g]indazole (**2j**). White solid (273 mg, 0.91 mmol, 91% yield). R_f = 0.40 (petroleum ether/ethyl acetate, 1/1 v/v). m.p. 255.4 – 255.9 °C. ^{1}H NMR (400 MHz, DMSO-d_6) δ_H 14.40 – 14.17 (s, 1H), 8.71 – 8.56 (m, 3H), 8.34 – 8.31 (m, 1H), 7.76 – 7.73 (m, 2H), 7.66 (t, $^3J_{H,H}$ = 7.7 Hz, 1H), 7.56 (d, $^3J_{H,H}$ = 3.6 Hz, 1H),

7.52 – 7.46 (m, 2H), 7.32 – 7.30 (m, 1H) ppm. ^{13}C NMR (100 MHz, DMSO-d_6) δ_C 140.4, 137.4, 136.1, 129.6, 128.0, 127.7, 127.5, 127.3, 127.1, 126.9, 125.1, 124.1, 124.0, 122.6, 122.3, 120.8, 113.2. HRMS (ESI IT-TOF) *m/z* $[M + H]^+$ Calcd. for $C_{19}H_{13}N_2S$ 301.0794, found 301.0793.

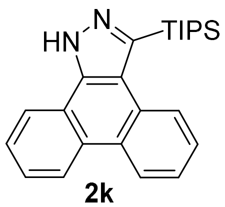

2k

3-(Triisopropylsilyl)-1H-dibenzo[e,g]indazole (**2k**). White solid (348 mg, 0.93 mmol, 93% yield). R_f = 0.40 (petroleum ether/ethyl acetate, 1/1 v/v). m.p. 87.8 – 88.3 °C. ^{1}H NMR (400 MHz, $CDCl_3$) δ_H 12.12 (s, 1H), 8.75 (d, $^3J_{H,H}$ = 7.6 Hz, 1H), 8.58 – 8.53 (m, 2H), 8.27 (d, $^3J_{H,H}$ = 7.8 Hz, 1H), 7.64 – 7.46 (m, 4H), 1.78 (hept, $^3J_{H,H}$ = 7.5 Hz, 3H), 1.12 (d, $^3J_{H,H}$ = 7.7 Hz, 18H) ppm. ^{13}C NMR (100 MHz, $CDCl_3$) δ_C 130.5, 129.1, 128.7, 127.3, 127.2, 126.5, 126.0, 125.3, 124.0, 123.6, 123.4, 123.2, 18.8, 12.7 ppm. HRMS (ESI IT-TOF) *m/z* $[M + H]^+$ Calcd. for $C_{24}H_{31}N_2Si$ 375.2251, found 375.2251.

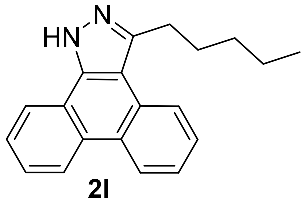

2l

3-Pentyl-1H-dibenzo[e,g]indazole (**2l**). White solid (176 mg, 0.61 mmol, 61% yield). R_f = 0.40 (petroleum ether/ethyl acetate, 1/1 v/v). m.p. 188.5 – 188.9 °C. ^{1}H NMR (400 MHz, DMSO-d_6) δ_H 13.69 – 13.49 (s, 1H), 8.78 – 8.67 (m, 2H), 8.44 (d, $^3J_{H,H}$ = 7.5 Hz,

1H), 8.24 – 8.15 (m, 1H), 7.69 – 7.52 (m, 4H), 3.19 (t, $^3J_{H,H}$ = 7.6 Hz, 2H), 1.82 – 1.80 (m, 2H), 1.42 – 1.32 (m, 4H), 0.87 (t, $^3J_{H,H}$ = 7.1 Hz, 3H) ppm. ^{13}C NMR (100 MHz, DMSO-d_6) δ_C 147.3, 137.1, 129.4, 127.6, 127.3, 127.1, 124.4, 124.0, 123.1, 122.2, 121.1, 112.4, 31.2, 28.9, 27.7, 21.9, 13.9 ppm. HRMS (ESI IT-TOF) *m/z* [M + H]$^+$ Calcd. for $C_{20}H_{21}N_2$ 289.1699, found 289.1698.

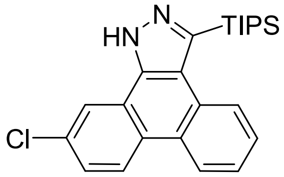

2m

10-Chloro-3-(triisopropylsilyl)-1H-dibenzo[e,g]indazole (**2m**). White solid (335 mg, 0.82 mmol, 82% yield). R_f = 0.40 (petroleum ether/ethyl acetate, 1/1 v/v). m.p. 191.1 – 191.7 °C. ^{1}H NMR (400 MHz, CDCl$_3$) δ_H 11.96 (s, 1H), 8.68 (s, 1H), 8.52 (d, $^3J_{H,H}$ = 7.9 Hz, 1H), 8.47 (d, $^3J_{H,H}$ = 9.0 Hz, 1H), 8.23 (d, $^3J_{H,H}$ = 7.7 Hz, 1H), 7.58 – 7.50 (m, 3H), 1.76 (hept, $^3J_{H,H}$ = 7.5 Hz, 3H), 1.15 (d, $^3J_{H,H}$ = 7.6 Hz, 18H) ppm. ^{13}C NMR (100 MHz, DMSO-d_6) δ_C 133.3, 129.0, 128.7, 128.5, 127.6, 126.9, 125.7, 125.5, 125.1, 124.0, 123.9, 122.8, 18.9, 12.7 ppm. HRMS (ESI IT-TOF) *m/z* [M + H]$^+$ Calcd. for $C_{24}H_{30}ClN_2Si$ 409.1861, found 409.1860.

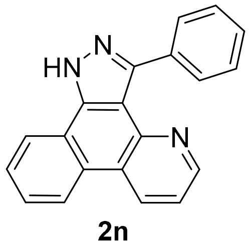

2n

3-Phenyl-1H-benzo[f]pyrazolo[3,4-h]quinoline (**2n**). White solid (260 mg, 0.88 mmol, 88% yield). R_f = 0.40 (petroleum ether/ethyl acetate, 1/1 v/v). m.p. 265.7 – 266.2 °C. ^{1}H NMR (400 MHz, DMSO-d_6) δ_H 14.39 – 14.16 (s, 1H), 9.02 – 8.92 (m, 1H), 8.78 – 8.56 (m, 3H), 8.29 – 8.14 (m, 2H), 7.79 – 7.62 (m, 2H), 7.55 – 7.42 (m, 4H) ppm. ^{13}C NMR (100 MHz, DMSO-d_6) δ_C 148.6, 148.2, 147.4, 145.9, 144.6, 139.9, 139.7, 134.6, 131.7, 130.1, 129.9, 129.6, 128.9, 128.5, 128.0, 127.9, 127.6, 127.4, 126.0, 124.1, 123.8, 123.4, 122.4, 122.3, 120.8, 120.5, 120.1, 113.4, 112.5 ppm. HRMS (ESI IT-TOF) *m*/*z* $[M + H]^+$ Calcd. for $C_{20}H_{14}N_3$ 296.1182, found 296.1181.

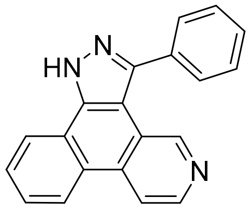

2o

3-Phenyl-1H-benzo[f]pyrazolo[3,4-h]isoquinoline (**2o**). White solid (266 mg, 0.90 mmol, 90% yield). R_f = 0.40 (petroleum ether/ethyl acetate, 1/1 v/v). m.p. 277.9 – 278.4 °C. ^{1}H NMR (400 MHz, DMSO-d_6) δ_H 14.37 – 14.12 (s, 1H), 9.22 (s, 1H), 8.82 – 8.72 (m, 1H), 8.56 – 8.52 (m, 3H), 7.85 – 7.59 (m, 7H) ppm. ^{13}C NMR (100 MHz, DMSO-d_6) δ_C 146.9, 145.1, 144.1, 137.7, 135.1, 132.5, 129.6, 129.4, 128.7, 128.5, 127.7, 127.5, 124.7, 122.4, 117.5, 110.6. HRMS (ESI IT-TOF) *m*/*z* $[M + H]^+$ Calcd. for $C_{20}H_{14}N_3$ 296.1182, found 296.1181.

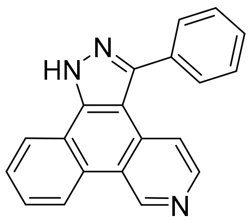

2p

3-Phenyl-1H-benzo[h]pyrazolo[4,3-f]isoquinoline (**2p**). White solid (221 mg, 0.75 mmol, 75% yield). R_f = 0.40 (petroleum ether/ethyl acetate, 1/1 v/v). m.p. 324.3 – 324.8 °C. ^{1}H NMR (400 MHz, DMSO-d_6) δ_H 14.46 – 14.20 (s, 1H), 10.06 – 9.95 (m, 1H), 9.04 – 8.87 (m, 1H), 8.56 – 8.48 (m, 2H), 7.86 – 7.55 (m, 8H). HRMS (ESI IT-TOF) *m/z* $[M + H]^+$ Calcd for $C_{20}H_{14}N_3$ 296.1182, found 296.1181. The ^{13}C NMR spectroscopic data could not be recorded due to the poor solubility in deuterated solvents, such as DMSO-d_6, $CDCl_3$.

B. Single crystal structures

C-1. The X-ray crystal structural data of 2a.

Crystal Structure of $C_{21}H_{14}N_2$

The low temperature (173±2°K) single-crystal X-ray experiments were performed on a SuperNova diffractometer with Cu K_α radiation. Unit cell was obtained and refined by 5063 reflections with 4.7° < θ < 73.2°. No decay was observed in data collection. Raw intensities were corrected for Lorentz and polarization effects, and for absorption by empirical method. Direct phase determination yielded the positions of all non-hydrogen atoms. All non-hydrogen atoms were subjected to anisotropic refinement. All hydrogen atoms were generated geometrically with C-H bonds of 0.95 Å according to criteria described in the SHELXTL manual (Bruker, 1997). They were included in the refinement with $U_{iso}(H) = 1.2U_{eq}$ of their parent atoms. The final full-matric least-square refinement on F^2 converged with $R1 = 0.0457$ and $wR2 = 0.1101$ for 2492 observed reflections [I ≥ 2σ(I)]. The final difference electron density map shows no features. Details of crystal parameters, data collection and structure refinement are given in Table 1.

Data collection was controlled by CrysAlisPro, Agilent Technologies, Version 1.171.36.32 (Oxford, 2013). Computations were performed using the SHELXTL NT ver. 5.10 program package (Bruker, 1997) on an IBM PC 586 computer. Analytic expressions of atomic scattering factors were employed, and anomalous dispersion corrections were incorporated (*International Tables for X-ray Crystallography*, 1989). Crystal drawings were produced with XP (Bruker, 1997).

References

[42] Bruker. (1997) SHELXTL. Structure Determination Programs, Version 5.10, Bruker AXS Inc.,6300 Enterprise Lane, Madison, WI 53719-1173, USA.

[43] *International Tables for X-ray Crystallography*: (1989) Vol. C (Kluwer Academic Publishers, Dordrecht) Tables 4.2.6.8 and 6.1.1.4.

[44] Oxford. (2013) CrysAlisPro, Agilent Technologies, Version 1.171.36.32, Oxford Diffraction Ltd., 68 Milton Park, Abingdon, Oxfordshire, OX14 4RX, UK.

Table S1. Details of Data Collection, Processing and Structure Refinement

Sample code	**2a**
Molecular formula	$C_{21}H_{14}N_2$
Molecular weight	294.34
Color and habit	orange prism
Crystal size	0.2 × 0.3 × 0.5 mm
Crystal system	monoclinic
Space group	$P2_1/c$ (No. 14)
Unit cell parameters	a = 9.2011(2) Å α = 90.00°
	b = 12.9253(3) Å β = 90.277(2)°
	c = 12.3868(2) Å γ = 90.00°
	V = 1473.11(5) Å^3 Z = 4 F(000) = 616
Density (calcd)	1.327 g/cm^3
Diffractometer	SuperNova, Dual, Cu at home/near, AtlasS2
Radiation	Cu K_α, λ = 1.54178 Å
Temperature	173±2K
Scan type	ω-scan
Data collection range	$-11 < h < 10$, $-15 < k < 16$, $-9 < l < 15$; $\theta_{max} = 73.8°$
Reflections measured	Total: 9738 Unique (n): 2937 Observed [I ≥ 2σ(I)]: 2492
Absorption coefficient	0.609 mm^{-1}
Minimum and maximum transmission	0.927, 1.000
No. of variables, p	212

Weighting scheme $w = \dfrac{1}{\sigma^2(F_o^2) + (0.0306P)^2 + 0.8796P}$ $P = (F_o^2 + 2F_c^2)/3$

$R1 = \dfrac{\Sigma||F_o|-|F_c||}{\Sigma|F_o|}$ (for all reflections) 0.0550 0.0457 (for observed data)

$wR2 = \sqrt{\dfrac{\Sigma[w(F_o^2 - F_c^2)^2]}{\Sigma w(F_o^2)^2}}$ (for all reflections) 0.1139 0.1101 (for observed data)

Goof = S = $\sqrt{\dfrac{\Sigma[w(F_o^2 - F_c^2)^2]}{n - p}}$ 1.166

Largest and mean Δ/σ 0.004, 0.000

Residual extrema in final difference map -0.243 to 0.219 e Å^{-3}

Table S2. Atomic coordinates and equivalent isotropic temperature factors* (Å^2)

Atoms	x	y	z	$U_{eq.}$
N(1)	0.91260(15)	0.53527(12)	0.60136(12)	0.0252(3)
N(2)	0.83731(16)	0.47314(12)	0.53256(12)	0.0244(3)
C(1)	0.82553(18)	0.55159(13)	0.68545(14)	0.0222(4)
C(2)	0.69066(17)	0.49933(13)	0.67016(13)	0.0214(3)
C(3)	0.70567(18)	0.44904(13)	0.57161(13)	0.0218(3)
C(4)	0.59796(18)	0.38125(13)	0.52552(14)	0.0233(4)
C(5)	0.6217(2)	0.32424(14)	0.43111(15)	0.0284(4)
C(6)	0.5170(2)	0.25703(15)	0.39322(16)	0.0352(5)
C(7)	0.3869(2)	0.24664(15)	0.44959(17)	0.0368(5)
C(8)	0.3613(2)	0.30325(15)	0.54149(16)	0.0322(4)
C(9)	0.46544(18)	0.37312(13)	0.58236(14)	0.0247(4)
C(10)	0.43881(18)	0.43705(13)	0.67843(14)	0.0244(4)
C(11)	0.55166(17)	0.49814(13)	0.72439(13)	0.0226(4)
C(12)	0.52156(19)	0.56063(15)	0.81447(15)	0.0288(4)
C(13)	0.3840(2)	0.56544(16)	0.85749(16)	0.0350(5)
C(14)	0.2724(2)	0.50747(16)	0.81138(16)	0.0352(5)
C(15)	0.29954(19)	0.44413(15)	0.72457(16)	0.0310(4)
C(16)	0.88687(17)	0.61370(13)	0.77483(13)	0.0218(4)
C(17)	0.87944(18)	0.58058(14)	0.88180(14)	0.0243(4)
C(18)	0.95271(19)	0.63455(15)	0.96241(14)	0.0283(4)
C(19)	1.0327(2)	0.72182(15)	0.93731(15)	0.0323(4)
C(20)	1.0389(2)	0.75641(15)	0.83176(16)	0.0332(4)
C(21)	0.96607(19)	0.70283(14)	0.75095(15)	0.0272(4)

*$U_{eq.}$ defined as one third of the trace of the orthogonalized **U** tensor.

Table S3. Bond lengths (Å) and bond angles (°)

N(1)-C(1)	1.334(2)	C(9)-C(10)	1.470(3)
N(1)-N(2)	1.358(2)	C(10)-C(15)	1.409(2)
N(2)-C(3)	1.343(2)	C(10)-C(11)	1.421(2)
C(1)-C(2)	1.425(2)	C(11)-C(12)	1.406(2)
C(1)-C(16)	1.477(2)	C(12)-C(13)	1.377(3)
C(2)-C(3)	1.390(2)	C(13)-C(14)	1.392(3)
C(2)-C(11)	1.448(2)	C(14)-C(15)	1.375(3)
C(3)-C(4)	1.439(2)	C(16)-C(17)	1.394(2)
C(4)-C(5)	1.400(2)	C(16)-C(21)	1.396(2)
C(4)-C(9)	1.415(3)	C(17)-C(18)	1.390(2)
C(5)-C(6)	1.378(3)	C(18)-C(19)	1.383(3)
C(6)-C(7)	1.395(3)	C(19)-C(20)	1.383(3)
C(7)-C(8)	1.375(3)	C(20)-C(21)	1.387(3)
C(8)-C(9)	1.409(2)		
C(1)-N(1)-N(2)	106.08(14)	C(4)-C(9)-C(10)	120.56(15)
C(3)-N(2)-N(1)	111.67(14)	C(15)-C(10)-C(11)	117.76(16)
N(1)-C(1)-C(2)	110.32(15)	C(15)-C(10)-C(9)	121.36(16)
N(1)-C(1)-C(16)	116.26(15)	C(11)-C(10)-C(9)	120.80(15)
C(2)-C(1)-C(16)	133.36(16)	C(12)-C(11)-C(10)	119.38(15)
C(3)-C(2)-C(1)	104.37(15)	C(12)-C(11)-C(2)	122.68(16)
C(3)-C(2)-C(11)	119.61(15)	C(10)-C(11)-C(2)	117.74(15)
C(1)-C(2)-C(11)	135.59(16)	C(13)-C(12)-C(11)	121.20(17)
N(2)-C(3)-C(2)	107.54(15)	C(12)-C(13)-C(14)	119.65(19)
N(2)-C(3)-C(4)	128.24(16)	C(15)-C(14)-C(13)	120.33(17)
C(2)-C(3)-C(4)	124.19(15)	C(14)-C(15)-C(10)	121.66(17)
C(5)-C(4)-C(9)	120.99(16)	C(17)-C(16)-C(21)	118.88(16)
C(5)-C(4)-C(3)	122.81(16)	C(17)-C(16)-C(1)	121.67(15)
C(9)-C(4)-C(3)	116.19(15)	C(21)-C(16)-C(1)	119.18(15)
C(6)-C(5)-C(4)	120.30(18)	C(18)-C(17)-C(16)	120.18(16)
C(5)-C(6)-C(7)	119.36(18)	C(19)-C(18)-C(17)	120.31(17)
C(8)-C(7)-C(6)	120.94(18)	C(20)-C(19)-C(18)	120.03(17)
C(7)-C(8)-C(9)	121.32(18)	C(19)-C(20)-C(21)	119.91(18)
C(8)-C(9)-C(4)	117.06(17)	C(20)-C(21)-C(16)	120.67(17)
C(8)-C(9)-C(10)	122.37(16)		
Hydrogen bonding			
H(2)···N(1)$^{\#1}$	2.11(2)	N(2)-H(2)···N(1)$^{\#1}$	139.7(19)

Symmetry transformations code: #1 (2-*x*, 1-*y*, 1-*z*).

Table S4. Anisotropic thermal parameters* (Å^2)

Atoms	U_{11}	U_{22}	U_{33}	U_{23}	U_{13}	U_{12}
N(1)	0.0212(7)	0.0282(8)	0.0262(7)	-0.0034(6)	0.0006(6)	-0.0016(6)
N(2)	0.0215(7)	0.0284(8)	0.0233(7)	-0.0042(6)	0.0017(6)	-0.0016(6)
C(1)	0.0181(8)	0.0238(8)	0.0248(8)	0.0004(7)	0.0020(7)	0.0010(6)
C(2)	0.0188(8)	0.0211(8)	0.0244(8)	0.0017(6)	0.0001(7)	0.0010(6)
C(3)	0.0188(8)	0.0222(8)	0.0244(8)	0.0024(6)	0.0011(7)	0.0018(6)
C(4)	0.0236(8)	0.0196(8)	0.0265(9)	0.0031(6)	-0.0041(7)	0.0002(6)
C(5)	0.0304(9)	0.0258(9)	0.0289(9)	-0.0001(7)	-0.0032(8)	0.0010(7)
C(6)	0.0449(12)	0.0264(9)	0.0343(10)	-0.0050(8)	-0.0103(9)	-0.0005(8)
C(7)	0.0377(11)	0.0290(10)	0.0437(11)	-0.0005(8)	-0.0101(9)	-0.0108(8)
C(8)	0.0278(9)	0.0289(9)	0.0399(11)	0.0045(8)	-0.0037(8)	-0.0071(7)
C(9)	0.0228(8)	0.0206(8)	0.0307(9)	0.0064(7)	-0.0046(7)	-0.0015(7)
C(10)	0.0207(8)	0.0233(8)	0.0290(9)	0.0070(7)	-0.0001(7)	-0.0002(6)
C(11)	0.0184(8)	0.0230(8)	0.0263(9)	0.0050(7)	0.0015(7)	0.0011(6)
C(12)	0.0227(8)	0.0331(10)	0.0305(10)	-0.0022(8)	0.0022(7)	0.0007(7)
C(13)	0.0287(10)	0.0416(11)	0.0346(10)	-0.0016(8)	0.0072(8)	0.0041(8)
C(14)	0.0213(9)	0.0433(11)	0.0409(11)	0.0061(9)	0.0101(8)	0.0010(8)
C(15)	0.0216(9)	0.0330(10)	0.0385(10)	0.0086(8)	-0.0002(8)	-0.0054(7)
C(16)	0.0155(7)	0.0251(8)	0.0249(8)	-0.0022(7)	0.0030(6)	0.0015(6)
C(17)	0.0190(8)	0.0261(9)	0.0280(9)	0.0007(7)	0.0029(7)	0.0012(7)
C(18)	0.0258(9)	0.0348(10)	0.0242(9)	-0.0002(7)	0.0010(7)	0.0051(7)
C(19)	0.0286(9)	0.0365(10)	0.0319(10)	-0.0095(8)	-0.0030(8)	-0.0025(8)
C(20)	0.0319(10)	0.0310(10)	0.0367(10)	-0.0035(8)	0.0030(8)	-0.0099(8)
C(21)	0.0265(9)	0.0296(9)	0.0255(9)	-0.0002(7)	0.0052(7)	-0.0038(7)

*The exponent takes the form: $-2\pi^2\Sigma\Sigma U_{ij}h_ih_j\mathbf{a}_i^*\mathbf{a}_j^*$

Table S5. Coordinates and isotropic temperature factors* (Å^2) for H atoms

Atoms	*x*	*y*	*z*	$U_{eq.}$
H(2)	0.882(2)	0.4547(17)	0.4717(18)	0.035(6)
H(5)	0.7104	0.3319	0.3930	0.034
H(6)	0.5333	0.2181	0.3293	0.042
H(7)	0.3150	0.1998	0.4241	0.044
H(8)	0.2716	0.2951	0.5782	0.039
H(12)	0.5975	0.6002	0.8462	0.035
H(13)	0.3654	0.6081	0.9183	0.042
H(14)	0.1770	0.5117	0.8400	0.042
H(15)	0.2224	0.4041	0.6950	0.037
H(17)	0.8241	0.5210	0.8996	0.029
H(18)	0.9478	0.6114	1.0351	0.034
H(19)	1.0834	0.7580	0.9926	0.039
H(20)	1.0930	0.8168	0.8146	0.040
H(21)	0.9701	0.7270	0.6786	0.033

*The exponent takes the form: $-8\pi^2 U \sin^2\theta/\lambda^2$

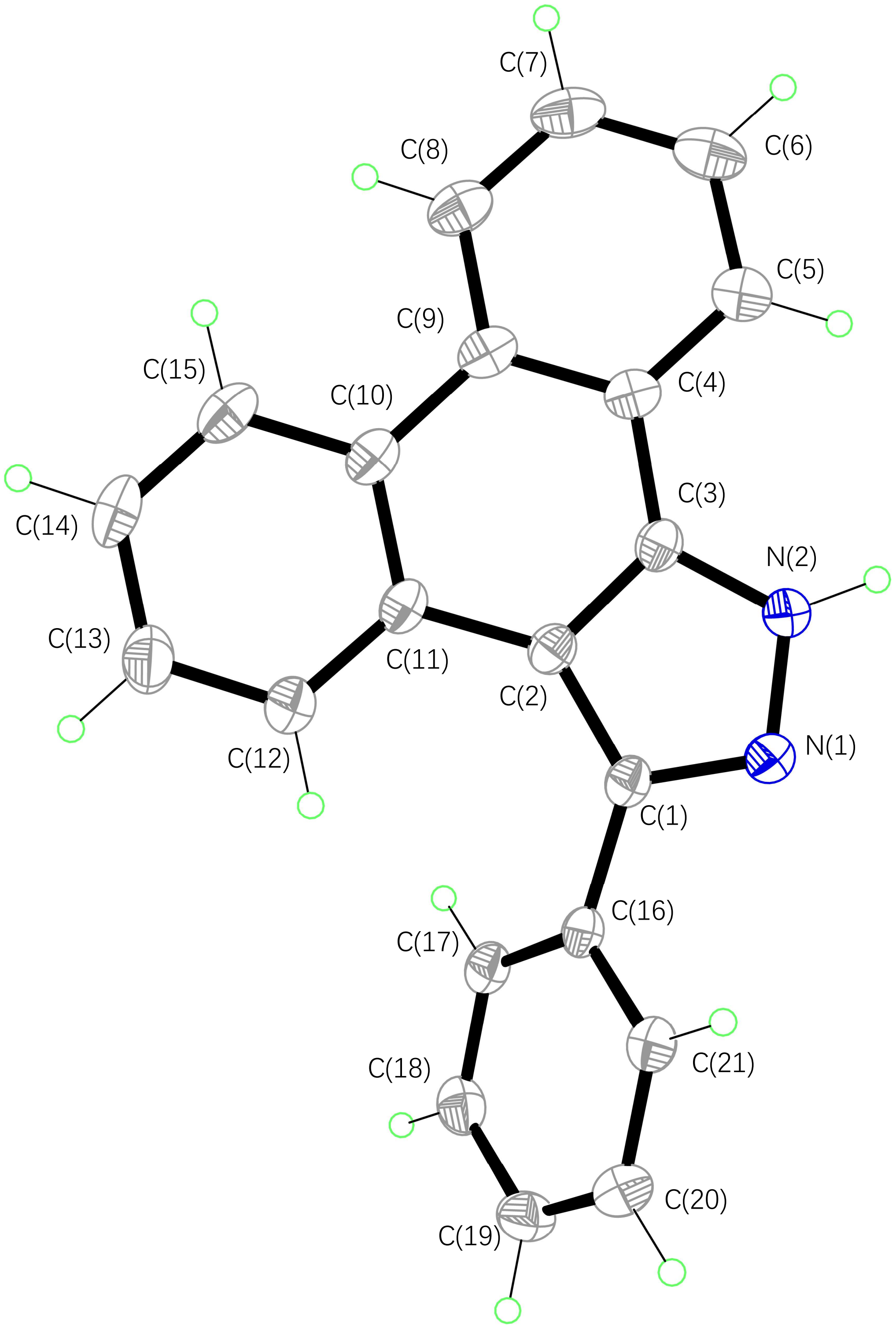

ORTEP drawing of $C_{21}H_{14}N_2$ with 50% probability ellipsoids, showing the atomic numbering scheme.

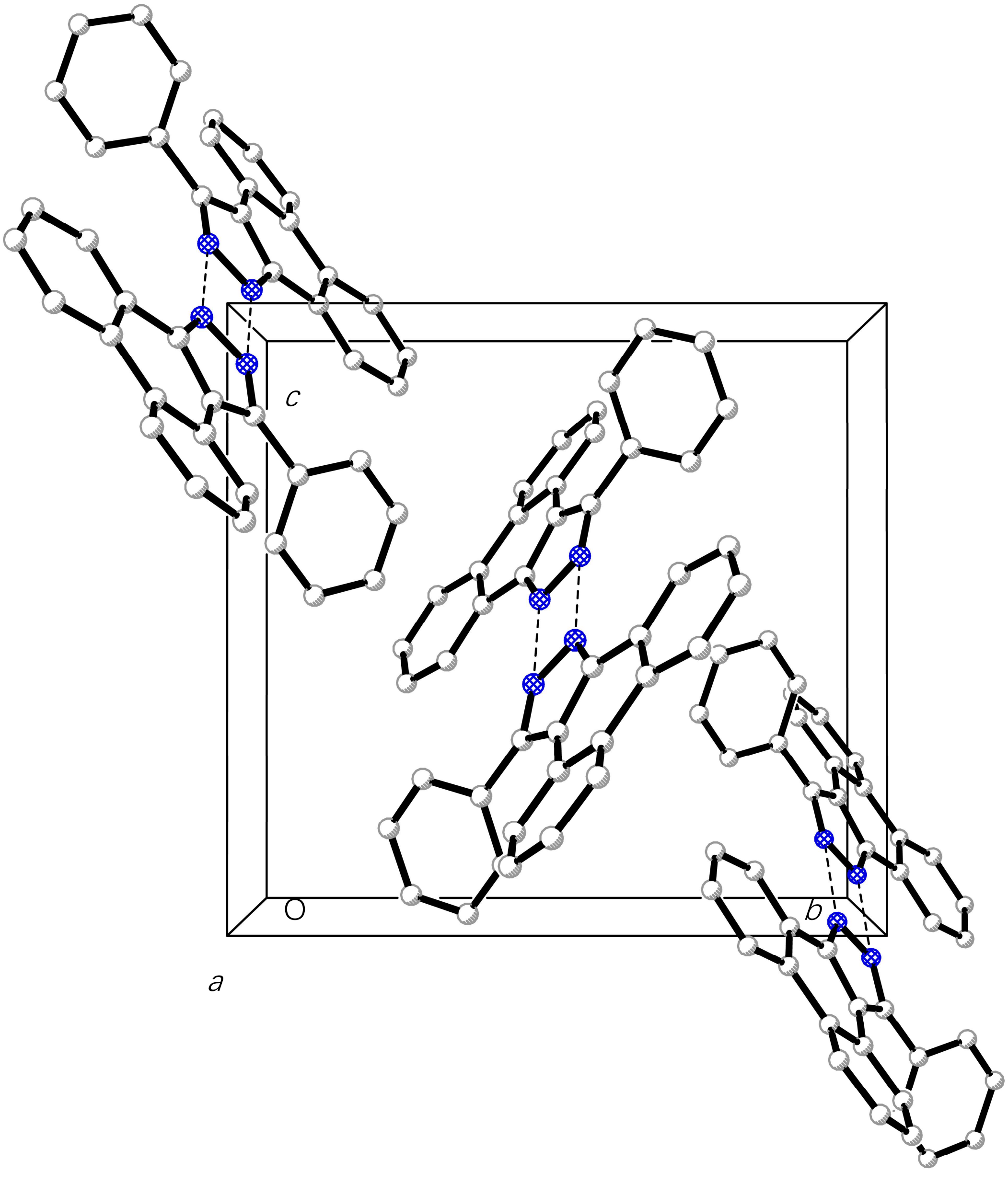

A packing view along the *a* direction

C. Computational details

(1) **2a**

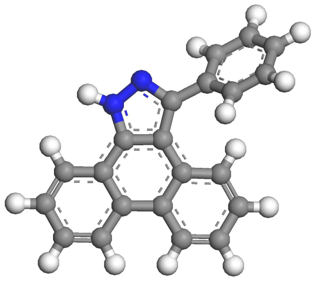

Name: **2a**

Charge: 0

Multiplicity: 1

E(RB3LYP) = -918.636331365 Ha

C	2.309899	-1.082416	0.028084
C	2.683573	0.285514	0.020939
C	1.660850	1.333693	-0.055064
C	0.275559	1.001357	-0.082205
C	-0.088967	-0.392068	-0.003594
C	0.908532	-1.366913	0.009304
C	3.285343	-2.091933	0.065633
C	4.624468	-1.769515	0.104446
C	5.009521	-0.424504	0.110201
C	4.060039	0.576450	0.069600
C	2.015187	2.693672	-0.128026
C	1.064883	3.687913	-0.235848
C	-0.290540	3.352976	-0.288836
C	-0.673005	2.030447	-0.215177
C	-1.313288	-1.131070	0.009679
N	-1.059871	-2.438856	0.009742
N	0.282518	-2.564947	0.008808
C	-2.716410	-0.681166	0.045894
C	-3.160500	0.207478	1.028692
C	-4.491827	0.599551	1.075717
C	-5.399777	0.108113	0.142449
C	-4.968222	-0.781787	-0.835241
C	-3.635969	-1.175223	-0.882618
H	2.978980	-3.130409	0.066454
H	5.372294	-2.551393	0.133433
H	6.059529	-0.163549	0.146165
H	4.398067	1.601897	0.076617
H	3.055891	2.981341	-0.109755
H	1.373929	4.723978	-0.289753
H	-1.040654	4.127065	-0.390807
H	-1.720676	1.779419	-0.268592
H	0.695592	-3.484659	0.030864

H	-2.460495	0.587097	1.761538
H	-4.821845	1.285936	1.845323
H	-6.436871	0.416852	0.178061
H	-5.668387	-1.167760	-1.565512
H	-3.299983	-1.862605	-1.648059

(2) **2a** tautomer

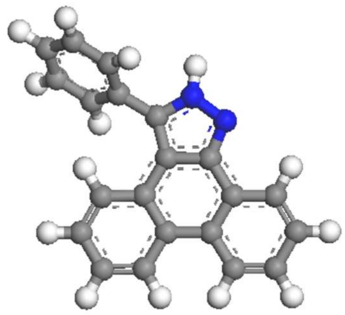

Name: **2a** tautomer

Charge: 0

Multiplicity: 1

E(RB3LYP) = -918.635057168 Ha

C	2.298709	-1.114348	0.029561
C	2.699089	0.243326	0.023098
C	1.697682	1.318618	-0.055161
C	0.306775	1.021449	-0.080787
C	-0.089918	-0.365417	-0.001513
C	0.885486	-1.394717	0.009177
C	3.255403	-2.138995	0.069957
C	4.602056	-1.842962	0.111644
C	5.012719	-0.506817	0.117703
C	4.079860	0.510756	0.074480
C	2.084038	2.668416	-0.132200
C	1.157722	3.686584	-0.241830
C	-0.204340	3.385199	-0.291624
C	-0.617073	2.070918	-0.213861
C	-1.316939	-1.042517	0.008900
N	-0.994333	-2.357270	0.002880
N	0.324832	-2.608514	0.011069
C	-2.723982	-0.627983	0.043047
C	-3.178415	0.283209	1.001039
C	-4.517266	0.645672	1.045895
C	-5.421935	0.099790	0.139910
C	-4.980176	-0.814637	-0.809859
C	-3.639951	-1.177715	-0.859304
H	-1.648489	-3.126384	0.038345
H	2.920511	-3.167875	0.070740
H	5.334775	-2.639295	0.143033
H	6.067172	-0.264181	0.155888
H	4.436191	1.530048	0.081943

H	3.131265	2.931116	-0.116141
H	1.492581	4.714474	-0.299131
H	-0.936321	4.176405	-0.394156
H	-1.670961	1.845117	-0.265103
H	-2.480919	0.699029	1.715862
H	-4.856512	1.350832	1.794052
H	-6.465585	0.385276	0.175541
H	-5.677922	-1.241884	-1.518882
H	-3.296528	-1.879174	-1.609264

(3) **2a**·dimer-*anti*

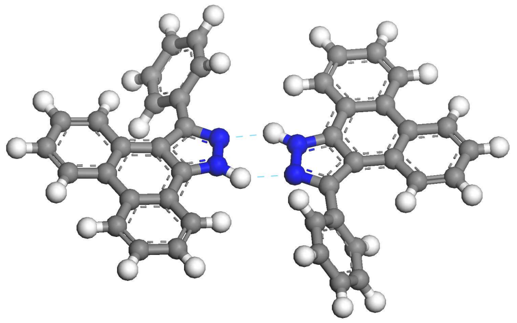

Name: **2a**·dimer-*anti*

Charge: 0

Multiplicity: 1

E(RB3LYP) = -1837.28624315 Ha

Corrected Total Energy = -1837.28587965 Ha

C	4.583550	4.643425	0.206237
C	5.364129	3.504986	0.203692
C	4.792087	2.219548	0.155280
C	3.377517	2.142633	0.103416
C	2.593015	3.307637	0.109247
C	3.188267	4.549284	0.160869
C	5.600905	0.995550	0.175731
C	4.986004	-0.289034	0.118007
C	3.549419	-0.348751	0.002127
C	2.806369	0.833664	0.035089
C	7.003178	1.051684	0.279548
C	7.774352	-0.090487	0.344457
C	7.162266	-1.346165	0.318918
C	5.790985	-1.437853	0.208208
C	2.569328	-1.385464	-0.093354
N	1.348659	-0.851971	-0.091189
N	1.505131	0.484827	-0.014516
C	2.685823	-2.848484	-0.216346
C	3.504029	-3.428619	-1.189243
C	3.558691	-4.808885	-1.332313
C	2.794301	-5.629201	-0.507983
C	1.971622	-5.060193	0.458534

C	1.916784	-3.679418	0.603452	C	-4.986031	0.289136	0.117369
H	5.058139	5.615603	0.244715	C	-3.549428	0.348719	0.001628
H	6.436955	3.620930	0.241222	C	-2.806485	-0.833759	0.034741
H	1.513836	3.227424	0.070399	C	-7.003339	-1.051394	0.278788
H	2.580480	5.444807	0.164354	C	-7.774419	0.090849	0.343539
H	7.502354	2.008459	0.319043	C	-7.162219	1.346472	0.317981
H	8.850830	-0.009246	0.424681	C	-5.790919	1.438032	0.207409
H	7.759538	-2.246521	0.388276	C	-2.569242	1.385341	-0.093847
H	5.324987	-2.410784	0.200151	N	-1.348621	0.851743	-0.091487
H	0.663544	1.059545	-0.035157	N	-1.505211	-0.485037	-0.014774
H	4.088067	-2.794063	-1.842804	C	-2.685583	2.848360	-0.217002
H	4.193917	-5.244416	-2.093318	C	-3.503634	3.428479	-1.190039
H	2.838227	-6.705111	-0.620290	C	-3.558129	4.808736	-1.333264
H	1.373336	-5.691922	1.103038	C	-2.793726	5.629057	-0.508952
H	1.280582	-3.237428	1.359064	C	-1.971202	5.060064	0.457706
C	-4.584020	-4.643353	0.205922	C	-1.916531	3.679299	0.602780
C	-5.364498	-3.504845	0.203230	H	-5.058699	-5.615486	0.244403
C	-4.792338	-2.219460	0.154812	H	-6.437339	-3.620692	0.240648
C	-3.377756	-2.142673	0.103092	H	-1.514167	-3.227627	0.070330
C	-2.593356	-3.307746	0.109070	H	-2.581017	-5.444913	0.164298
C	-3.188724	-4.549337	0.160700	H	-7.502604	-2.008122	0.318294
C	-5.601051	-0.995390	0.175112	H	-8.850913	0.009710	0.423654

H	-7.759419	2.246886	0.387215
H	-5.324835	2.410921	0.199338
H	-0.663670	-1.059822	-0.035241
H	-4.087675	2.793917	-1.843590
H	-4.193234	5.244254	-2.094377
H	-2.837522	6.704959	-0.621383
H	-1.372905	5.691797	1.102196
H	-1.280447	3.237321	1.358498

(4) **2a**/**2a** tautomer dimer-syn

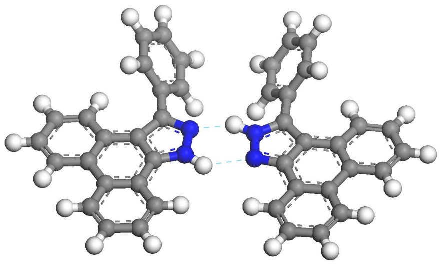

Name: **2a**/**2a** tautomer dimer-syn

Charge: 0

Multiplicity: 1

E(RB3LYP) = -1837.28530929 Ha

Corrected Total Energy = -1837.28493018 Ha

C	-5.000091	-0.110882	-0.068101
C	-5.594495	-1.404976	-0.024132
C	-4.763391	-2.612980	0.018641
C	-3.349223	-2.512924	-0.011705
C	-2.797565	-1.193750	-0.038816
C	-3.561183	-0.023988	-0.025424
C	-5.827563	1.019443	-0.184989
C	-7.200697	0.901868	-0.220988
C	-7.791714	-0.361622	-0.140365
C	-6.999123	-1.487180	-0.049865
C	-5.315362	-3.906939	0.070029
C	-4.517273	-5.032844	0.086672
C	-3.123590	-4.916688	0.049698
C	-2.547556	-3.665877	0.000558
N	-1.501333	-0.817963	-0.071408
N	-1.372370	0.523935	-0.063525
C	-2.600733	1.034479	-0.027211
C	-2.738882	2.499677	0.038344
C	-3.519311	3.106564	1.025956
C	-3.592274	4.490466	1.114147
C	-2.884713	5.287555	0.219173
C	-2.099762	4.691853	-0.762197
C	-2.025892	3.307439	-0.851738

H	-0.654280	-1.378303	-0.066345	C	4.666103	-4.953255	0.165054
H	-5.378479	1.997559	-0.258555	C	5.434415	-3.806077	0.131812
H	-7.816145	1.787748	-0.313558	C	7.059341	-1.352406	-0.029664
H	-8.869328	-0.462452	-0.159264	C	7.829980	-0.211740	-0.137021
H	-7.483397	-2.451278	-0.005650	C	7.213299	1.036940	-0.235042
H	-6.386533	-4.039387	0.095271	C	5.836852	1.124513	-0.199697
H	-4.976621	-6.012174	0.127741	C	2.634126	1.064602	-0.046624
H	-2.501833	-5.802459	0.061362	N	1.514553	-0.902627	-0.056039
H	-1.469543	-3.568843	-0.024491	N	1.439186	0.438267	-0.076647
H	-4.058930	2.490944	1.733605	C	2.669379	2.531457	-0.017286
H	-4.196934	4.947406	1.887366	C	3.456958	3.219833	0.910327
H	-2.942996	6.366419	0.288419	C	3.431601	4.606675	0.961921
H	-1.544948	5.305434	-1.460411	C	2.614777	5.326670	0.094921
H	-1.416629	2.845293	-1.616919	C	1.819951	4.649900	-0.823529
C	3.434862	-2.462647	0.024288	C	1.846529	3.262670	-0.881134
C	4.848399	-2.528477	0.058145	H	0.503807	0.857548	-0.066473
C	5.654415	-1.299529	-0.003087	H	1.586713	-3.553171	0.026300
C	5.033901	-0.020258	-0.065981	H	2.671064	-5.772896	0.148352
C	3.591229	0.035871	-0.025121	H	5.151127	-5.919380	0.223666
C	2.827493	-1.158404	-0.023026	H	6.508710	-3.910464	0.162839
C	2.665299	-3.634948	0.053580	H	7.563606	-2.305537	0.027087
C	3.270878	-4.872073	0.123376	H	8.909446	-0.291287	-0.155331

H	7.809095	1.934773	-0.340488
H	5.366926	2.091795	-0.287715
H	4.076199	2.665223	1.602309
H	4.044130	5.126146	1.687887
H	2.596343	6.408221	0.137742
H	1.180034	5.201557	-1.499890
H	1.232781	2.742227	-1.604893

(5) **2a** tautomer-anti

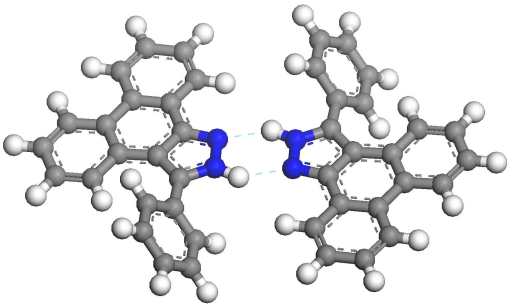

Name: **2a** tautomer-anti

Charge: 0

Multiplicity: 1

E(RB3LYP) = -1837.28452716 Ha

Corrected Total Energy = -1837.28412719 Ha

C	-4.977972	-0.405951	-0.086448
C	-5.632080	0.857751	-0.044812
C	-4.860572	2.108613	0.023399
C	-3.445967	2.079697	0.015655
C	-2.806429	0.791241	-0.014386
C	-3.534764	-0.424241	-0.021059
C	-5.748910	-1.571699	-0.224488
C	-7.126335	-1.519550	-0.286352
C	-7.776259	-0.286366	-0.211704
C	-7.036919	0.874378	-0.099484
C	-5.480166	3.371153	0.080037
C	-4.741045	4.537230	0.121590
C	-3.343500	4.491659	0.106044
C	-2.705254	3.270313	0.053440
N	-1.488441	0.572632	-0.026004
N	-1.370633	-0.765640	-0.036655
C	-2.548302	-1.425801	-0.020683
H	-0.427115	-1.157052	-0.011217
C	-2.545979	-2.892559	0.018369
C	-1.693049	-3.609757	-0.828075
C	-1.635609	-4.995764	-0.760362
C	-2.428746	-5.684782	0.150674
C	-3.275132	-4.978361	0.999998
C	-3.331383	-3.592857	0.938621

H	-5.253423	-2.527617	-0.294805	C	7.036908	-0.874409	0.099488
H	-7.696860	-2.433289	-0.394759	C	5.480129	-3.371167	-0.080034
H	-8.856825	-0.234535	-0.251772	C	4.740995	-4.537237	-0.121565
H	-7.565641	1.815068	-0.061811	C	3.343451	-4.491654	-0.105978
H	-6.557190	3.448702	0.092045	C	2.705218	-3.270301	-0.053362
H	-5.251196	5.491082	0.166591	N	1.488433	-0.572610	0.026090
H	-2.767163	5.407423	0.137923	N	1.370637	0.765665	0.036717
H	-1.624295	3.216884	0.046438	C	2.548311	1.425815	0.020698
H	-1.082457	-3.080334	-1.548446	H	0.427123	1.157088	0.011297
H	-0.973810	-5.537076	-1.424415	C	2.546002	2.892571	-0.018394
H	-2.386594	-6.765334	0.200620	C	1.693100	3.609801	0.828052
H	-3.887092	-5.507015	1.719741	C	1.635673	4.995806	0.760302
H	-3.974382	-3.048270	1.616721	C	2.428793	5.684791	-0.150774
C	4.977974	0.405942	0.086439	C	3.275149	4.978338	-1.000101
C	5.632069	-0.857767	0.044817	C	3.331388	3.592835	-0.938687
C	4.860548	-2.108621	-0.023377	H	5.253451	2.527609	0.294761
C	3.445944	-2.079692	-0.015599	H	7.696886	2.433256	0.394701
C	2.806418	-0.791229	0.014441	H	8.856827	0.234486	0.251757
C	3.534765	0.424246	0.021071	H	7.565619	-1.815105	0.061838
C	5.748926	1.571684	0.224455	H	6.557151	-3.448726	-0.092077
C	7.126351	1.519522	0.286313	H	5.251136	-5.491094	-0.166582
C	7.776261	0.286328	0.211687	H	2.767104	-5.407412	-0.137841

H	1.624260	-3.216861	-0.046335	H	2.386651	6.765342	-0.200748
H	1.082521	3.080405	1.548453	H	3.887095	5.506967	-1.719875
H	0.973897	5.537145	1.424357	H	3.974361	3.048222	-1.616790

D. NMR charts

^{1}H NMR (400 MHz, $CDCl_3$) chart of **S**$_\mathbf{n}$

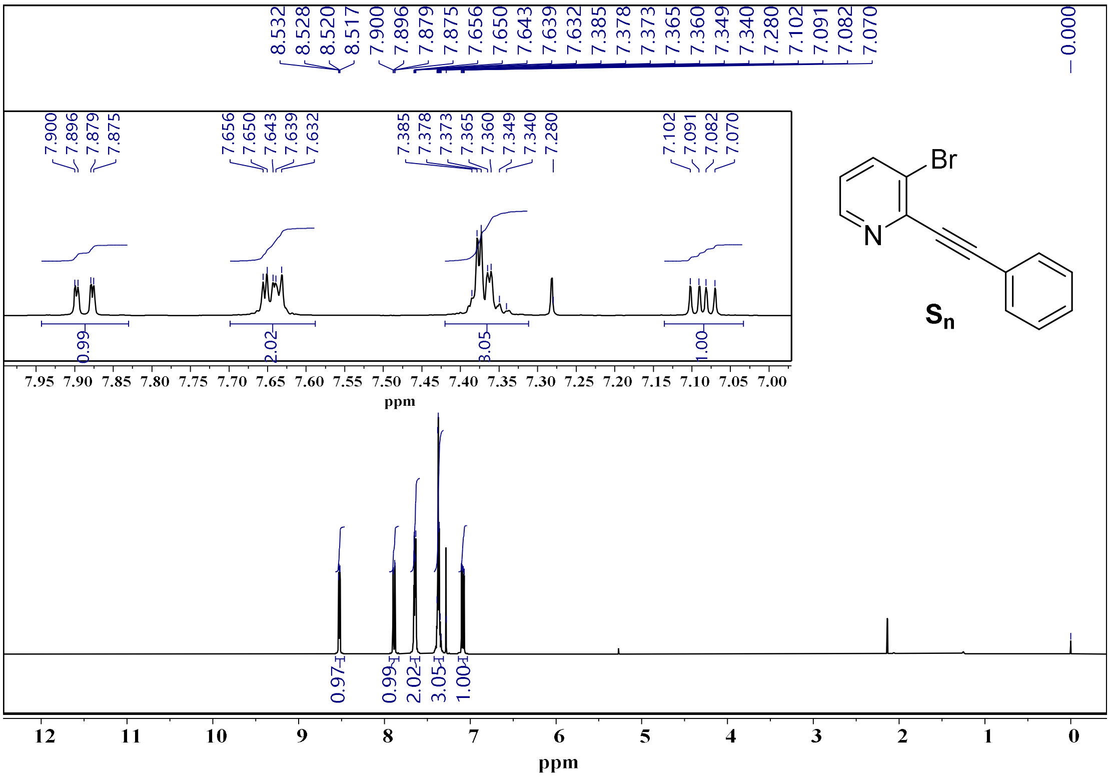

^{13}C{^{1}H} NMR (101 MHz, $CDCl_3$) chart of **S**$_\mathbf{n}$

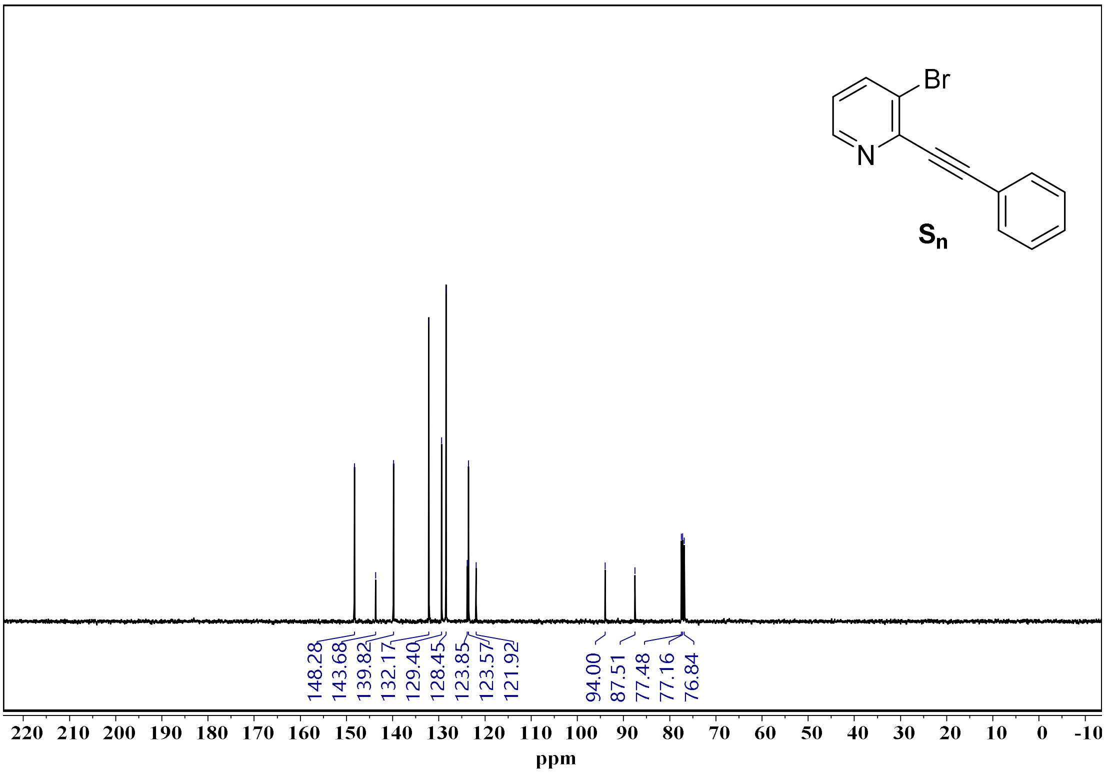

^{1}H NMR (400 MHz, $CDCl_3$) chart of **So**

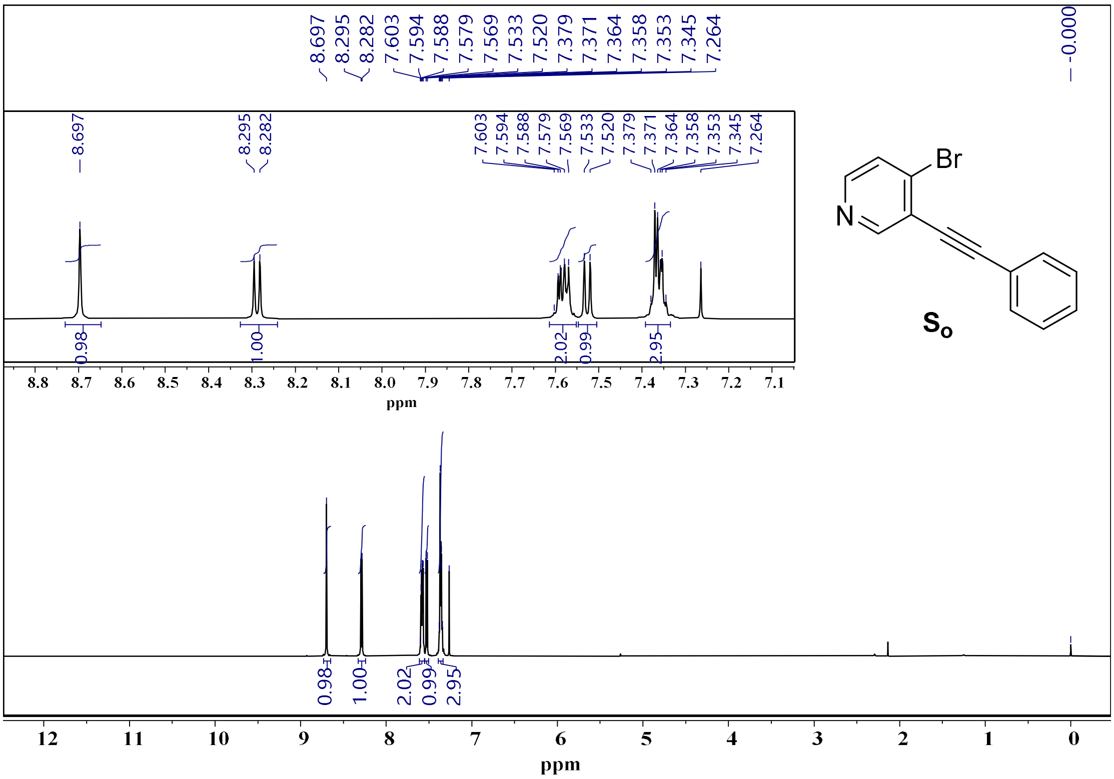

^{13}C{^{1}H} NMR (101 MHz, $CDCl_3$) chart of **So**

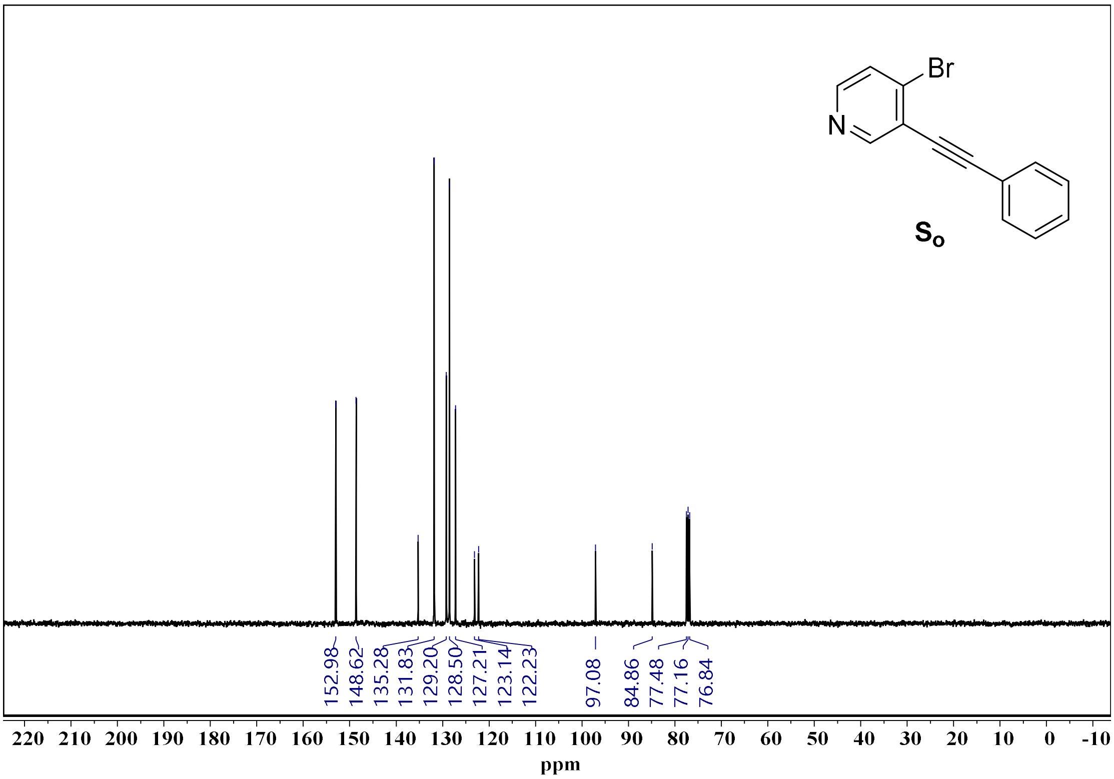

^{1}H NMR (400 MHz, $CDCl_3$) chart of **S_p**

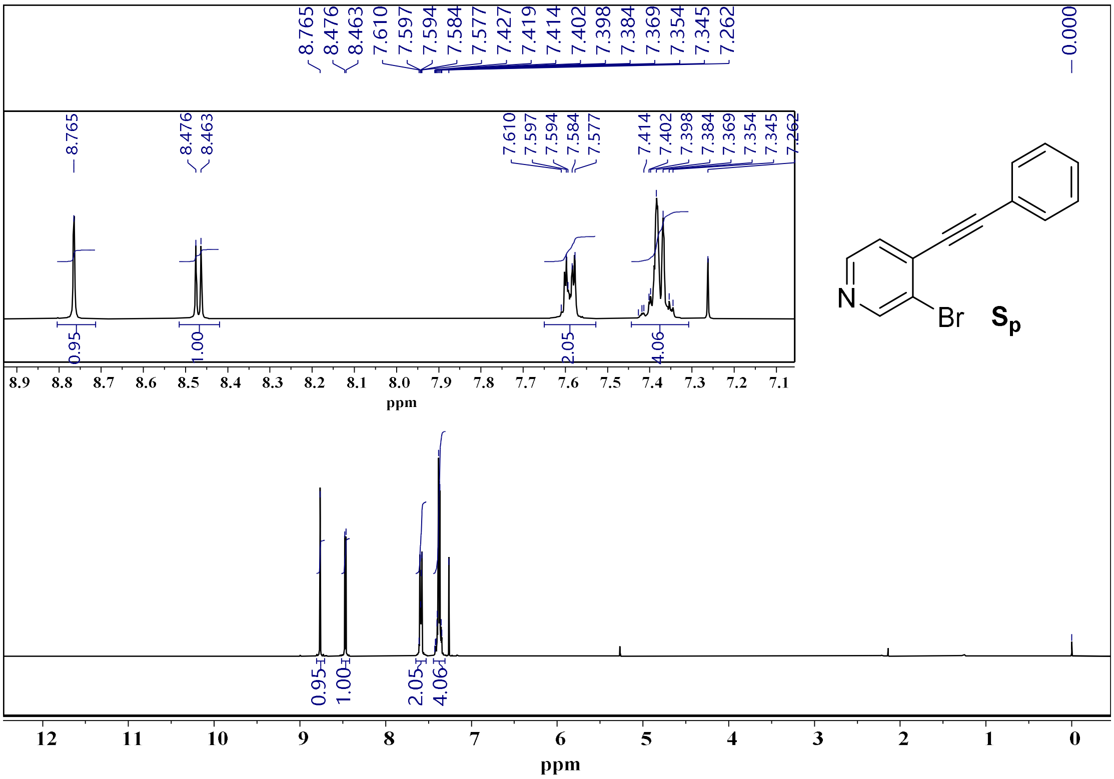

^{13}C{^{1}H} NMR (101 MHz, $CDCl_3$) chart of **S_p**

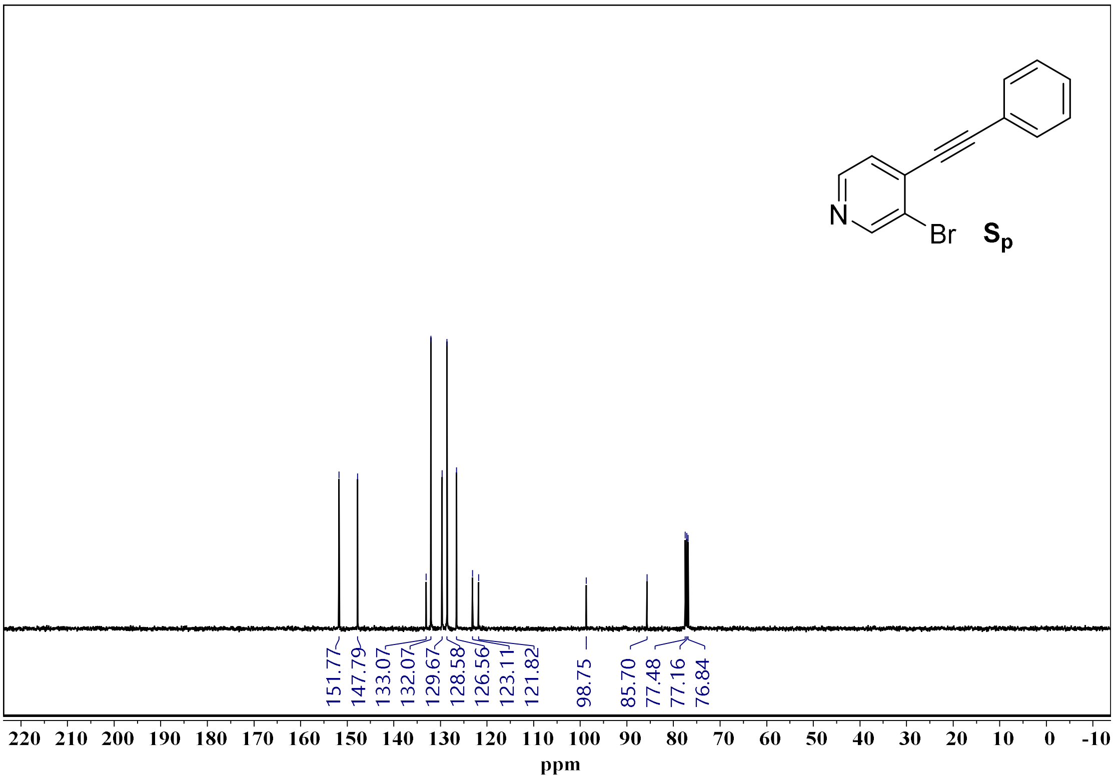

^{1}H NMR (400 MHz, $CDCl_3$) chart of **1a**

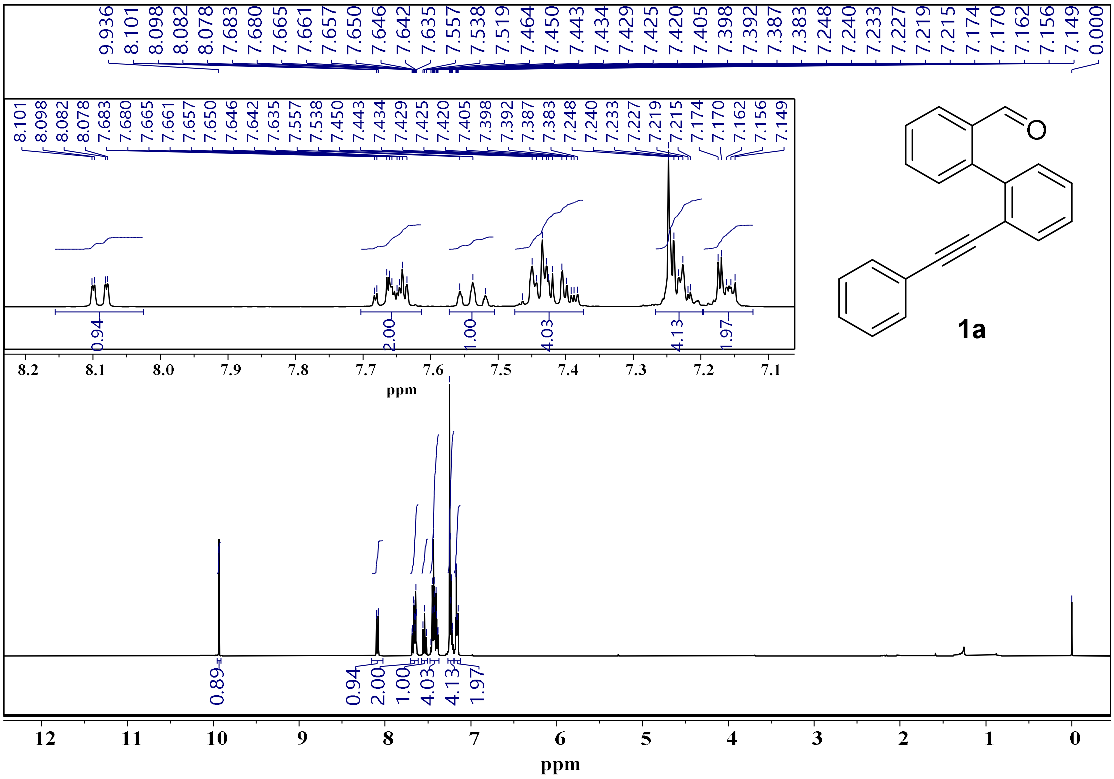

^{13}C{^{1}H} NMR (101 MHz, $CDCl_3$) chart of **1a**

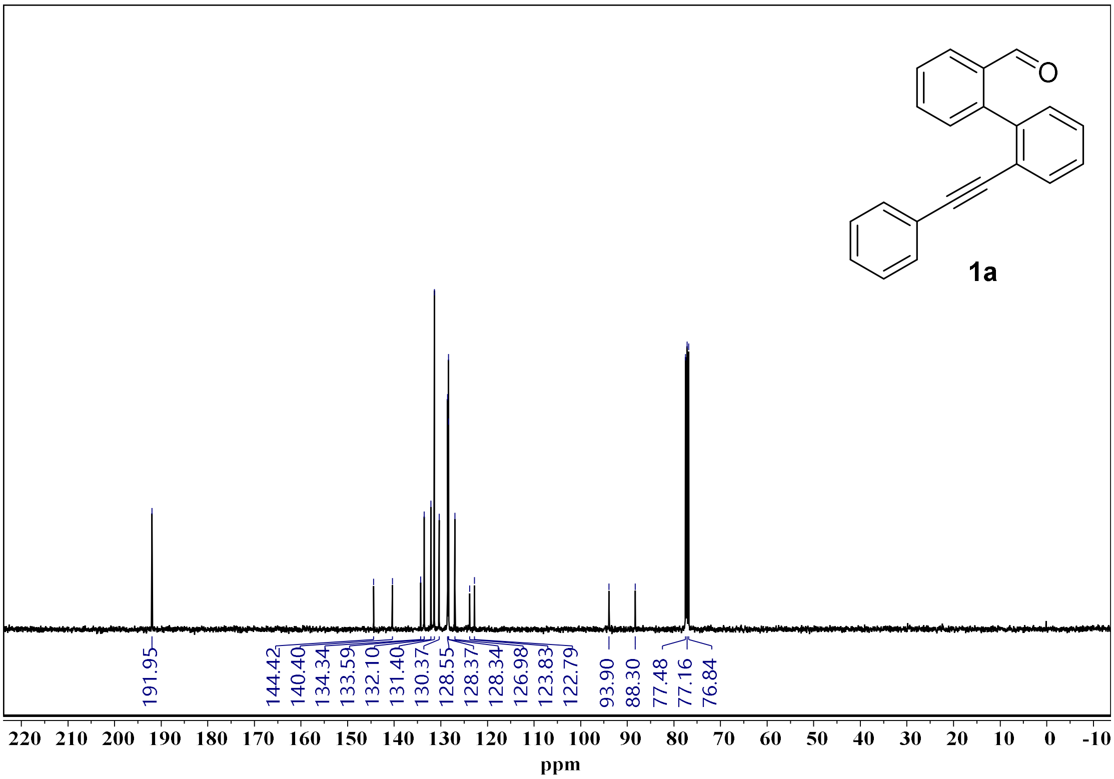

^{1}H NMR (400 MHz, $CDCl_3$) chart of **1b**

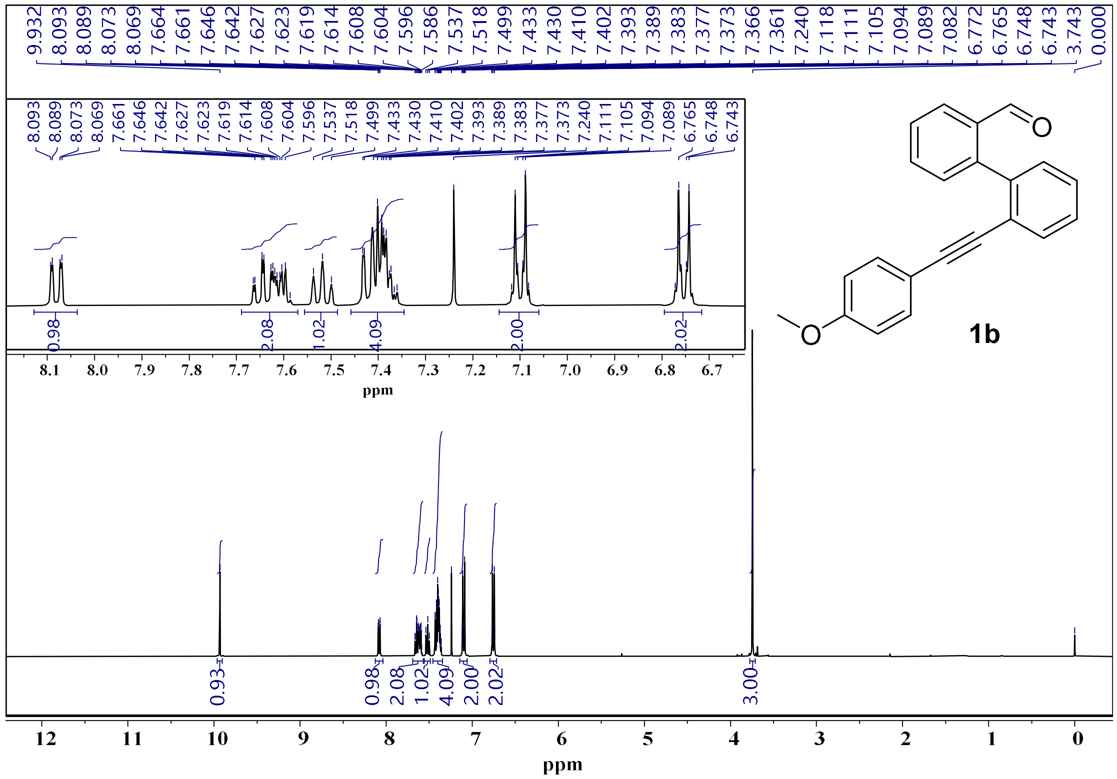

^{13}C{^{1}H} NMR (101 MHz, $CDCl_3$) chart of **1b**

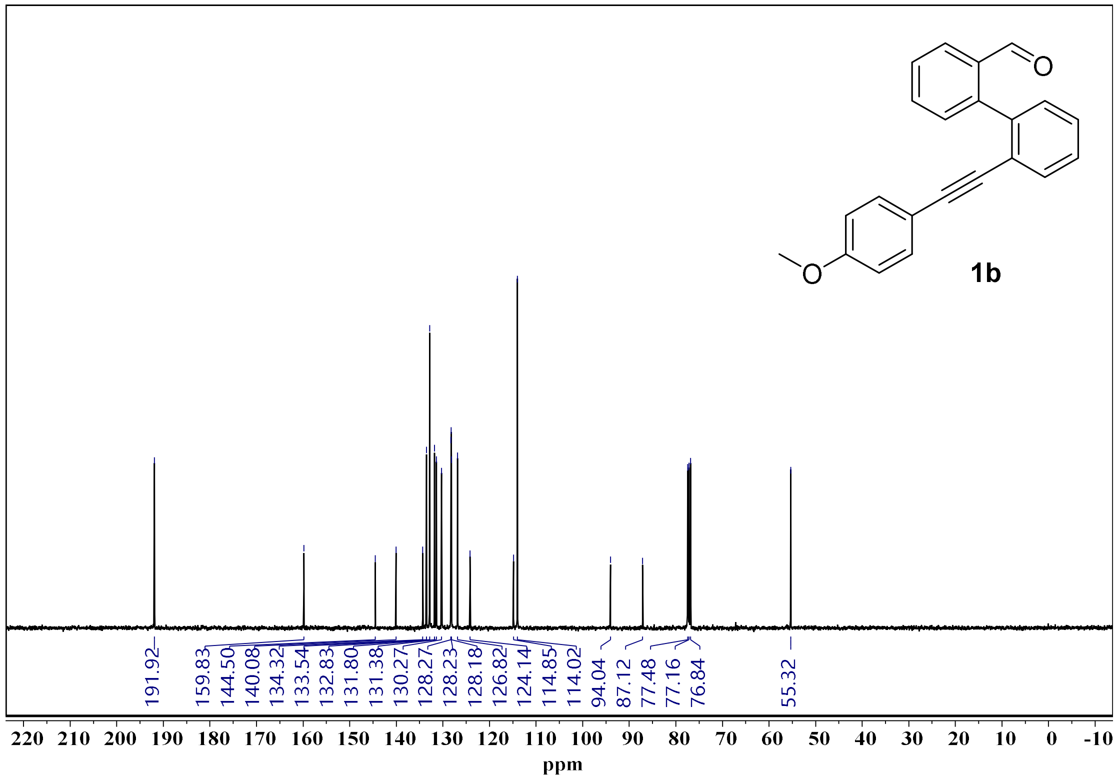

^{1}H NMR (400 MHz, $CDCl_3$) chart of **1c**

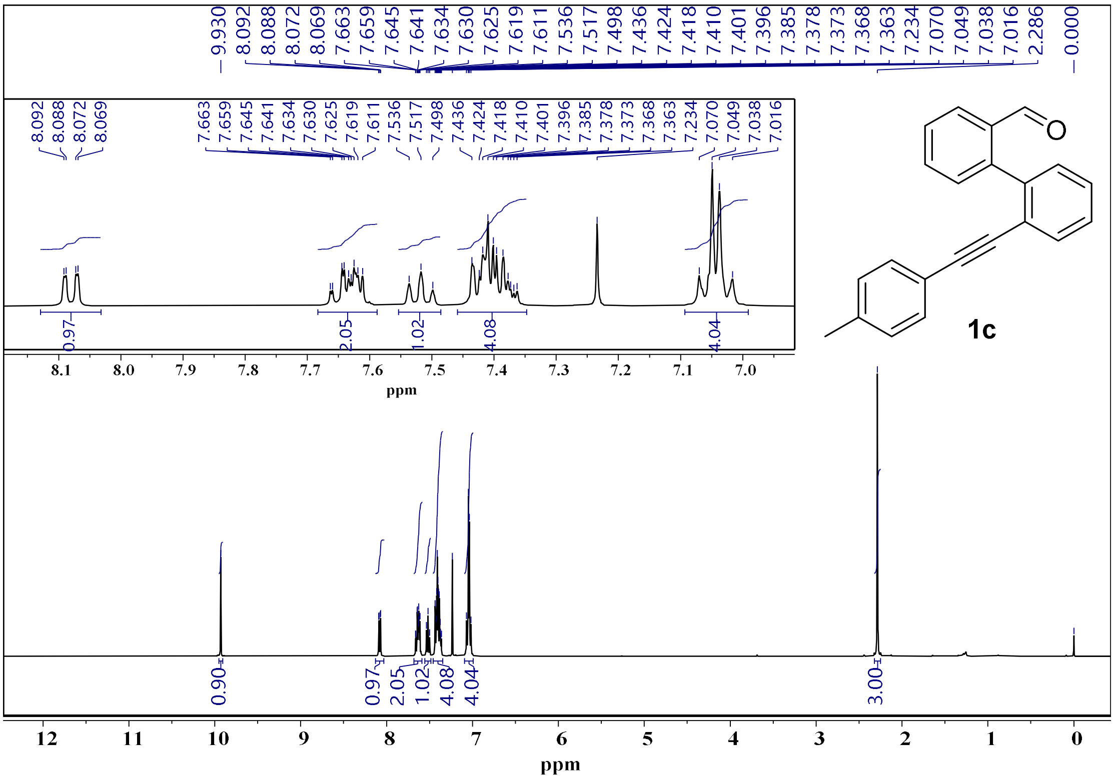

^{13}C{^{1}H} NMR (101 MHz, $CDCl_3$) chart of **1c**

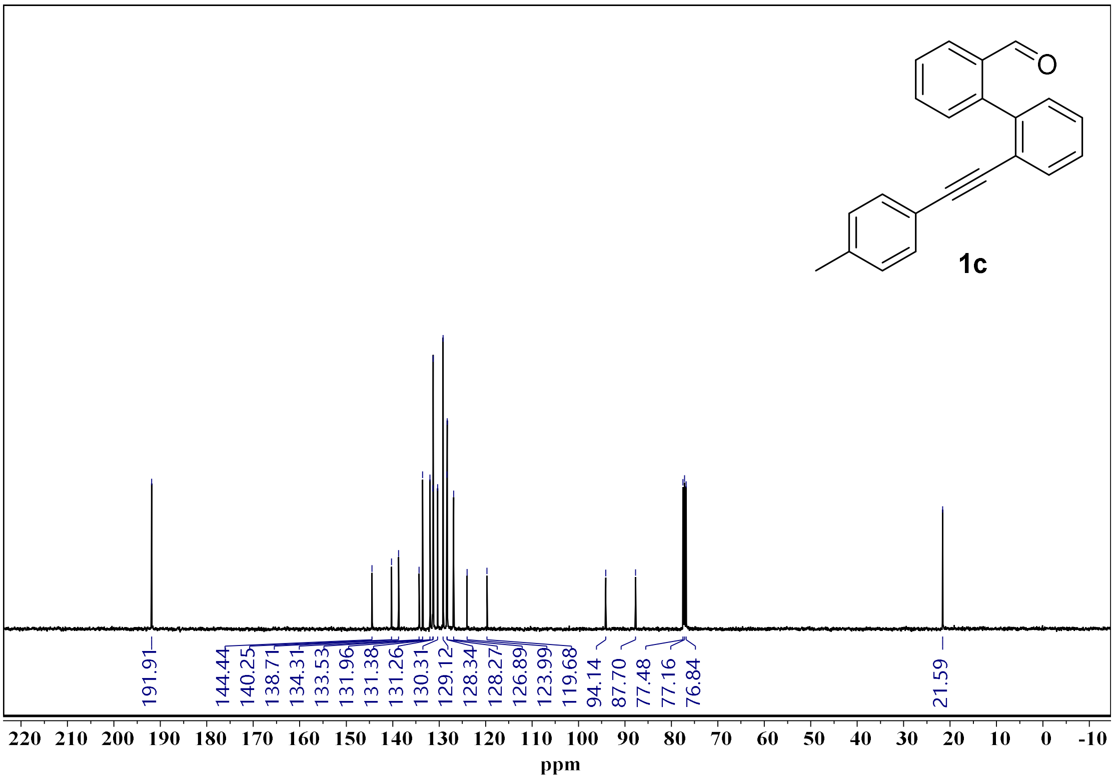

^{1}H NMR (400 MHz, $CDCl_3$) chart of **1d**

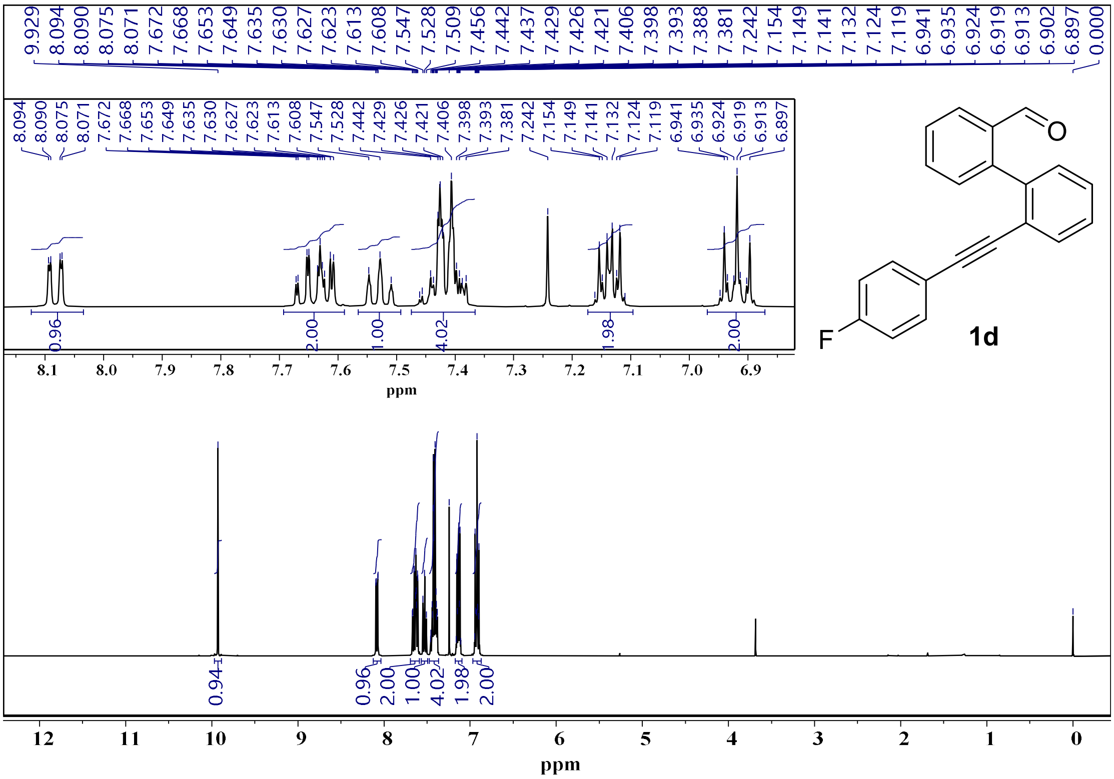

^{13}C{^{1}H} NMR (101 MHz, $CDCl_3$) chart of **1d**

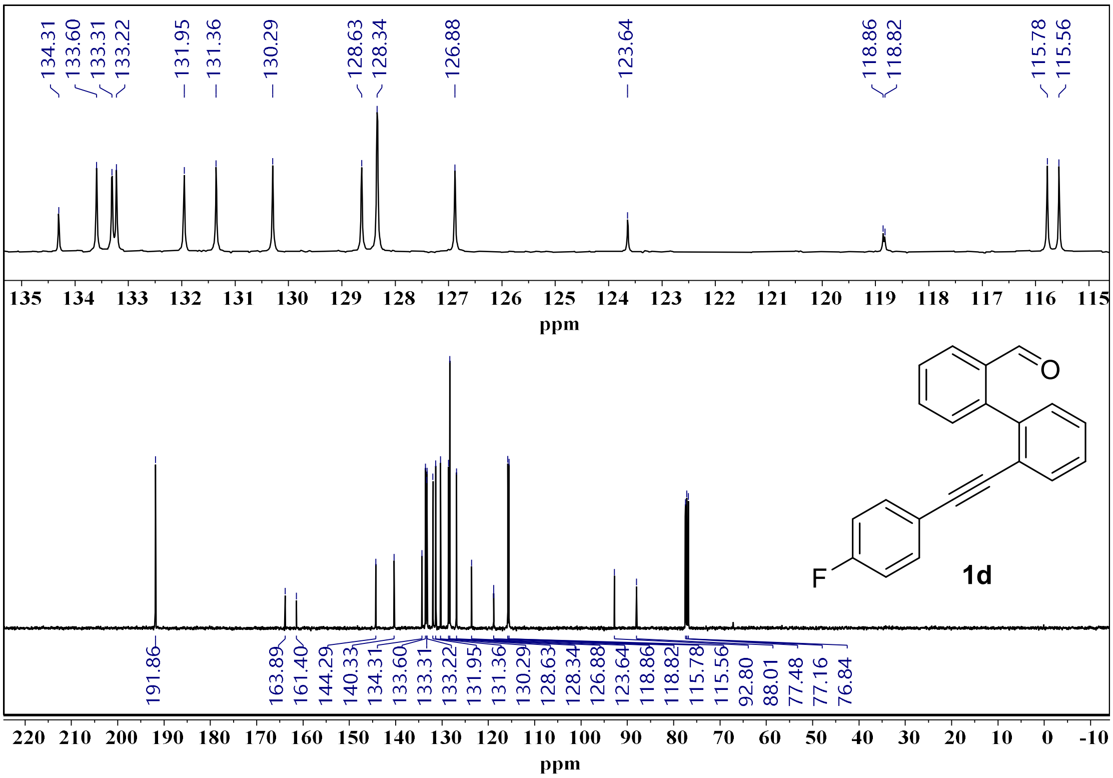

^{1}H NMR (400 MHz, $CDCl_3$) chart of **1e**

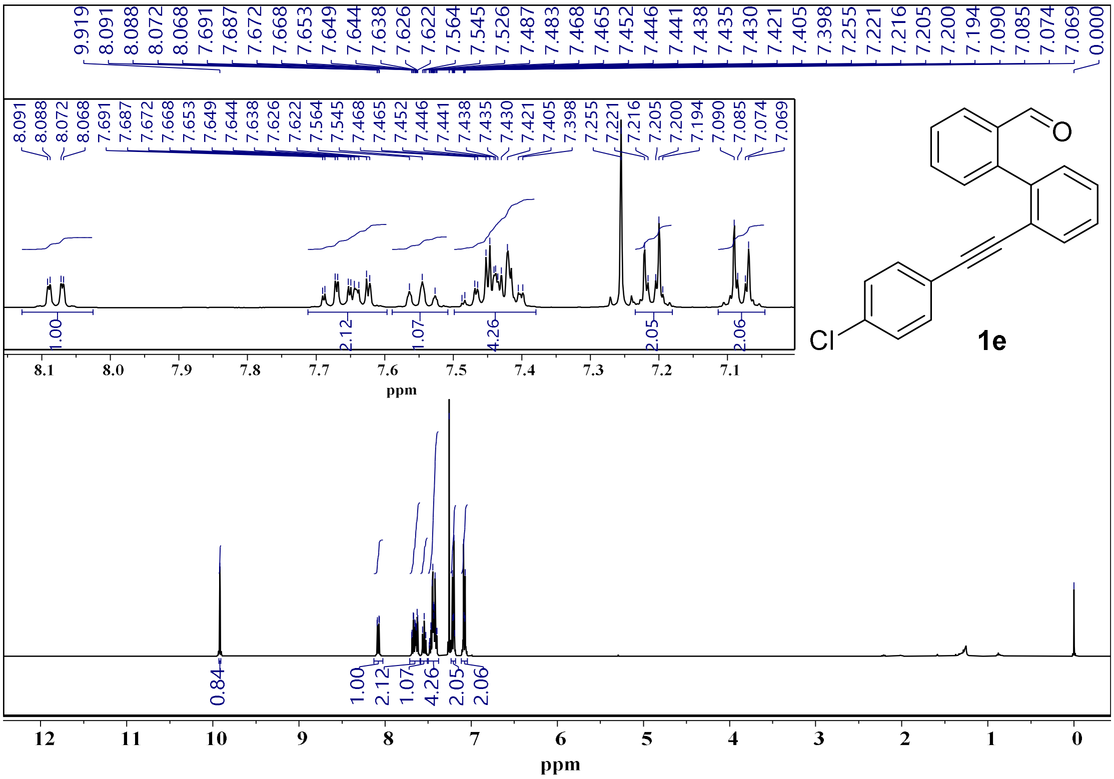

^{13}C{^{1}H} NMR (101 MHz, $CDCl_3$) chart of **1e**

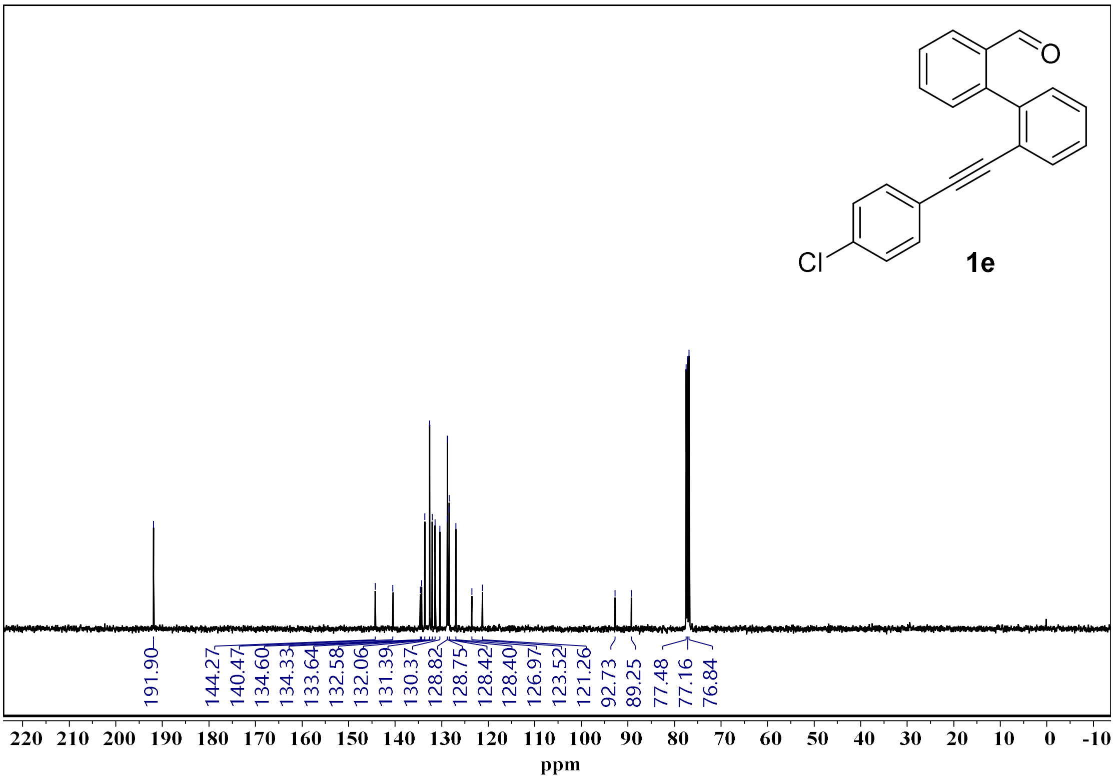

^{1}H NMR (400 MHz, $CDCl_3$) chart of **1f**

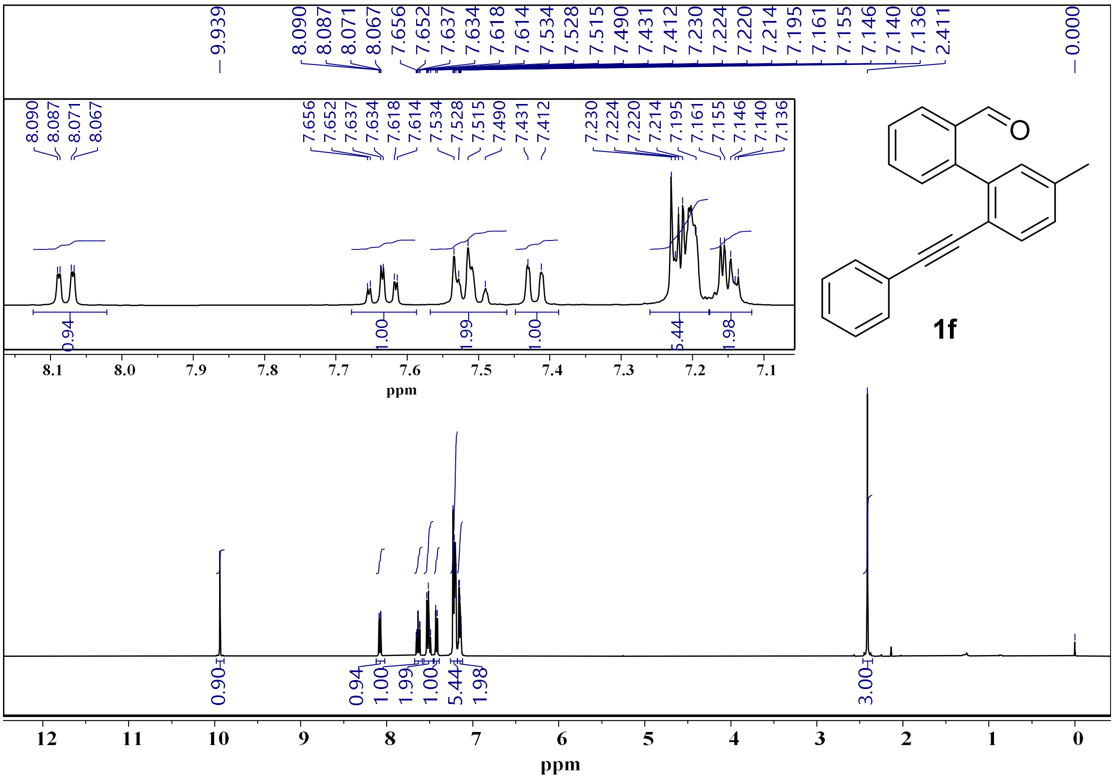

^{13}C{^{1}H} NMR (101 MHz, $CDCl_3$) chart of **1f**

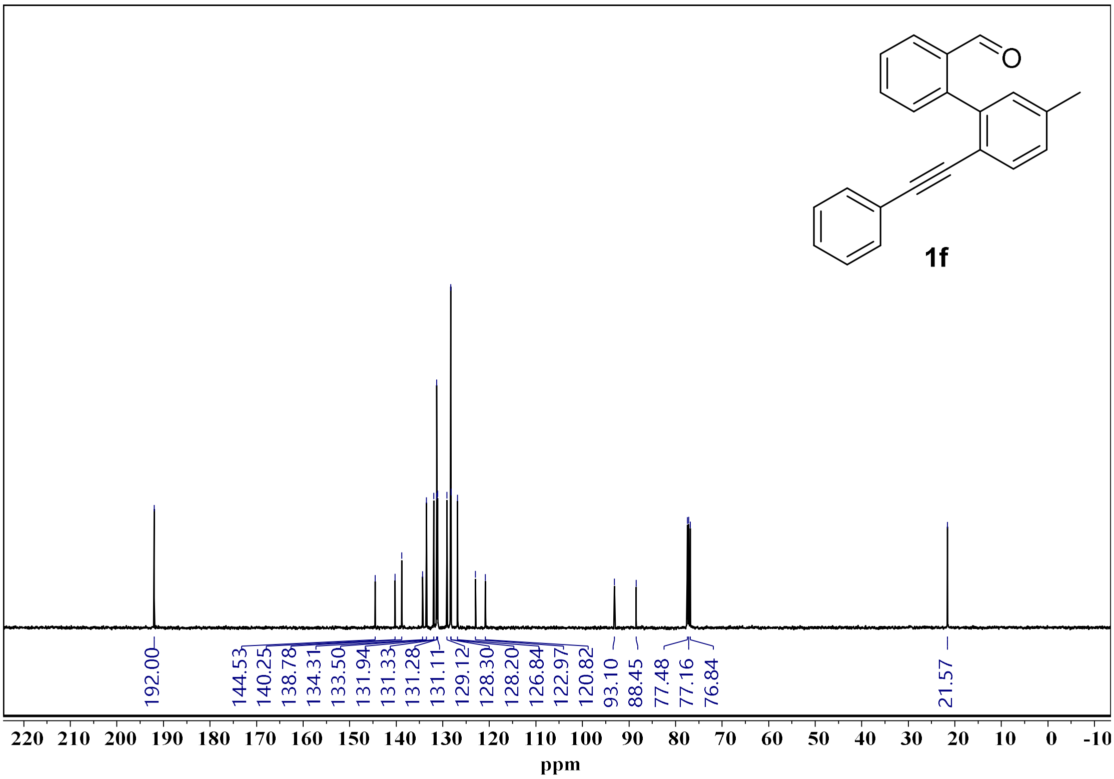

^{1}H NMR (400 MHz, $CDCl_3$) chart of **1g**

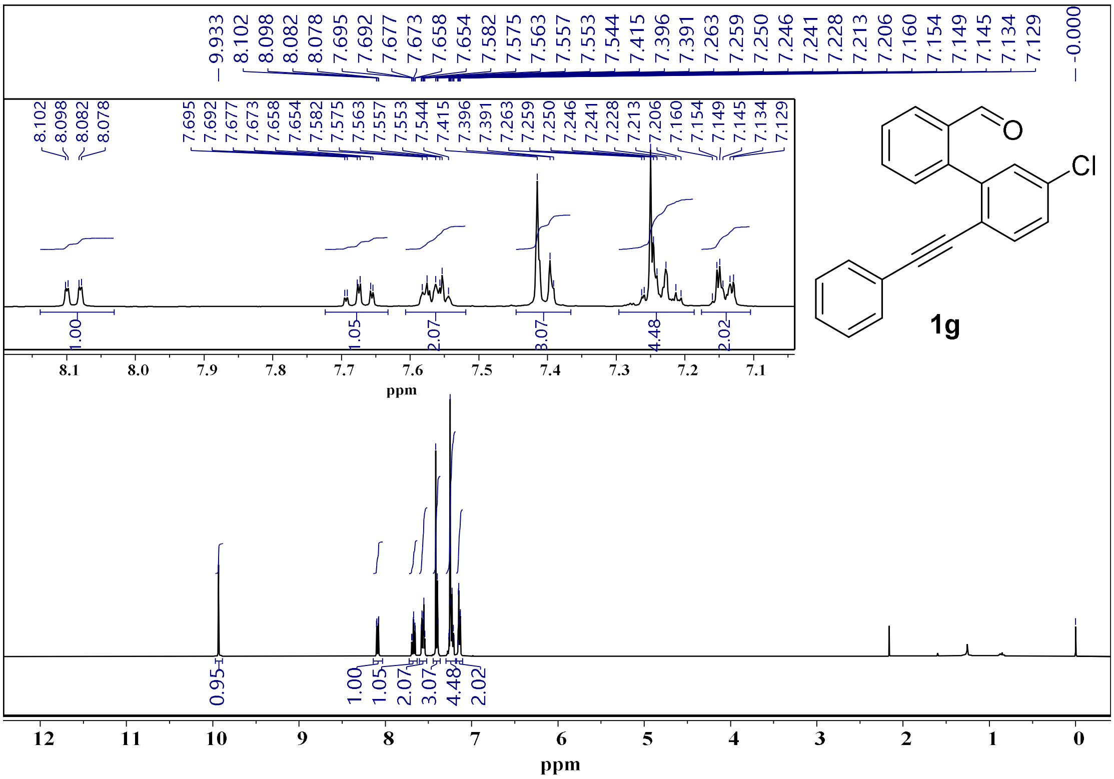

^{13}C{^{1}H} NMR (101 MHz, $CDCl_3$) chart of **1g**

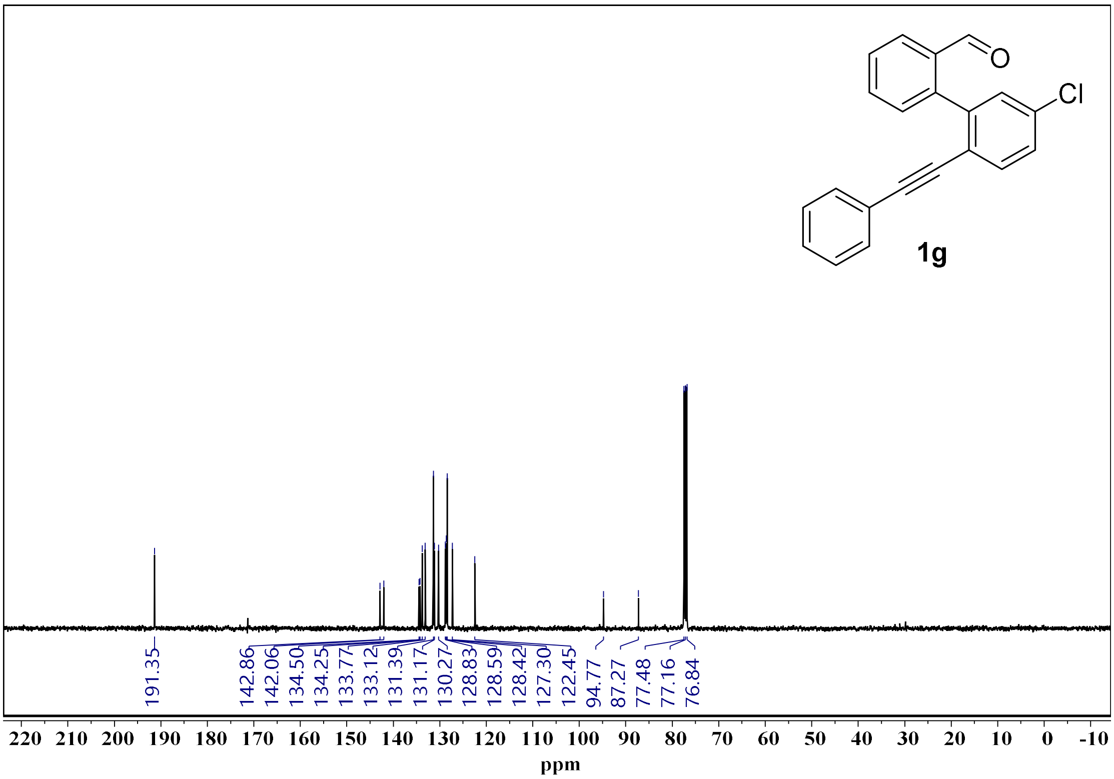

^{1}H NMR (400 MHz, $CDCl_3$) chart of **1h**

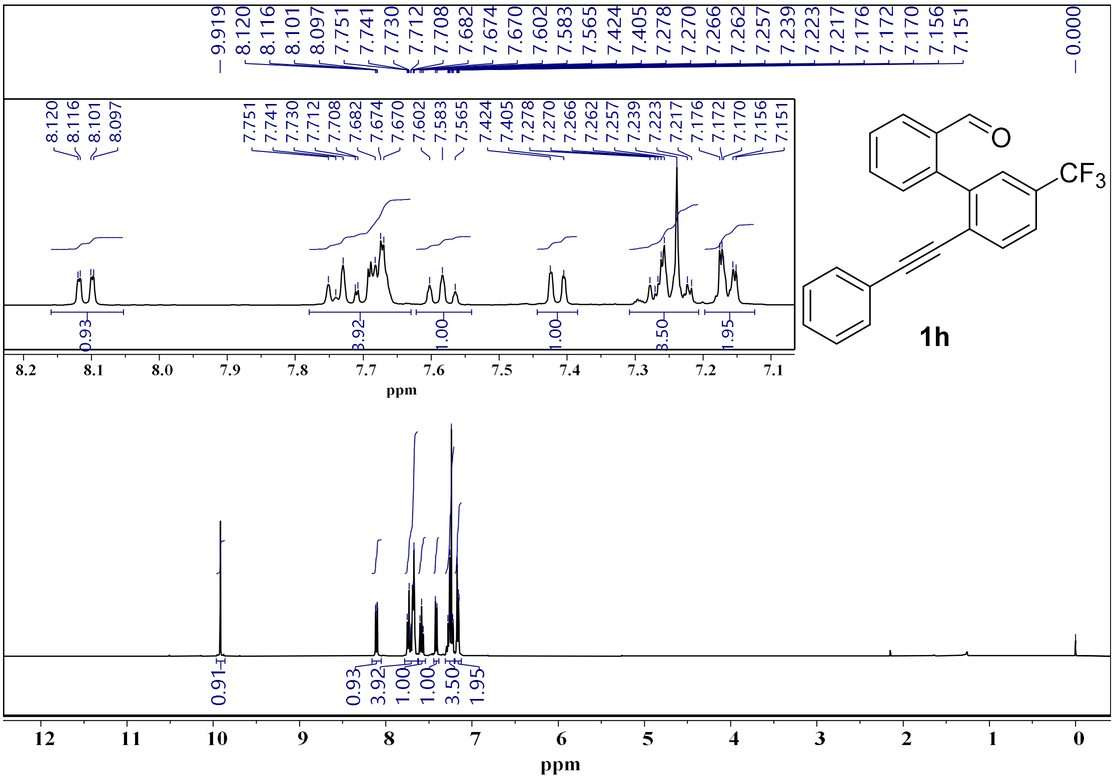

$^{13}C\{^{1}H\}$ NMR (101 MHz, $CDCl_3$) chart of **1h**

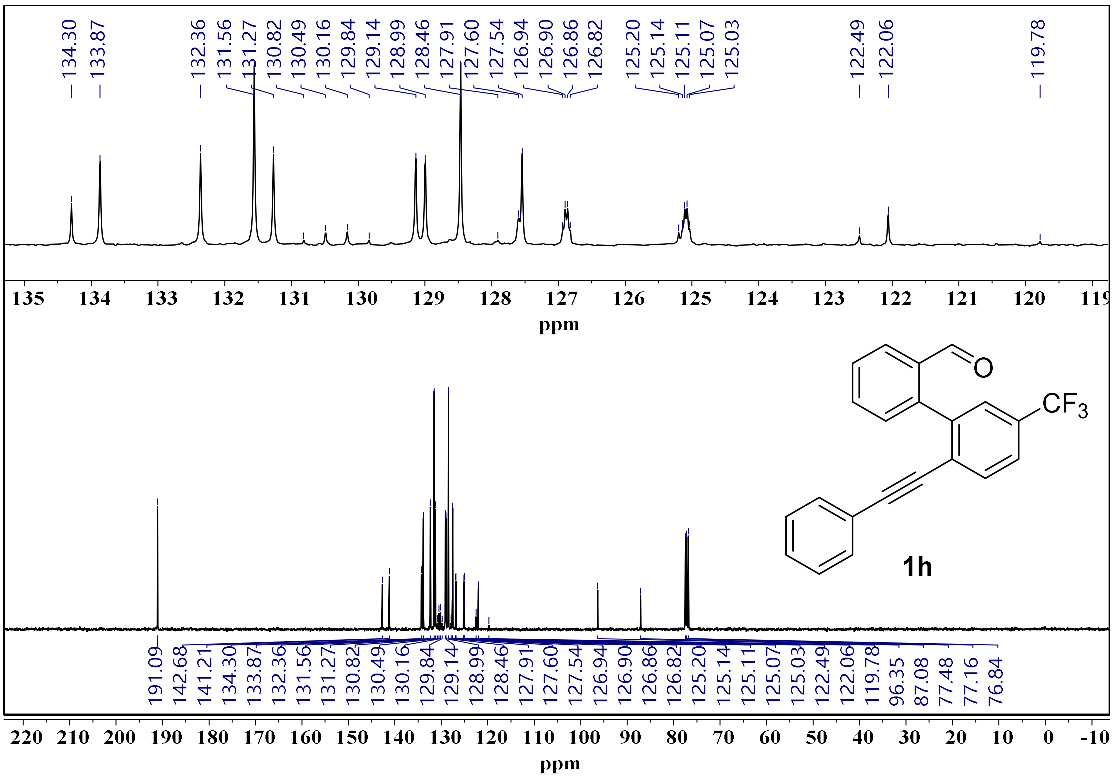

^{19}F NMR (376 MHz, $CDCl_3$) chart of **1h**

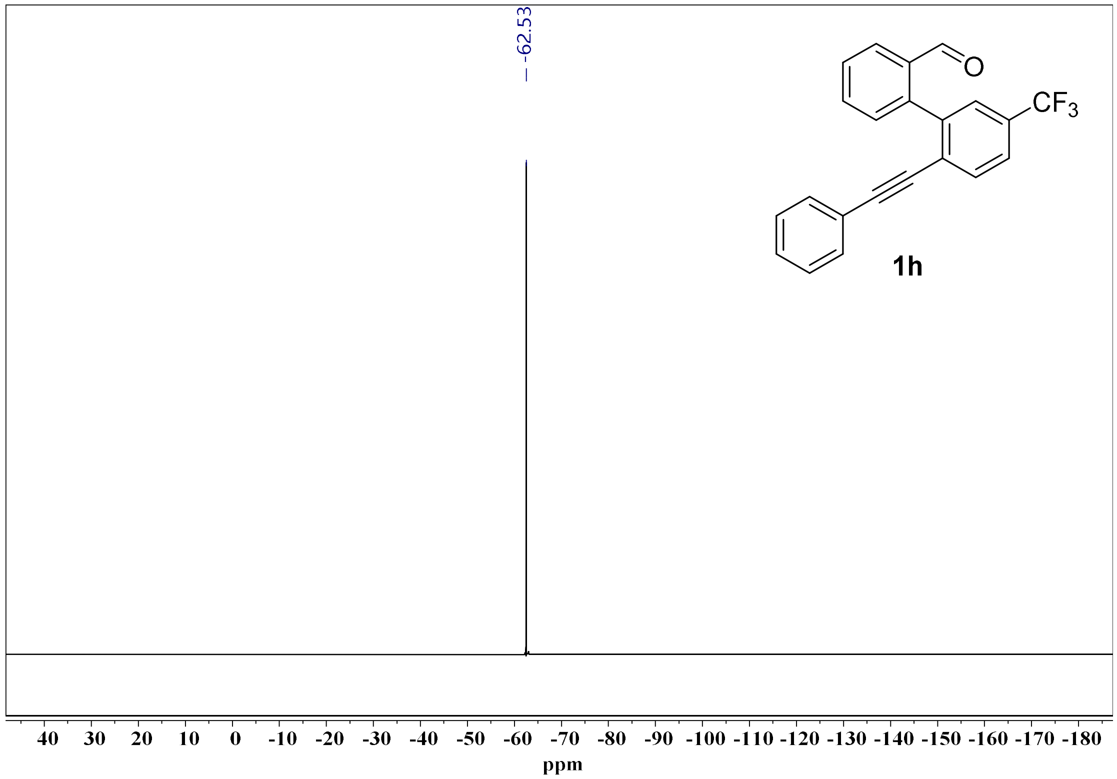

^{1}H NMR (400 MHz, $CDCl_3$) chart of **1i**

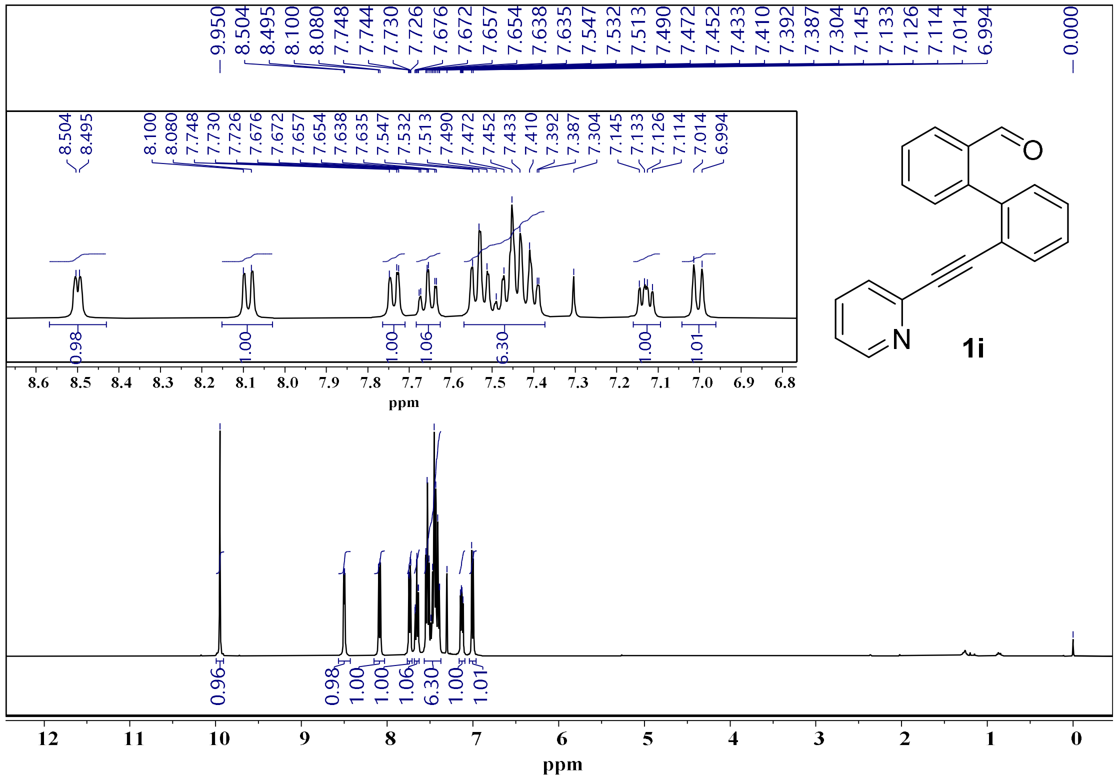

^{13}C{^{1}H} NMR (101 MHz, $CDCl_3$) chart of **1i**

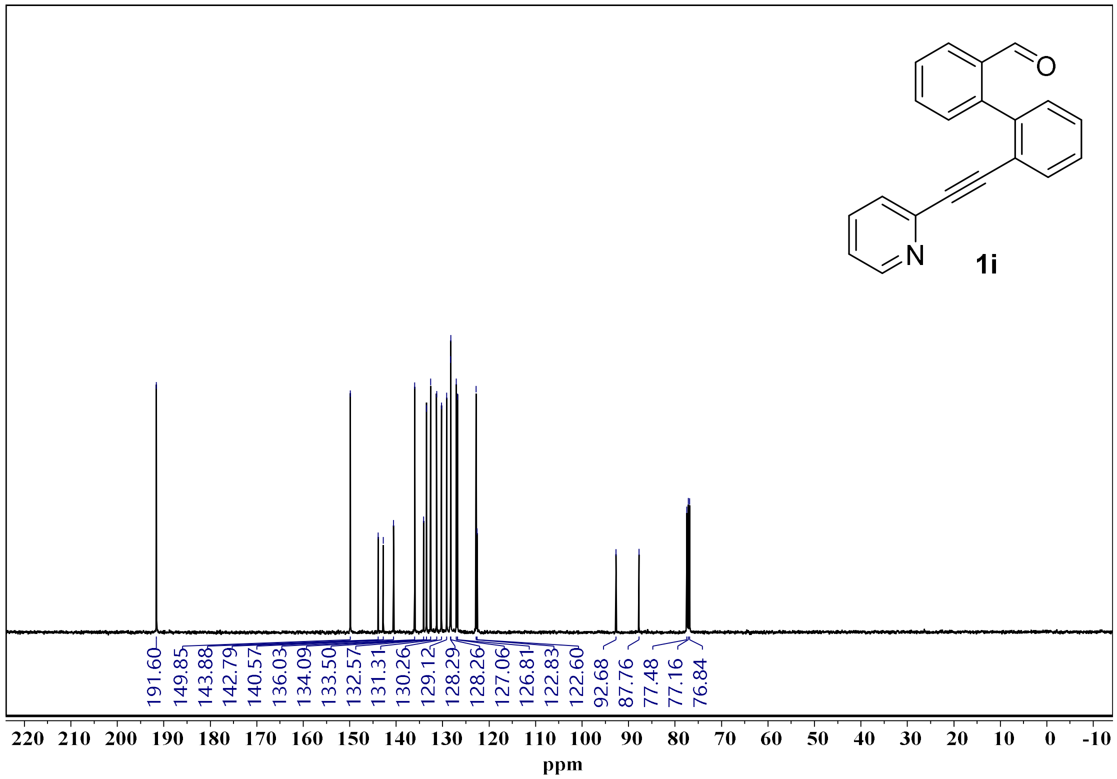

^{1}H NMR (400 MHz, $CDCl_3$) chart of **1j**

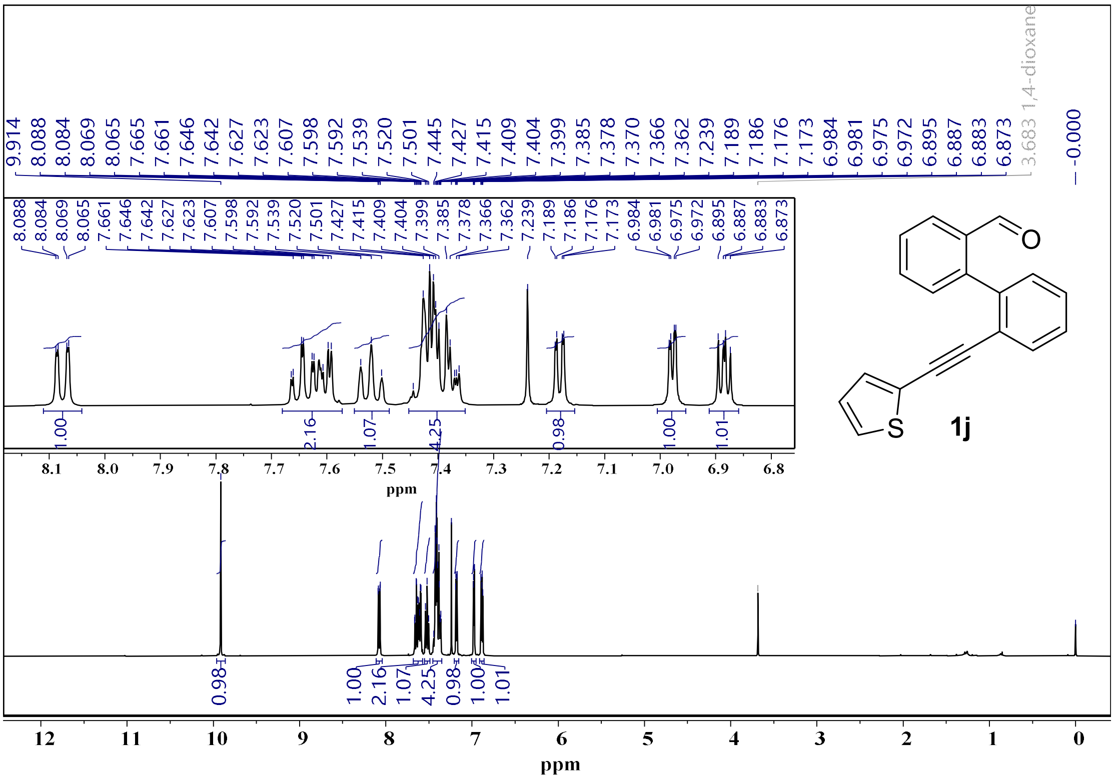

^{13}C{^{1}H} NMR (101 MHz, $CDCl_3$) chart of **1j**

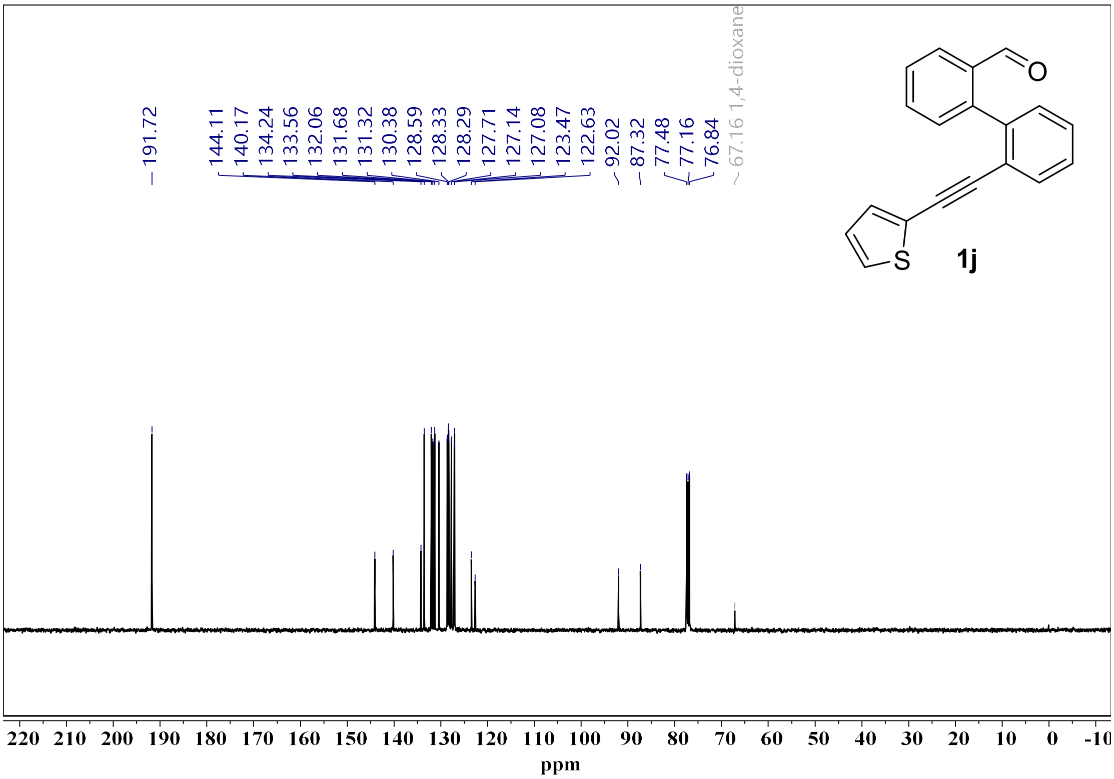

^{1}H NMR (400 MHz, $CDCl_3$) chart of **1k**

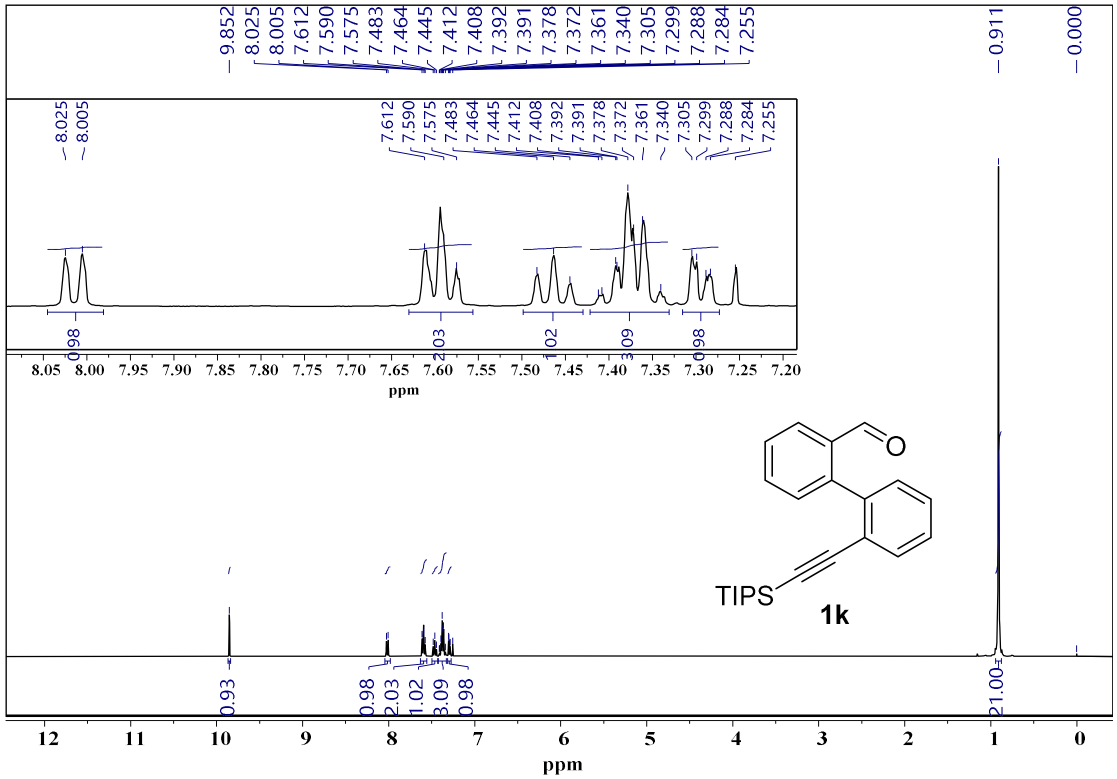

^{13}C{^{1}H} NMR (101 MHz, $CDCl_3$) chart of **1k**

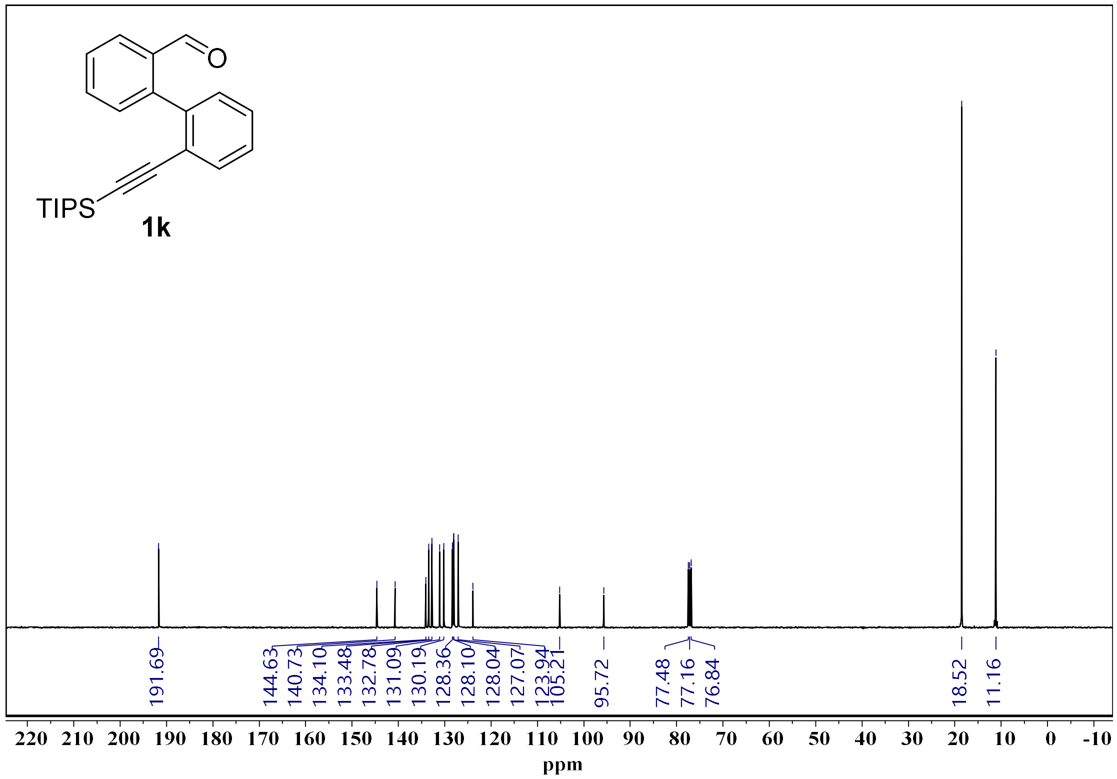

^{1}H NMR (400 MHz, $CDCl_3$) chart of **1l**

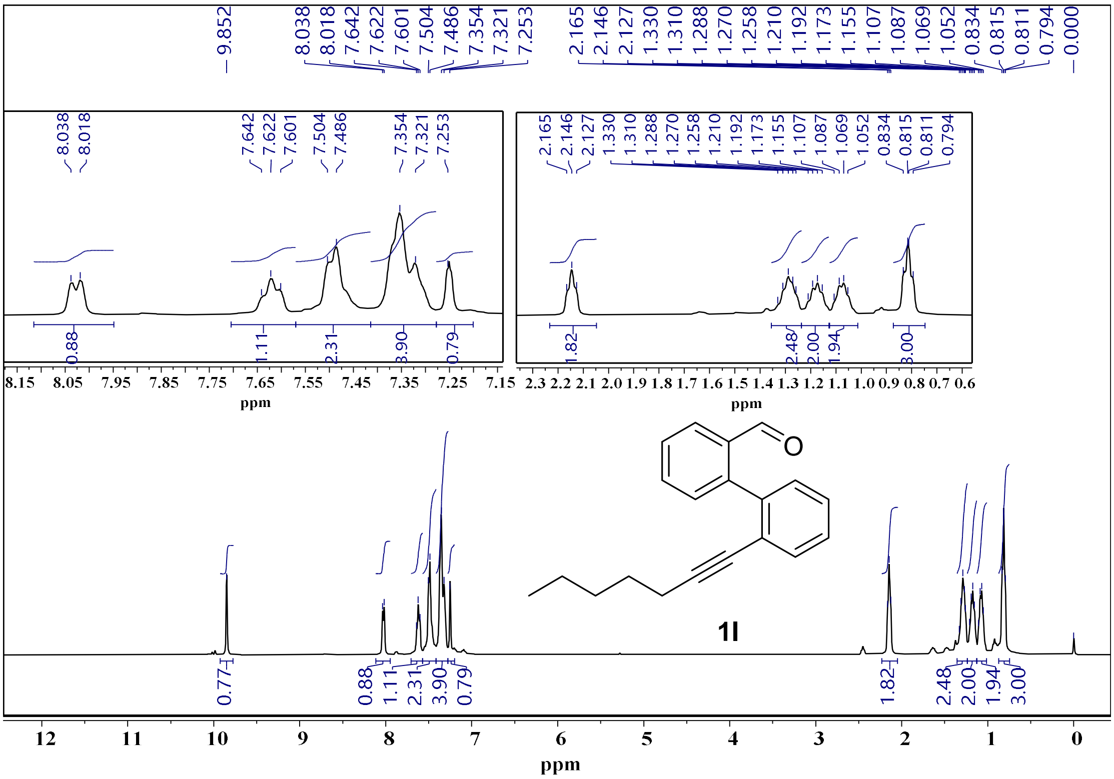

^{13}C{^{1}H} NMR (101 MHz, $CDCl_3$) chart of **1l**

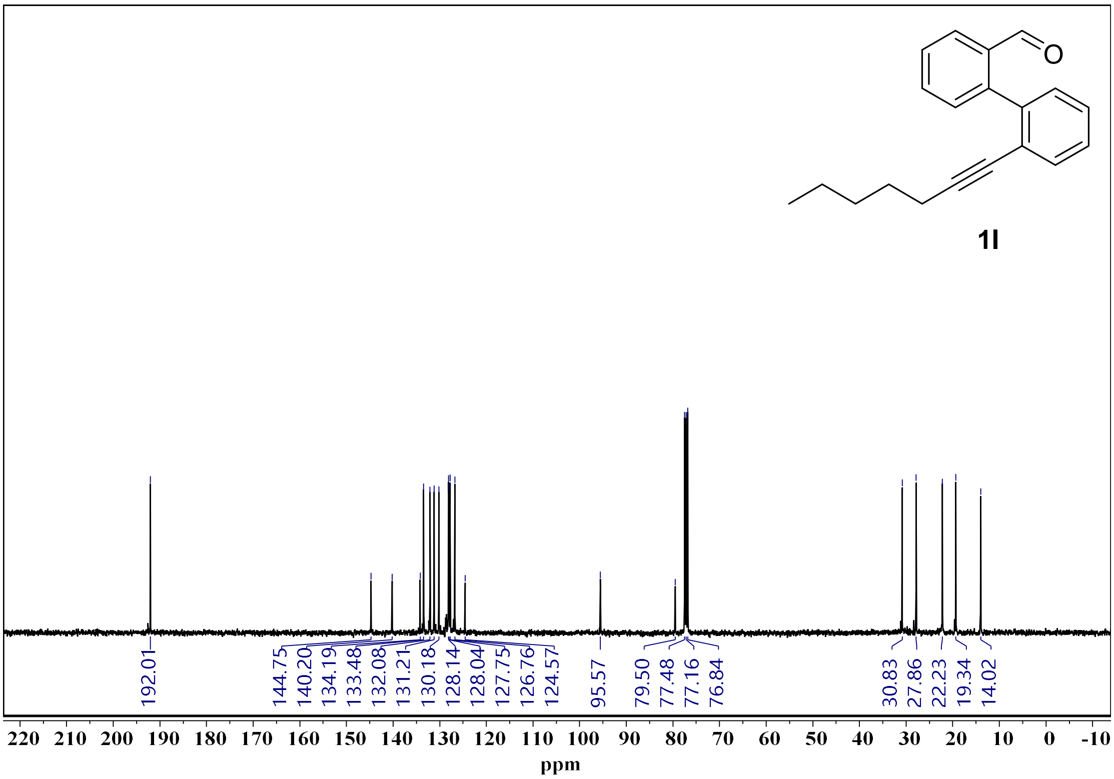

^1H NMR (400 MHz, $CDCl_3$) chart of **1m**

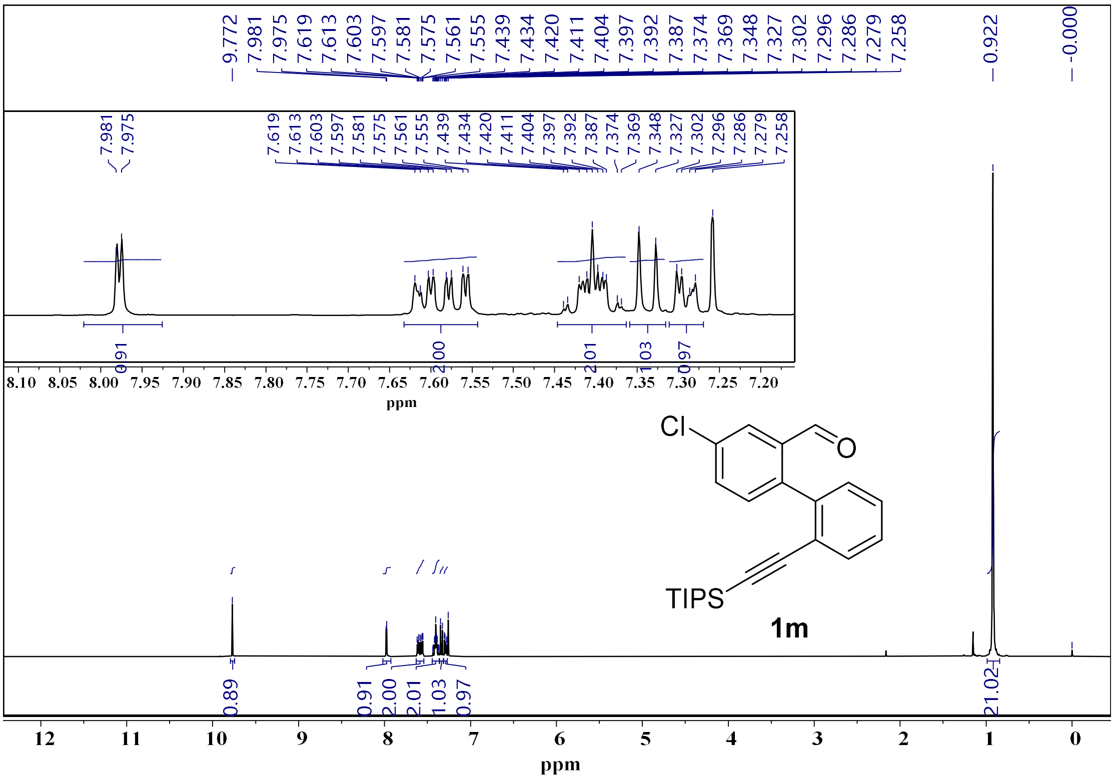

$^{13}C\{^1H\}$ NMR (101 MHz, $CDCl_3$) chart of **1m**

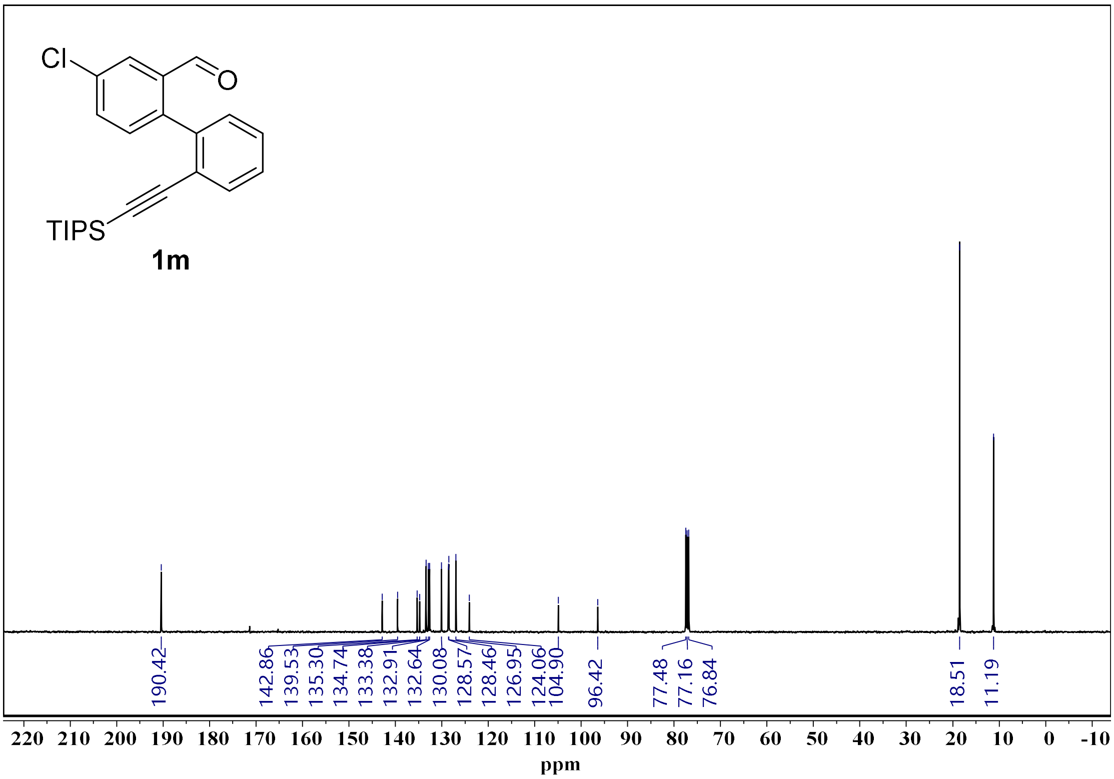

^{1}H NMR (400 MHz, $CDCl_3$) chart of **1n**

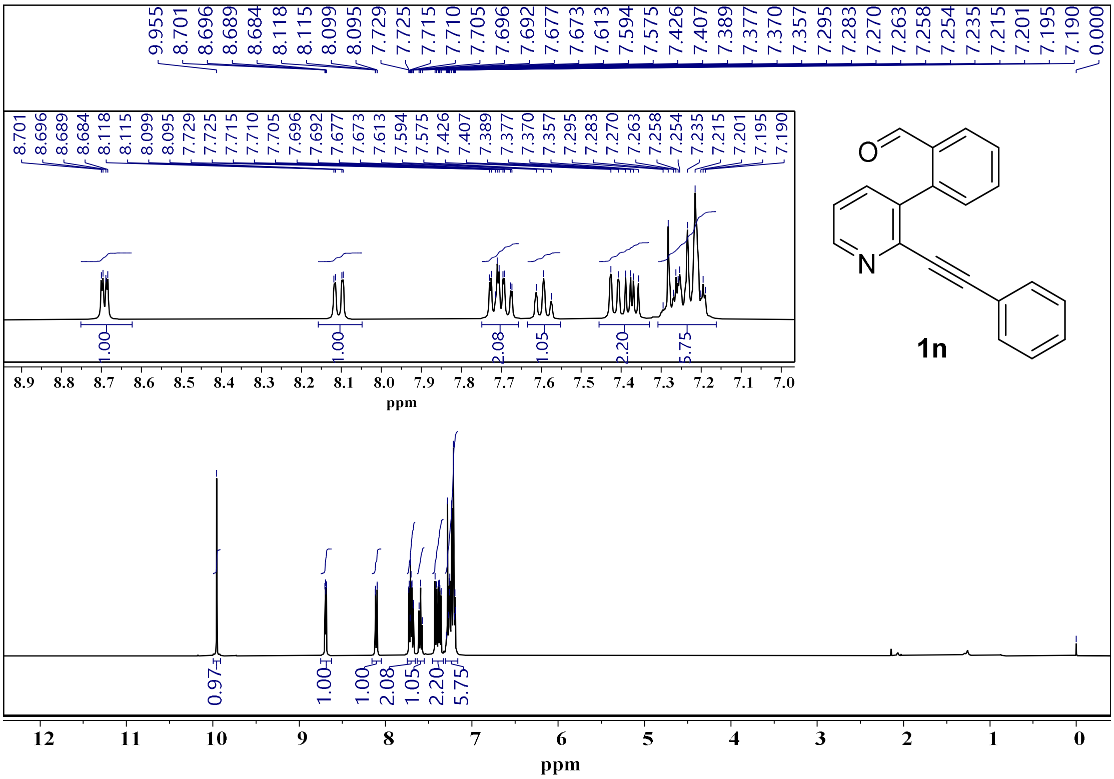

^{13}C{^{1}H} NMR (101 MHz, $CDCl_3$) chart of **1n**

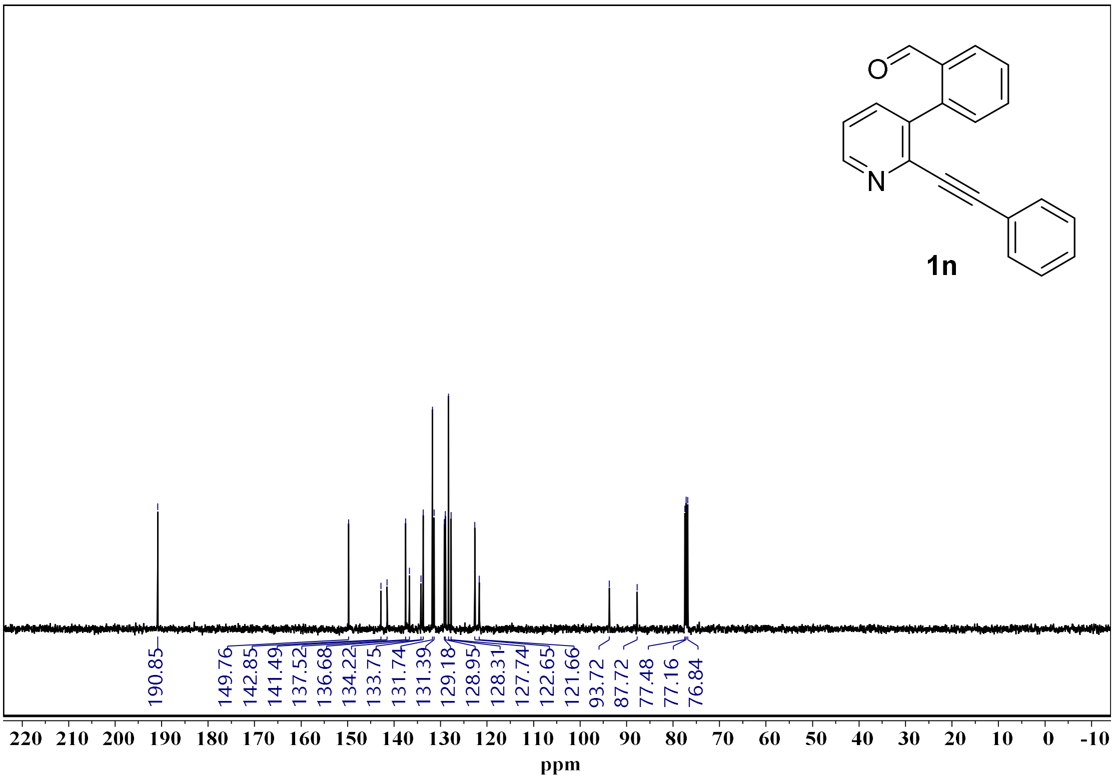

^{1}H NMR (400 MHz, $CDCl_3$) chart of **1o**

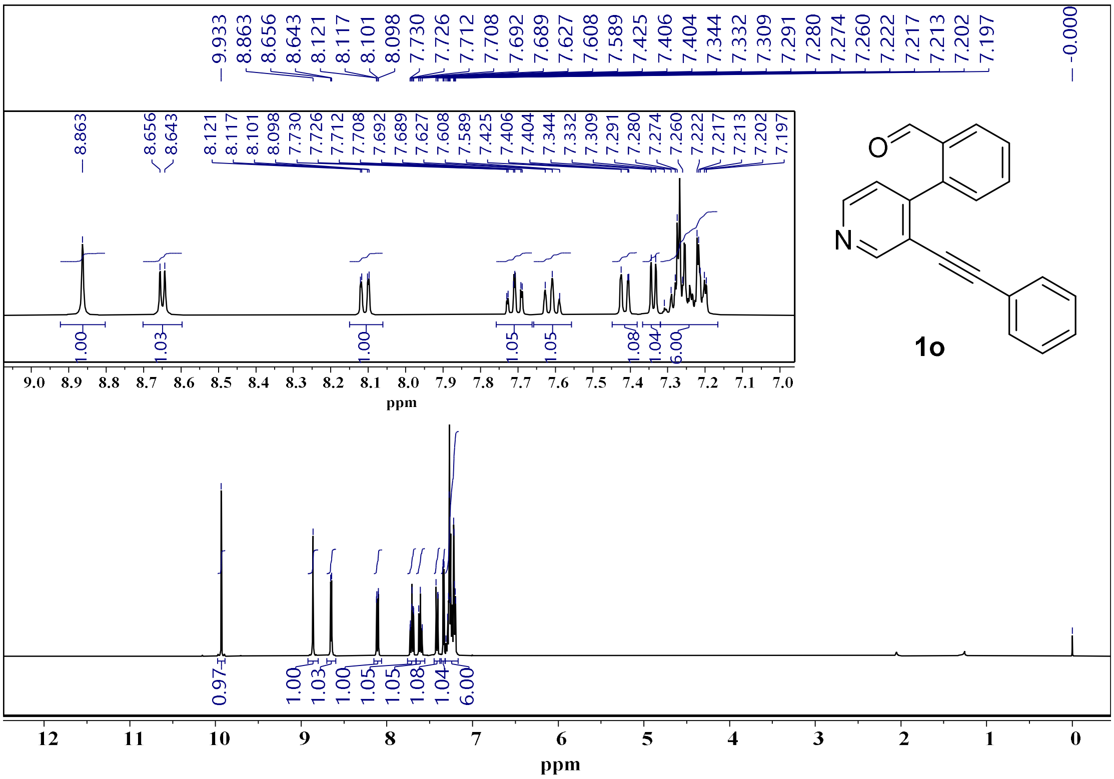

$^{13}C\{^{1}H\}$ NMR (101 MHz, $CDCl_3$) chart of **1o**

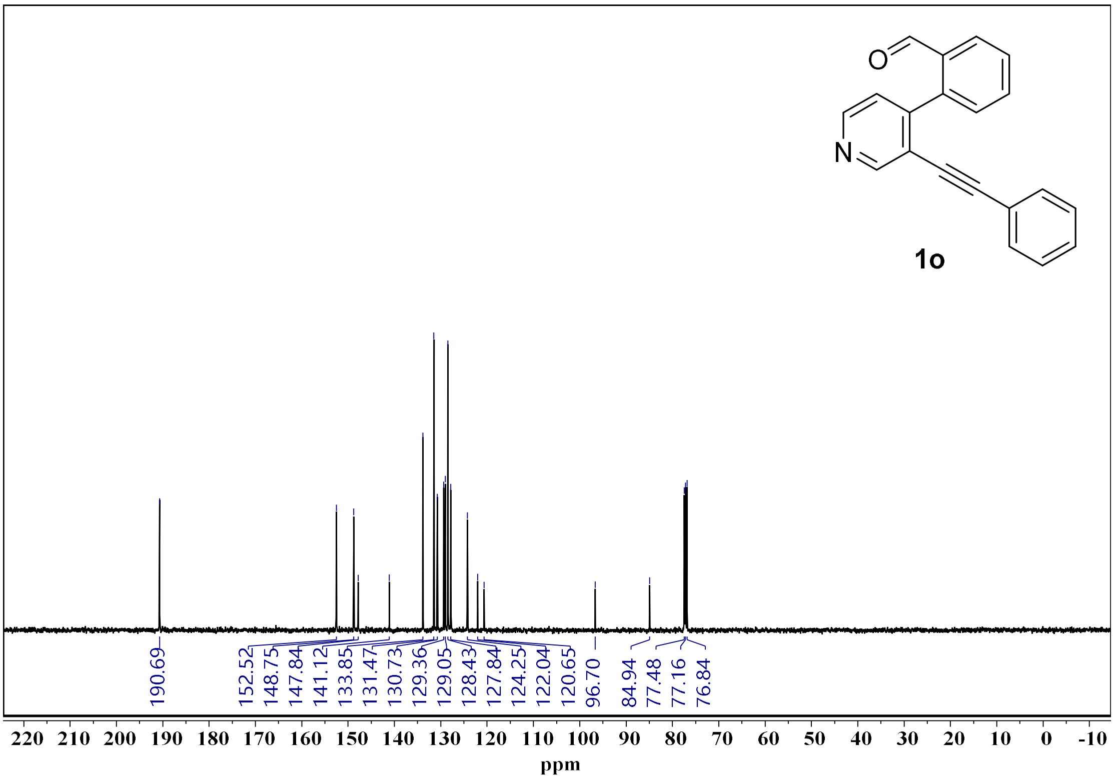

^{1}H NMR (400 MHz, $CDCl_3$) chart of **1p**

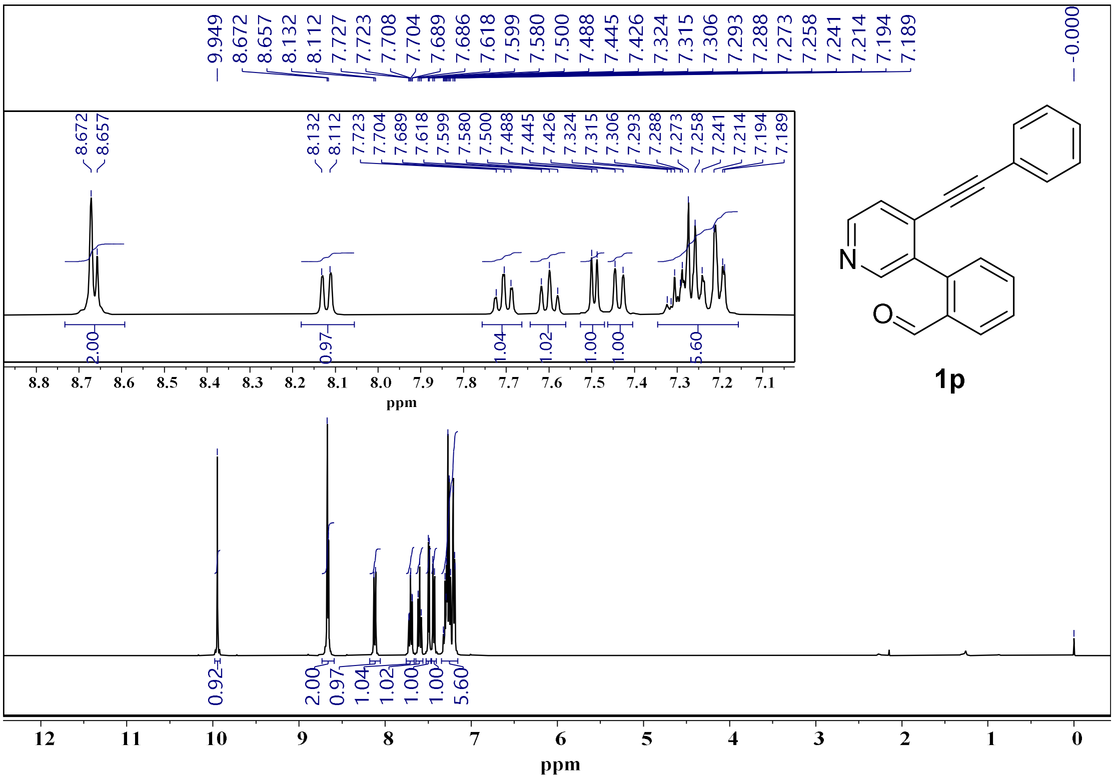

^{13}C{^{1}H} NMR (101 MHz, $CDCl_3$) chart of **1p**

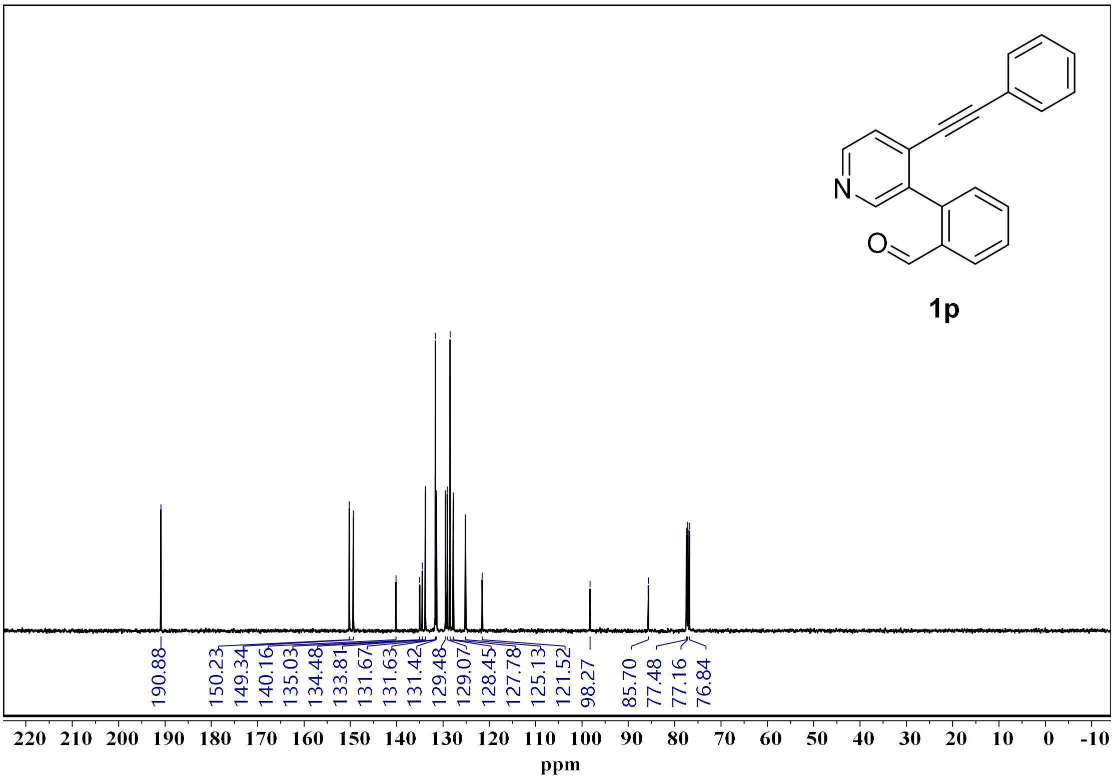

^{1}H NMR (400 MHz, $CDCl_3$) chart of **1a'**

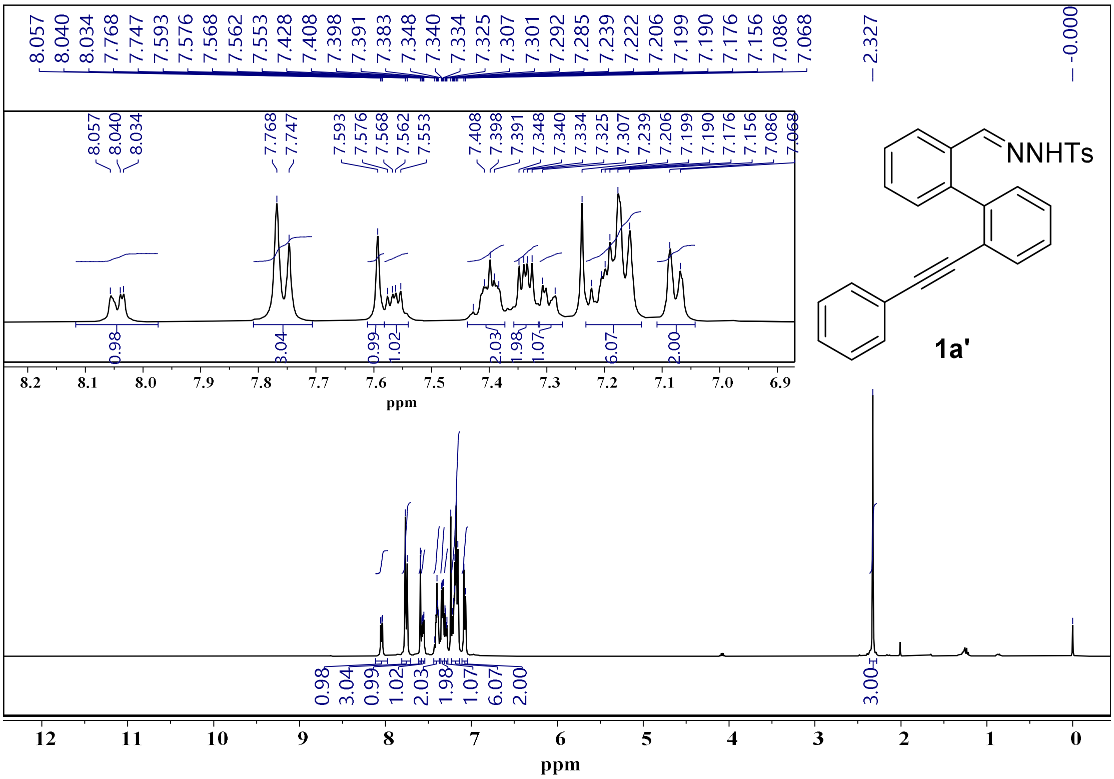

^{13}C{^{1}H} NMR (101 MHz, $CDCl_3$) chart of **1a'**

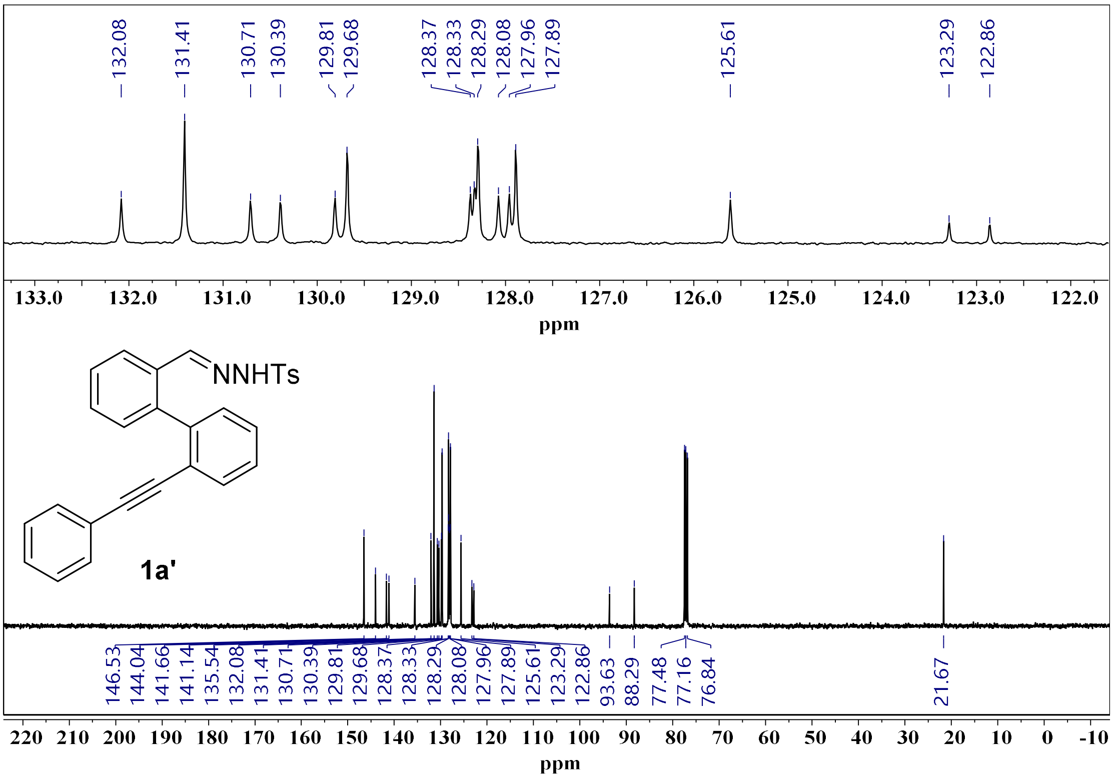

^{1}H NMR (400 MHz, DMSO-d_6) chart of **2a**

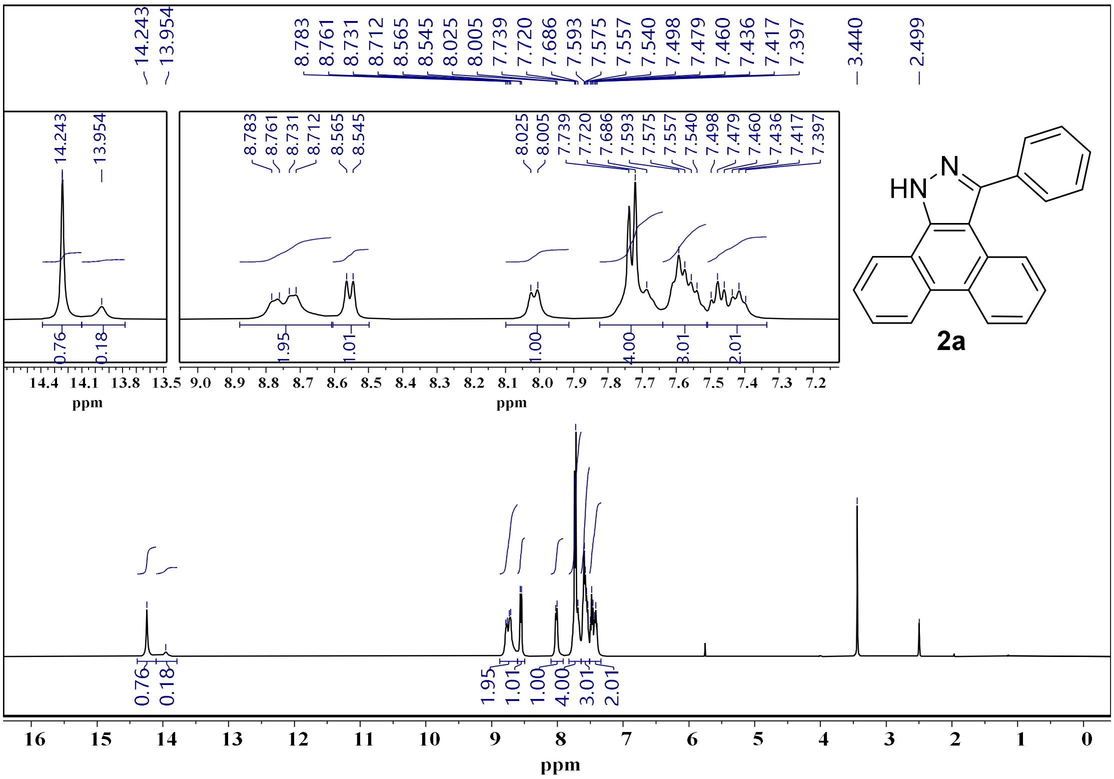

^{13}C{^{1}H} NMR (101 MHz, DMSO-d_6) chart of **2a**

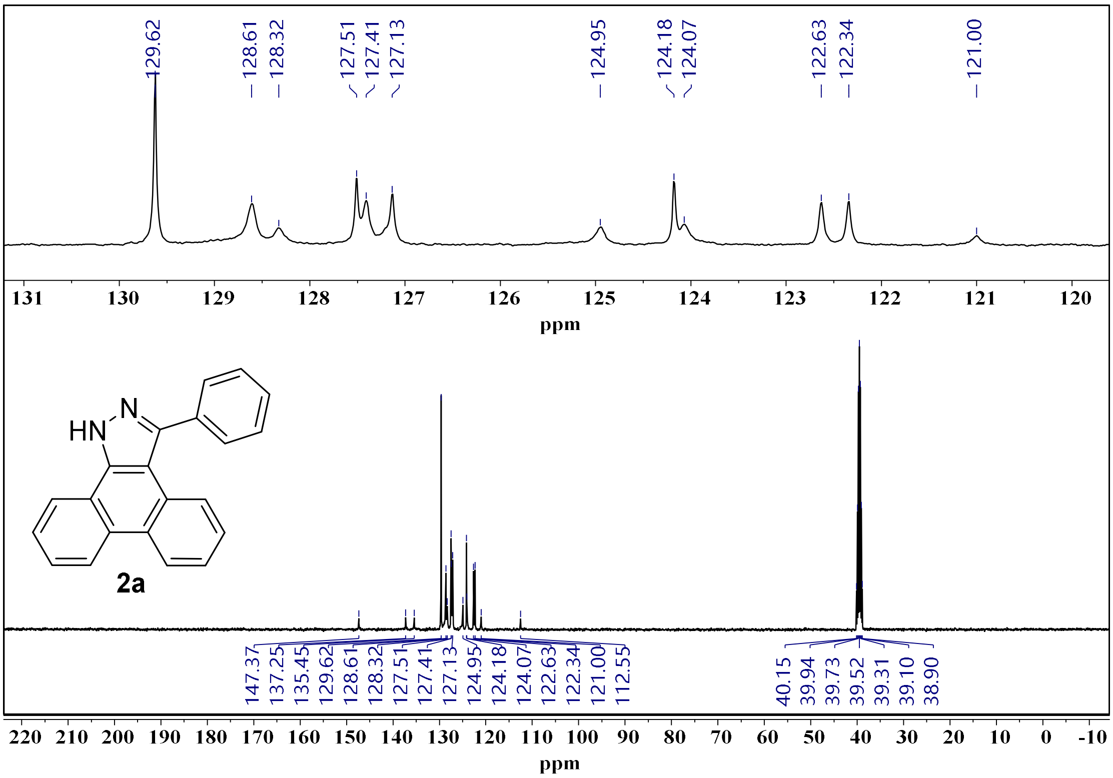

^{1}H NMR (400 MHz, DMSO-d_6) chart of **2b**

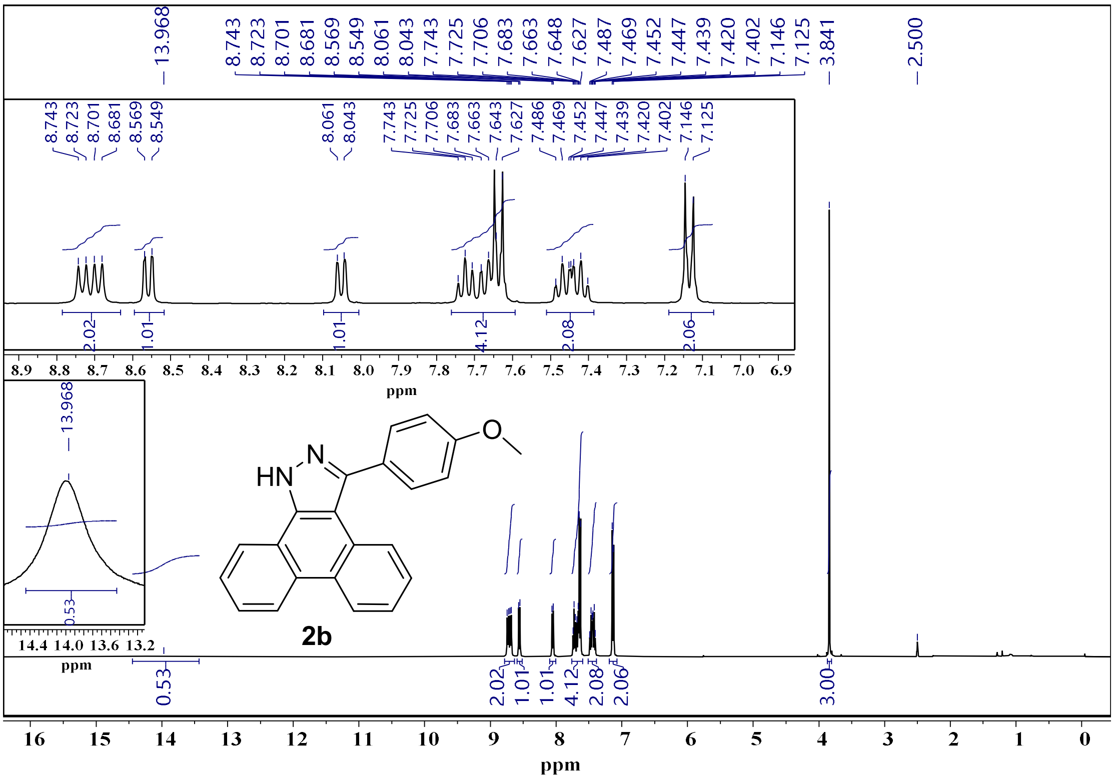

^{13}C{^{1}H} NMR (101 MHz, DMSO-d_6) chart of **2b**

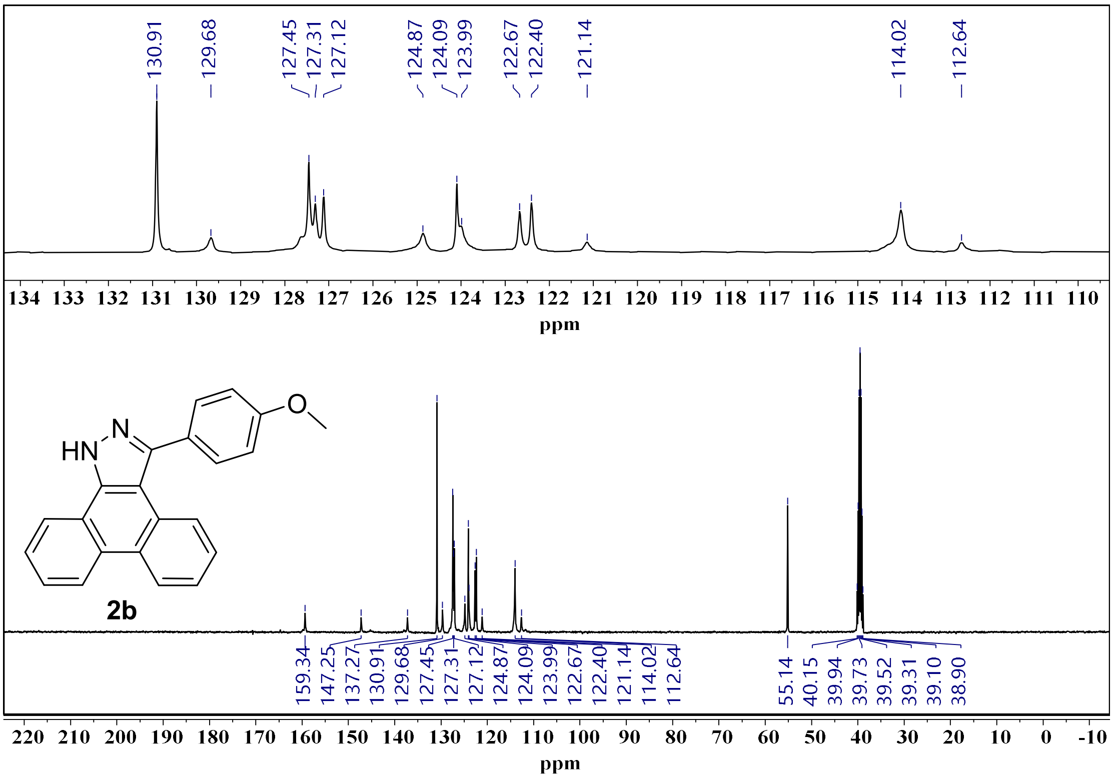

^{1}H NMR (400 MHz, DMSO-d_6) chart of **2c**

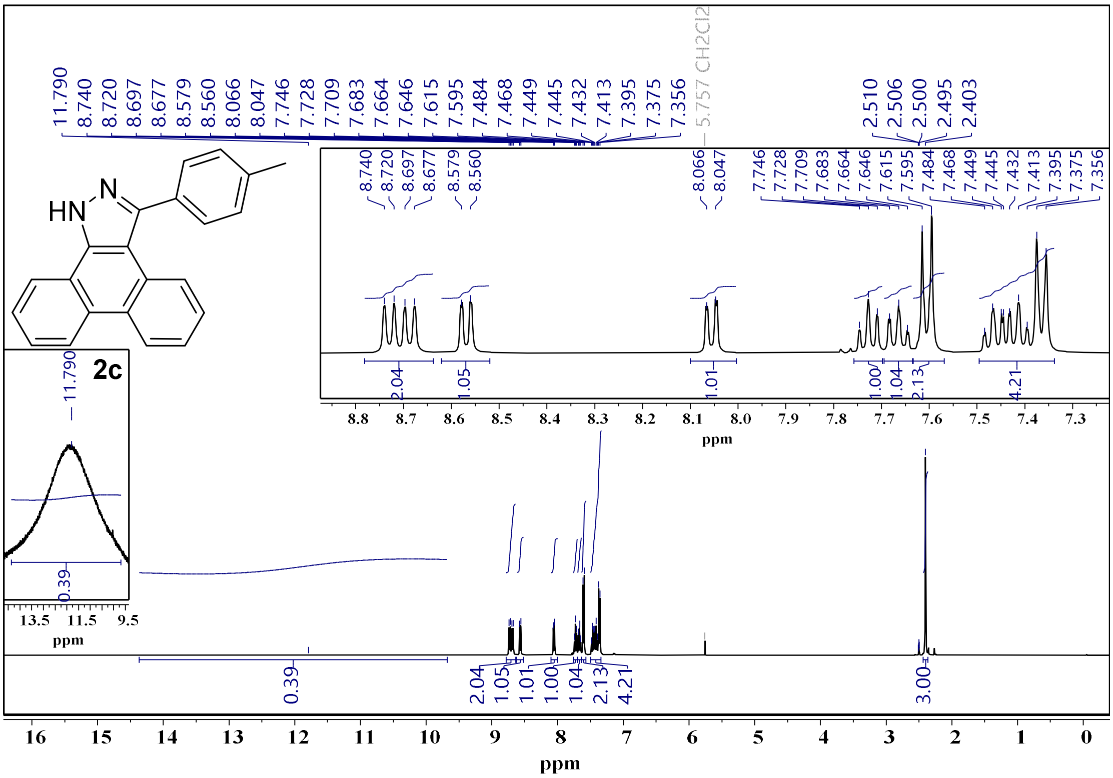

^{13}C{^{1}H} NMR (101 MHz, DMSO-d_6) chart of **2c**

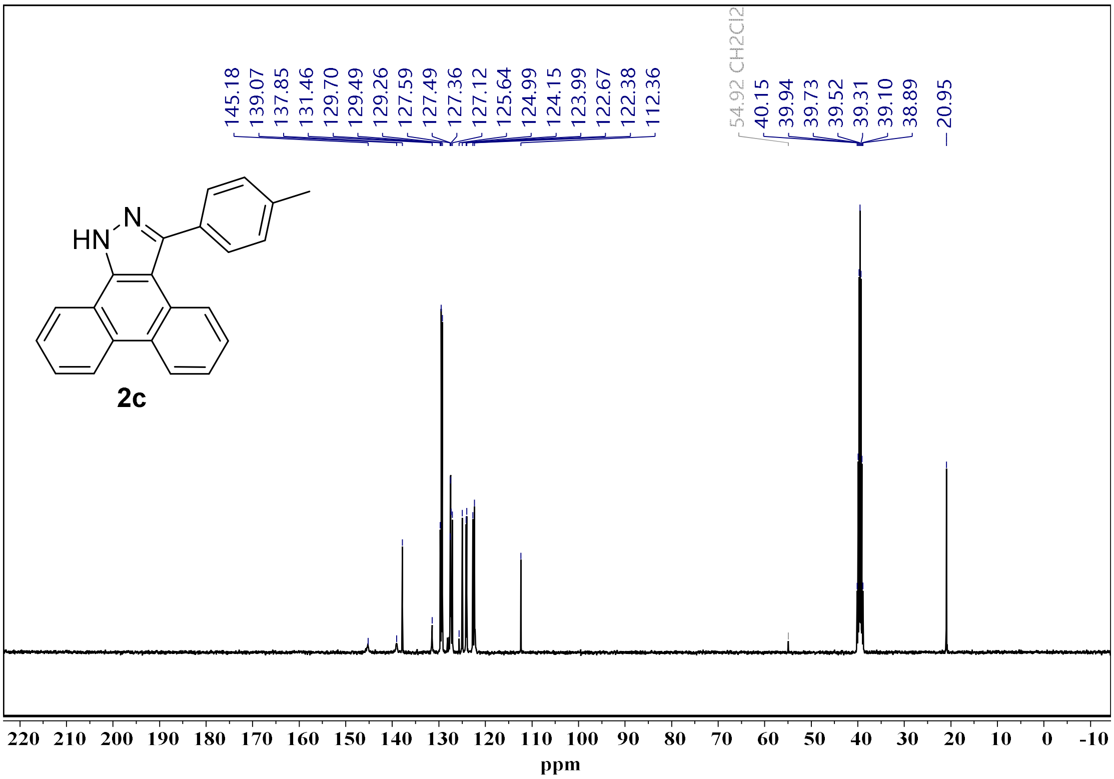

^{1}H NMR (400 MHz, DMSO-d_6) chart of **2d**

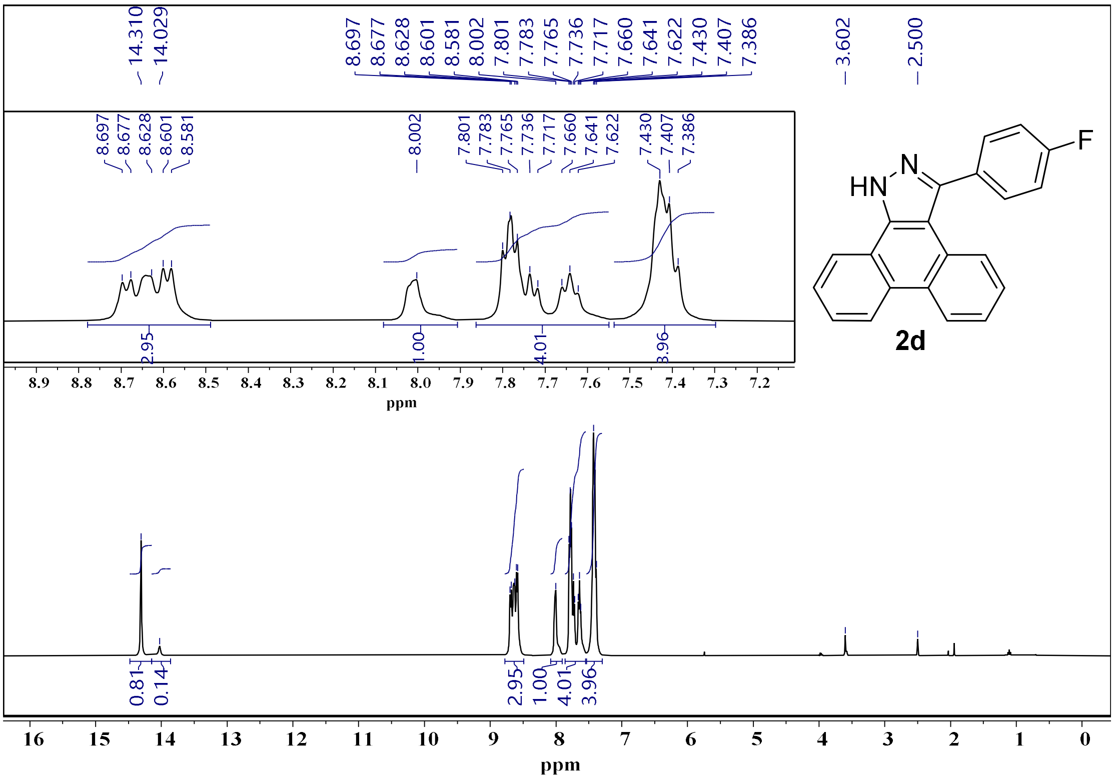

^{13}C{^{1}H} NMR (101 MHz, DMSO-d_6) chart of **2d**

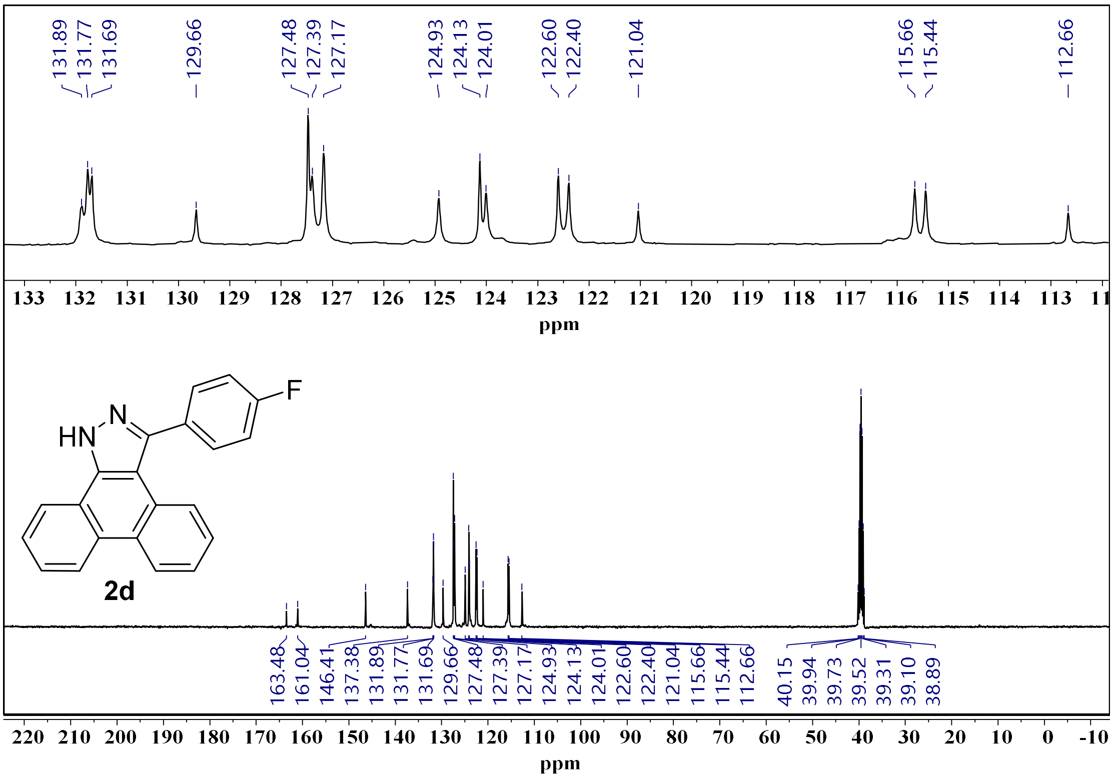

^{1}H NMR (400 MHz, DMSO-d_6) chart of **2e**

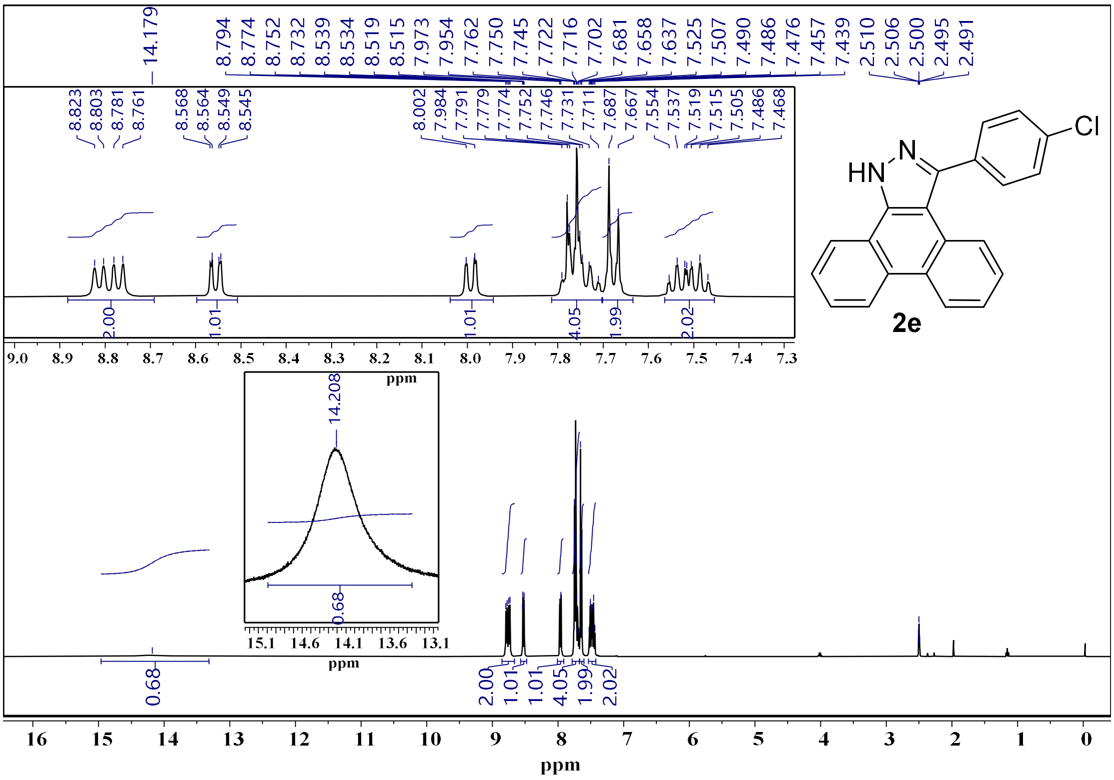

^{13}C{^{1}H} NMR (101 MHz, DMSO-d_6) chart of **2e**

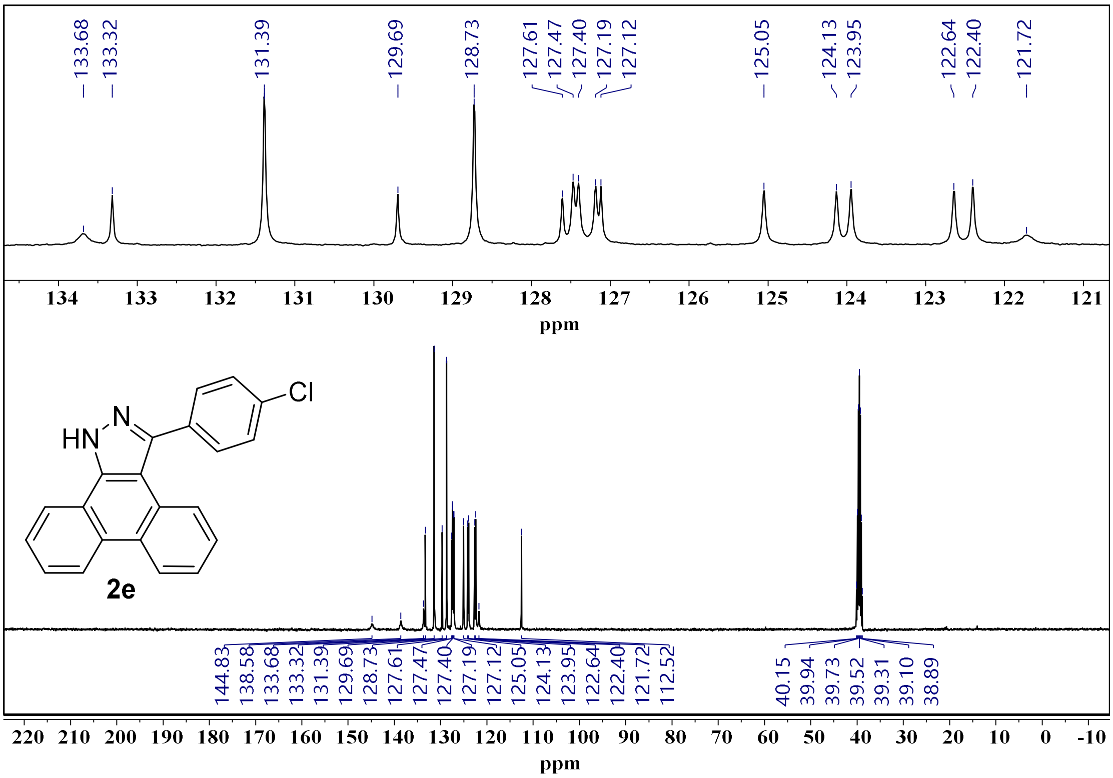

^{1}H NMR (400 MHz, DMSO-d_6) chart of **2f**

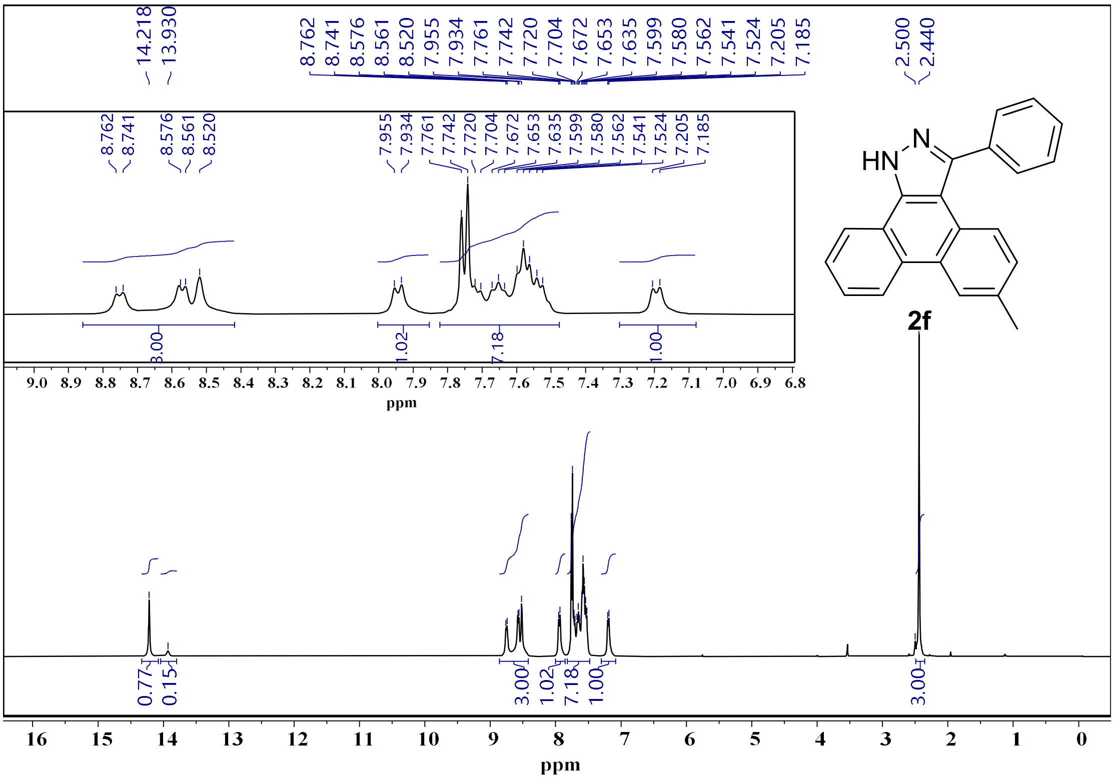

$^{13}C\{^{1}H\}$ NMR (101 MHz, DMSO-d_6) chart of **2f**

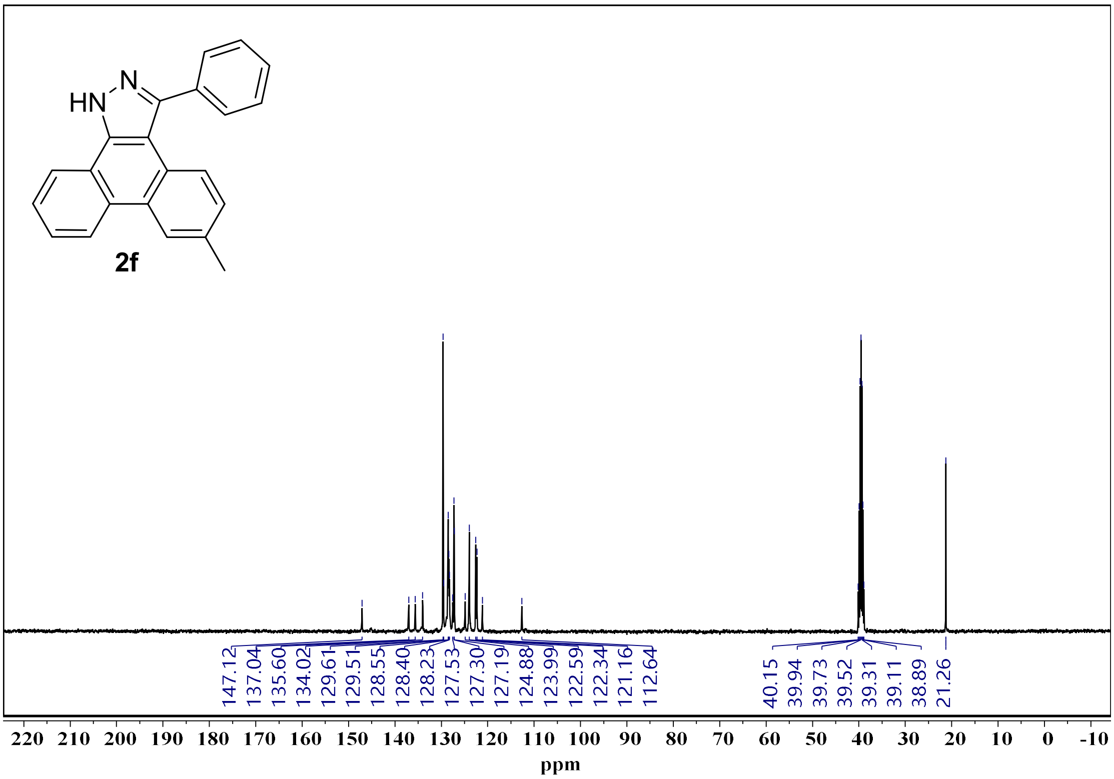

^{1}H NMR (400 MHz, DMSO-d_6) chart of **2g**

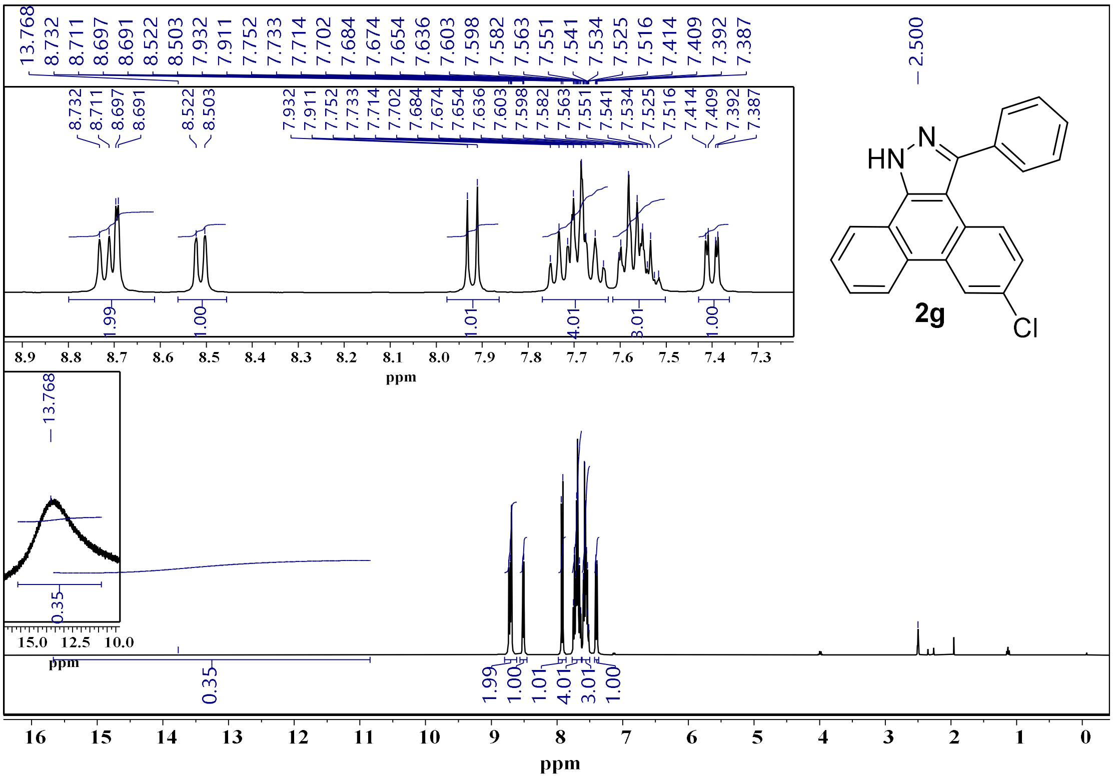

^{13}C{^{1}H} NMR (101 MHz, DMSO-d_6) chart of **2g**

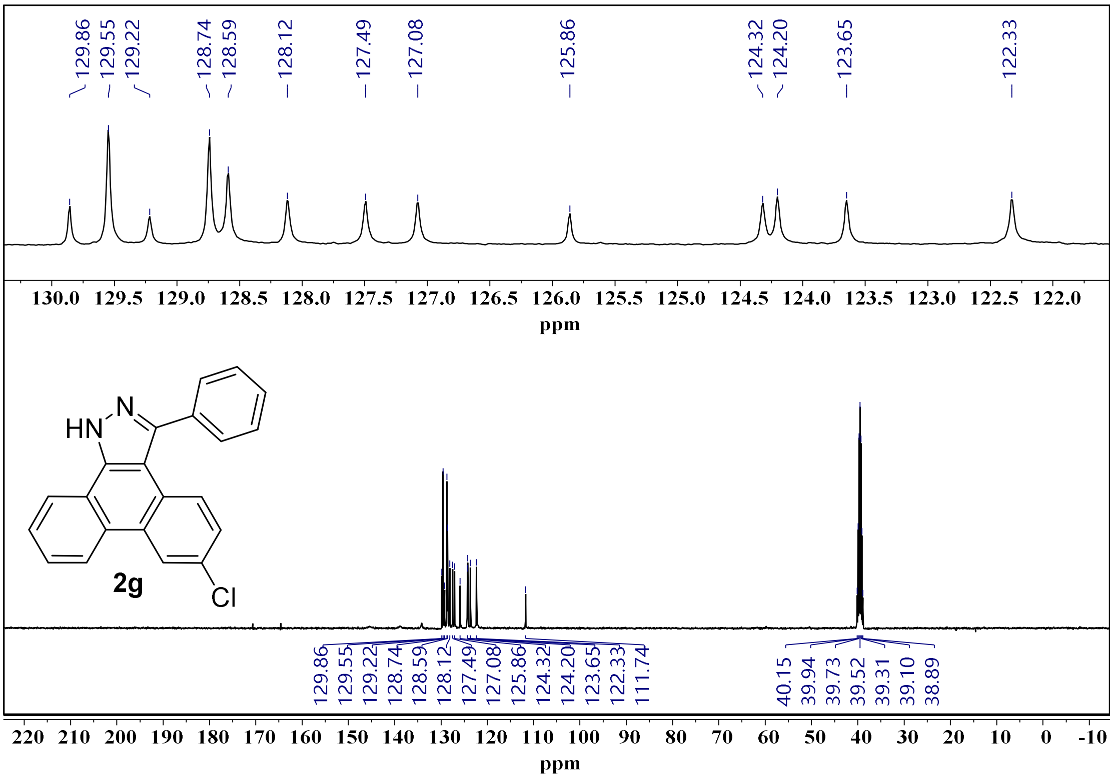

^{1}H NMR (400 MHz, DMSO-d_6) chart of **2h**

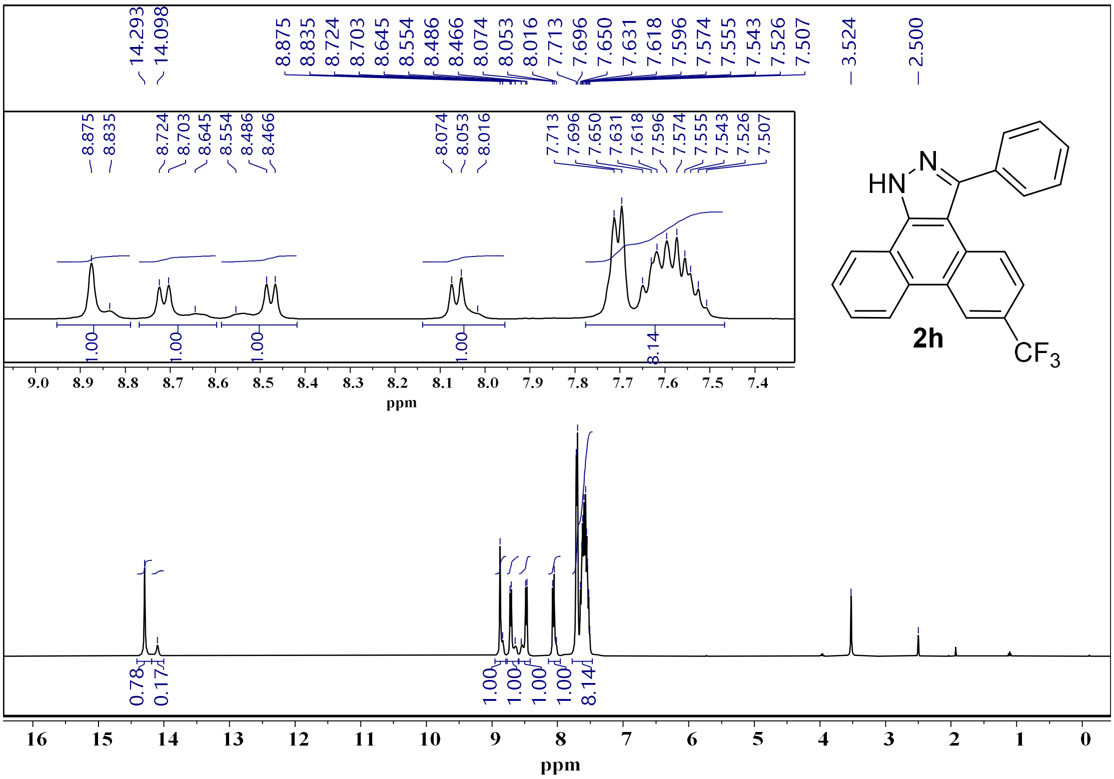

^{13}C{^{1}H} NMR (101 MHz, DMSO-d_6) chart of **2h**

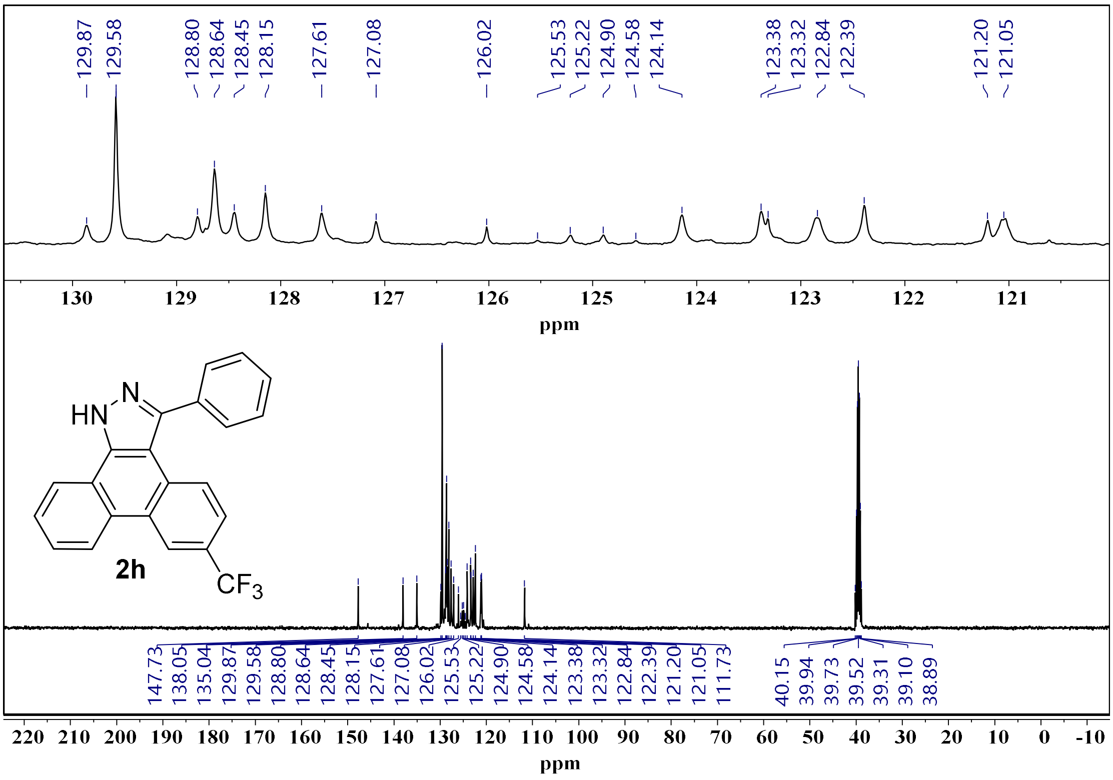

^{19}F NMR (376 MHz, $CDCl_3$) chart of **2h**

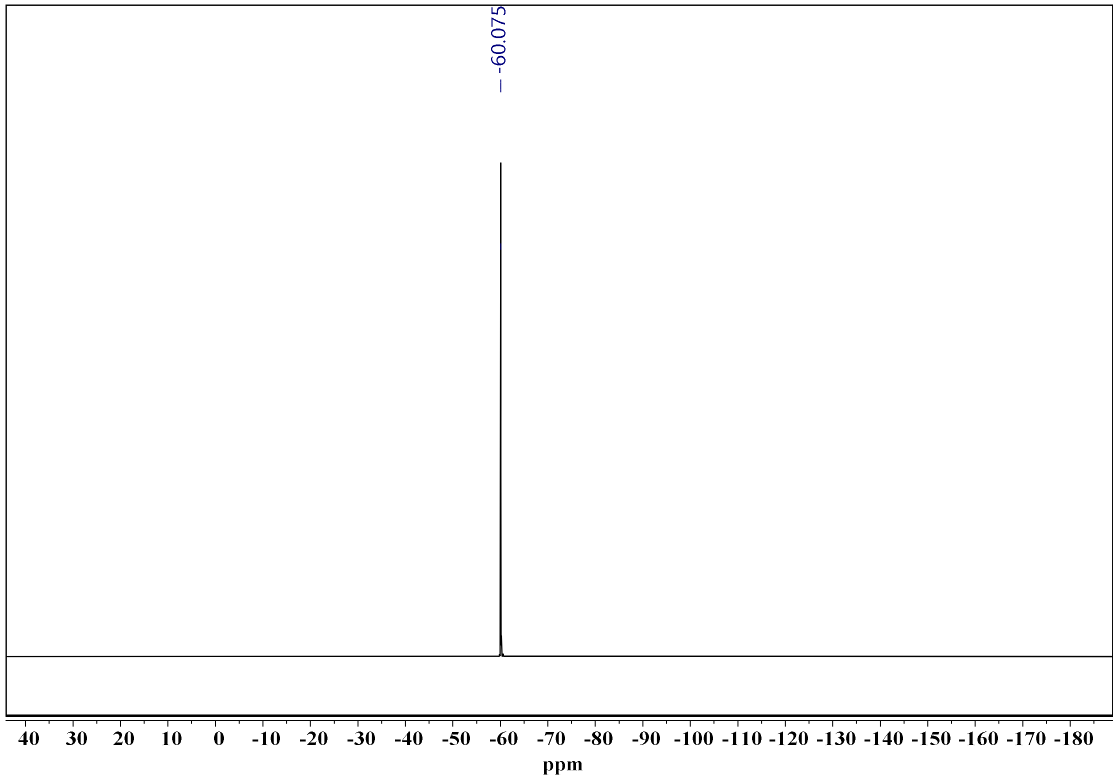

^{1}H NMR (400 MHz, DMSO-d_6) chart of **2i**

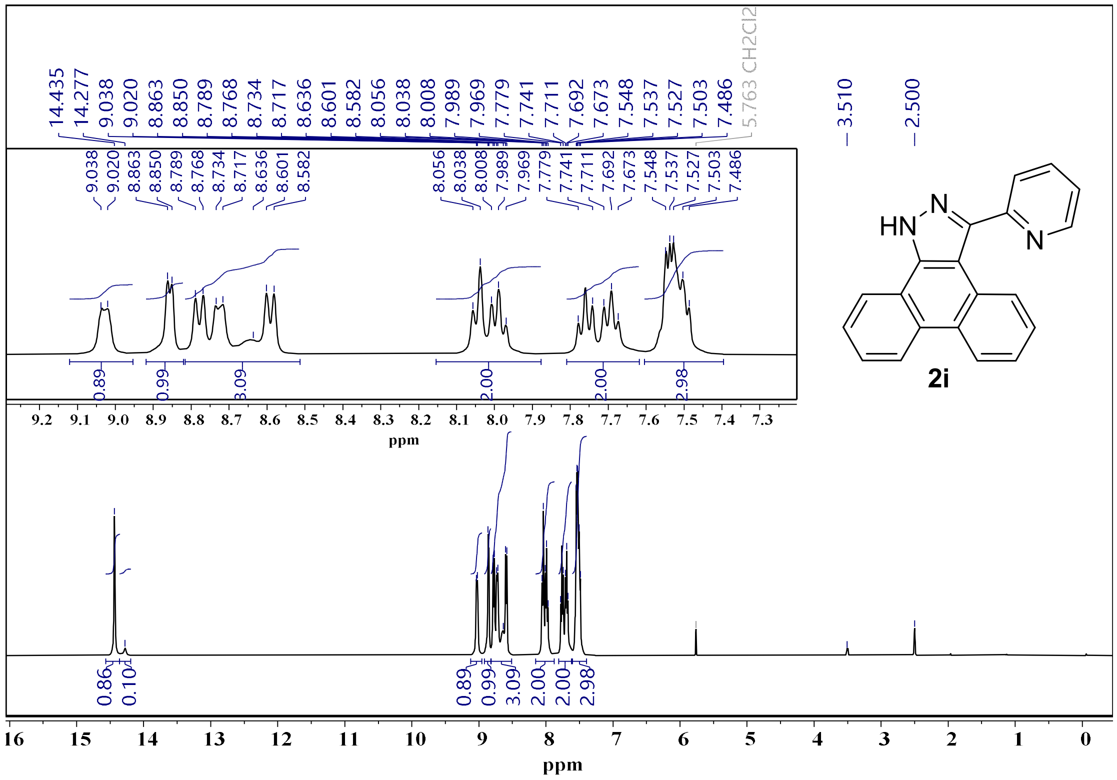

^{13}C{^{1}H} NMR (101 MHz, DMSO-d_6) chart of **2i**

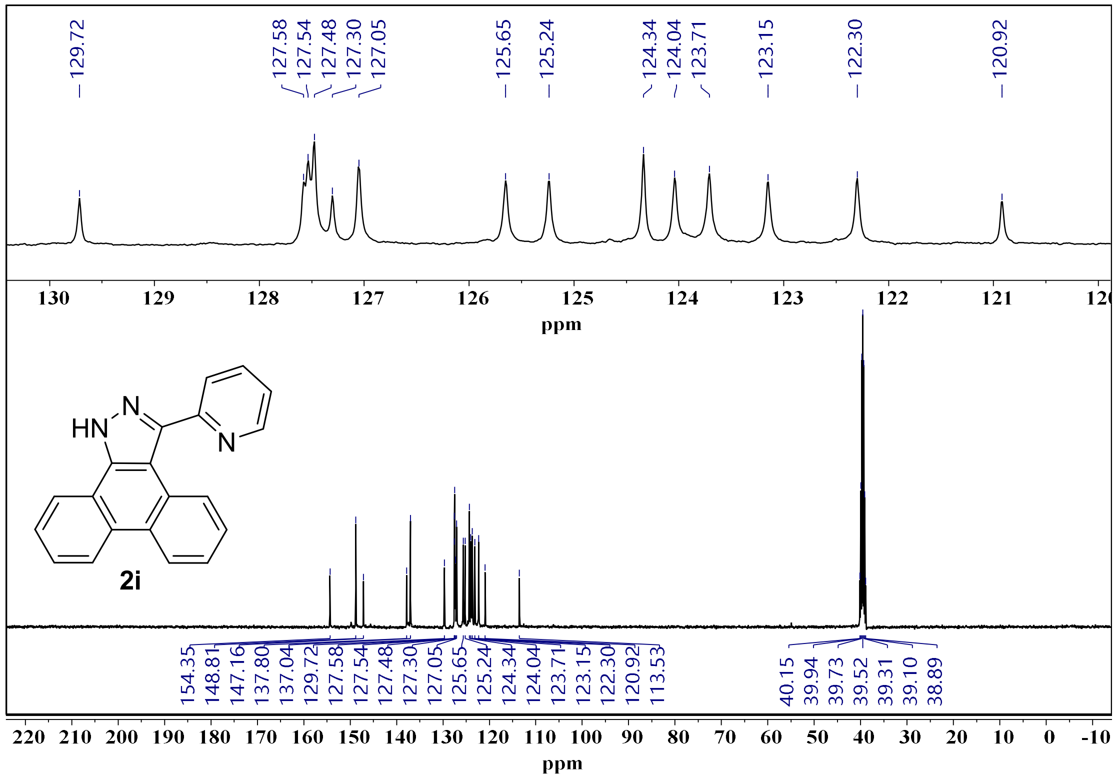

^{1}H NMR (400 MHz, DMSO-d_6) chart of **2j**

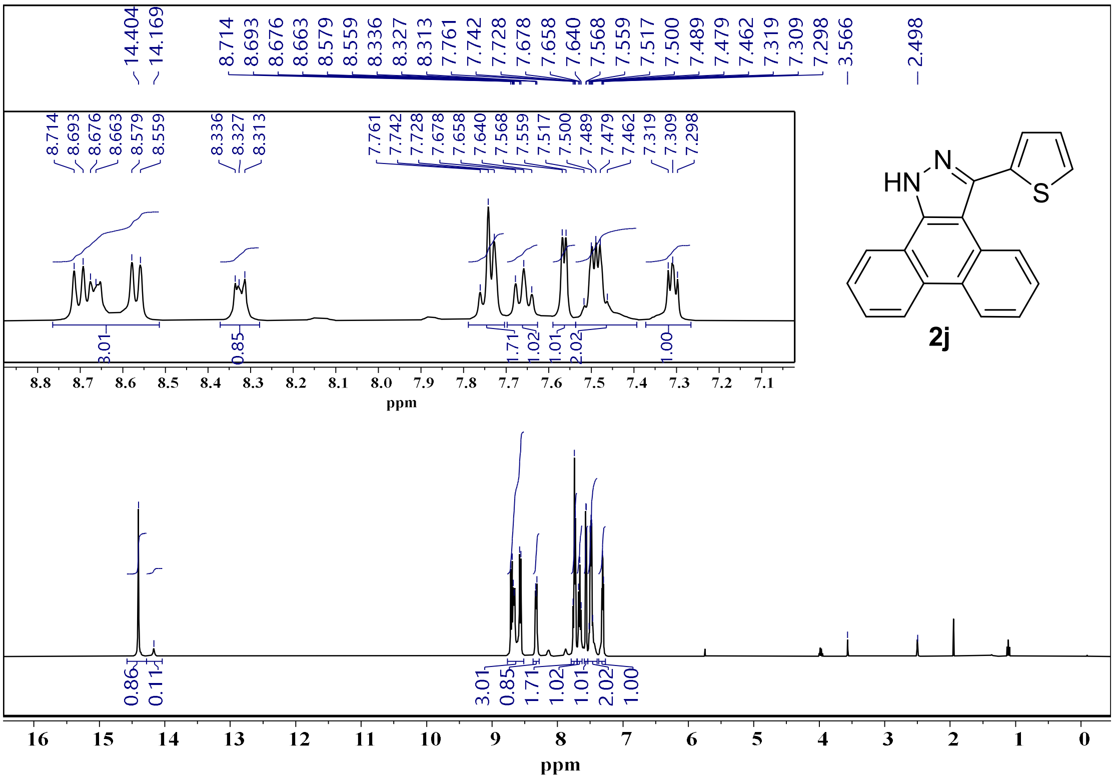

$^{13}C\{^{1}H\}$ NMR (101 MHz, DMSO-d_6) chart of **2j**

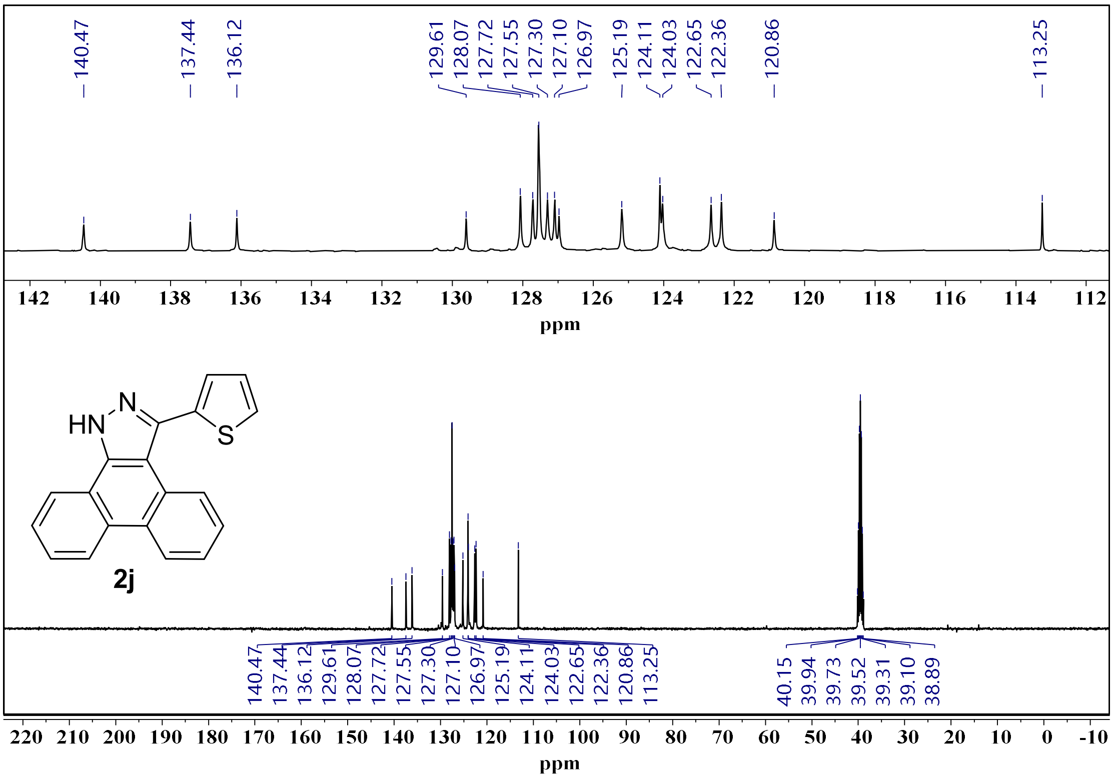

^{1}H NMR (400 MHz, $CDCl_3$) chart of **2k**

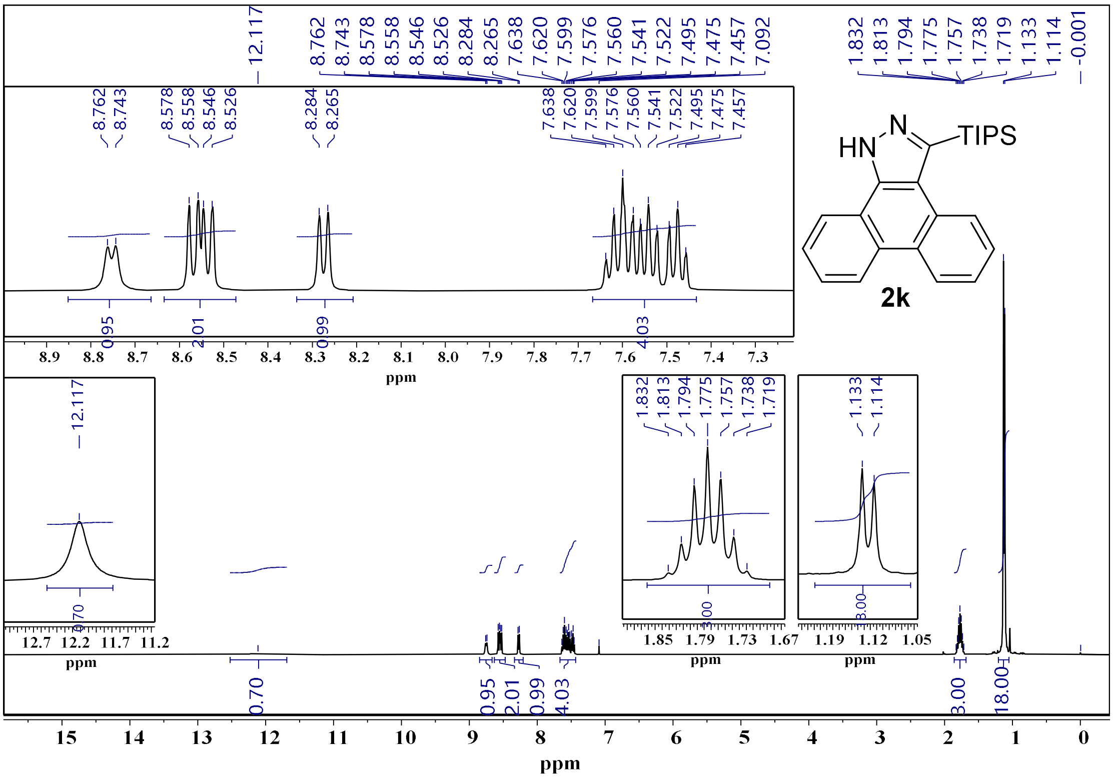

^{13}C{^{1}H} NMR (101 MHz, $CDCl_3$) chart of **2k**

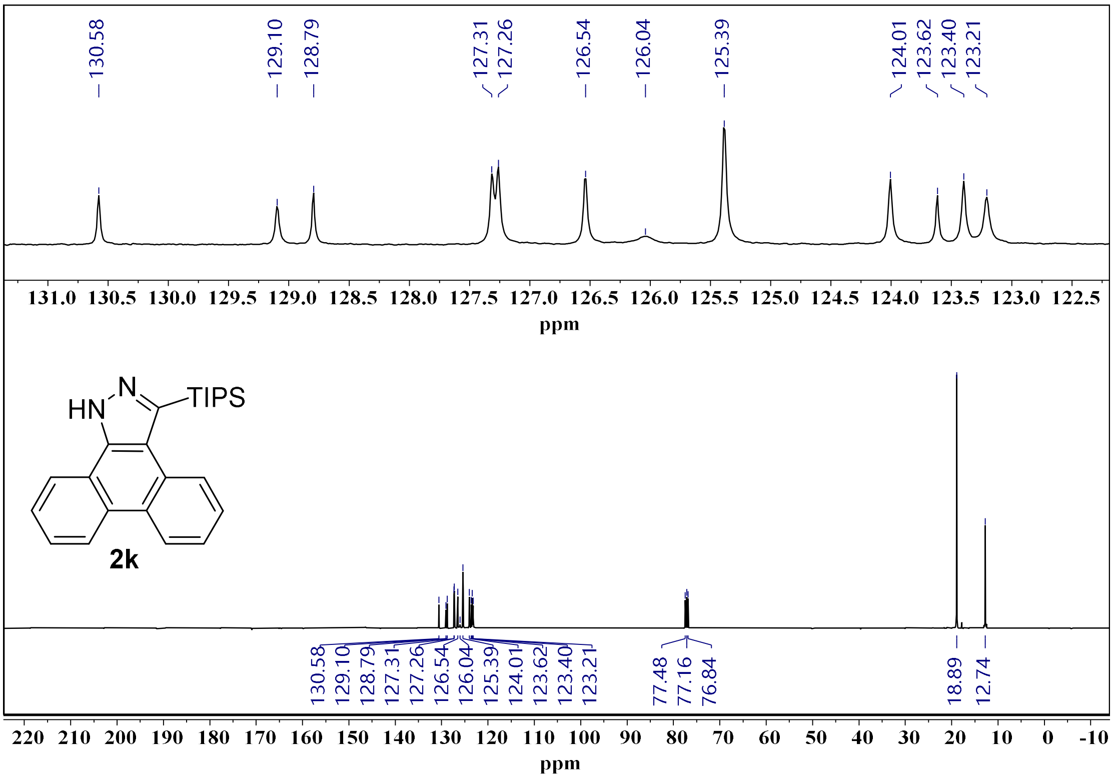

^{1}H NMR (400 MHz, DMSO-d_6) chart of **2l**

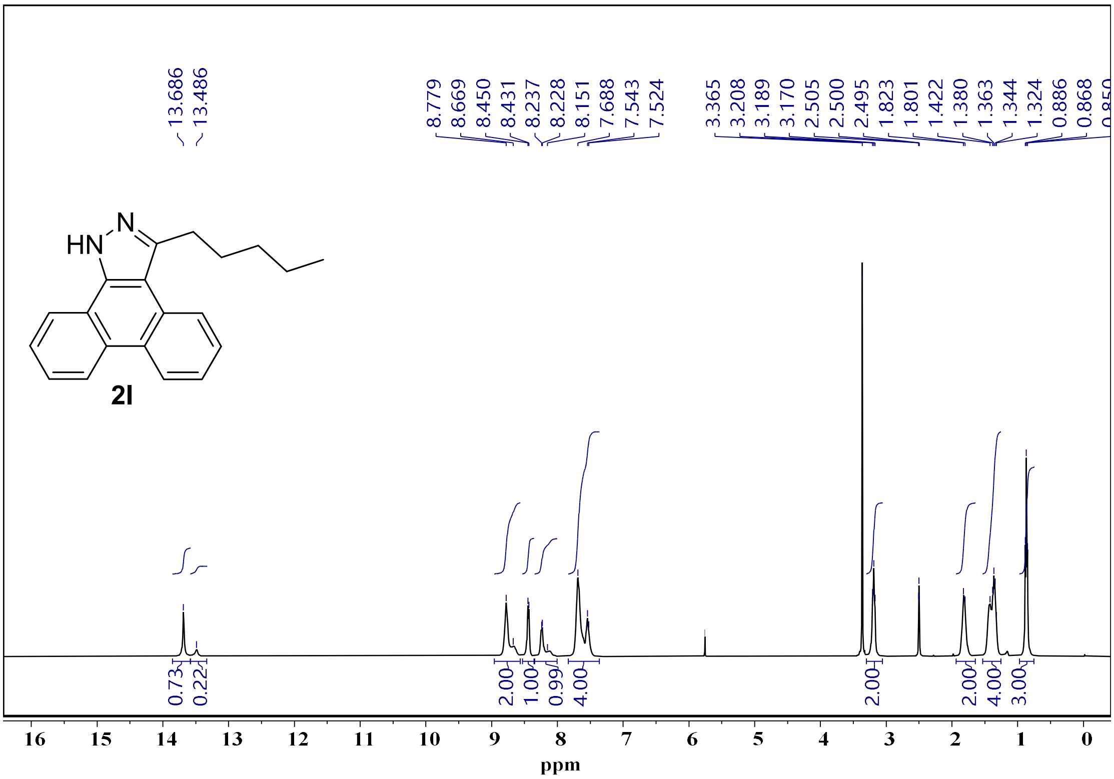

$^{13}C\{^{1}H\}$ NMR (101 MHz, DMSO-d_6) chart of **2l**

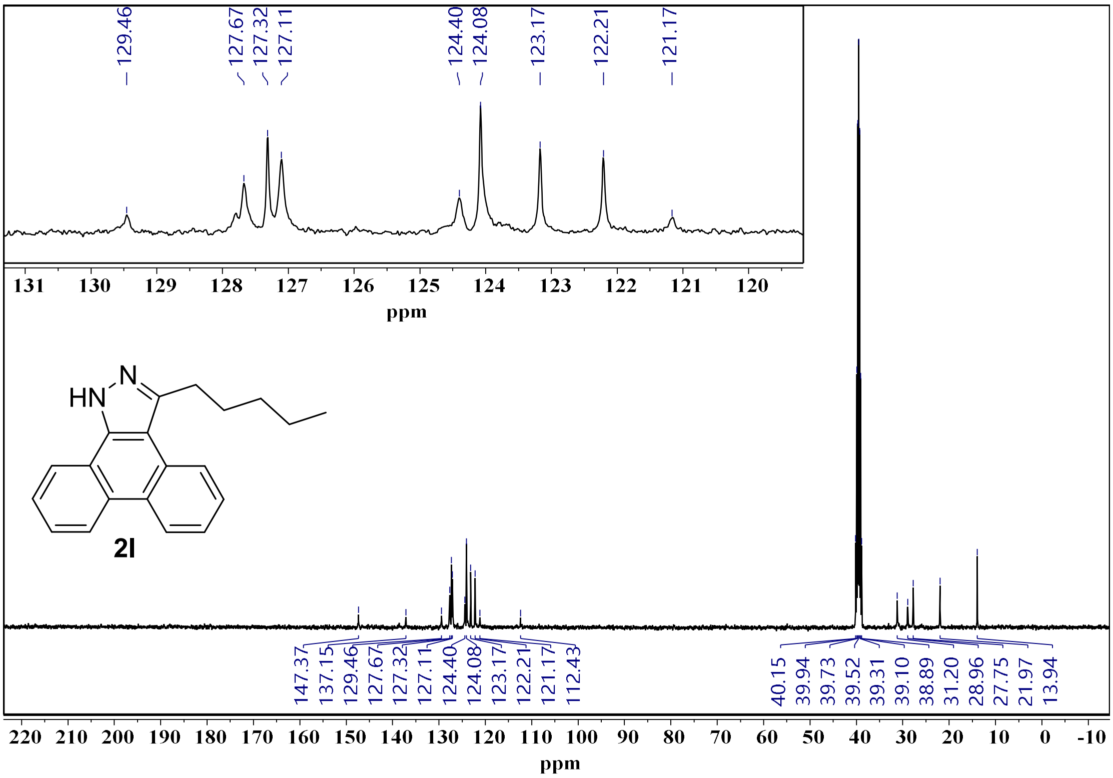

^{1}H NMR (400 MHz, $CDCl_3$) chart of **2m**

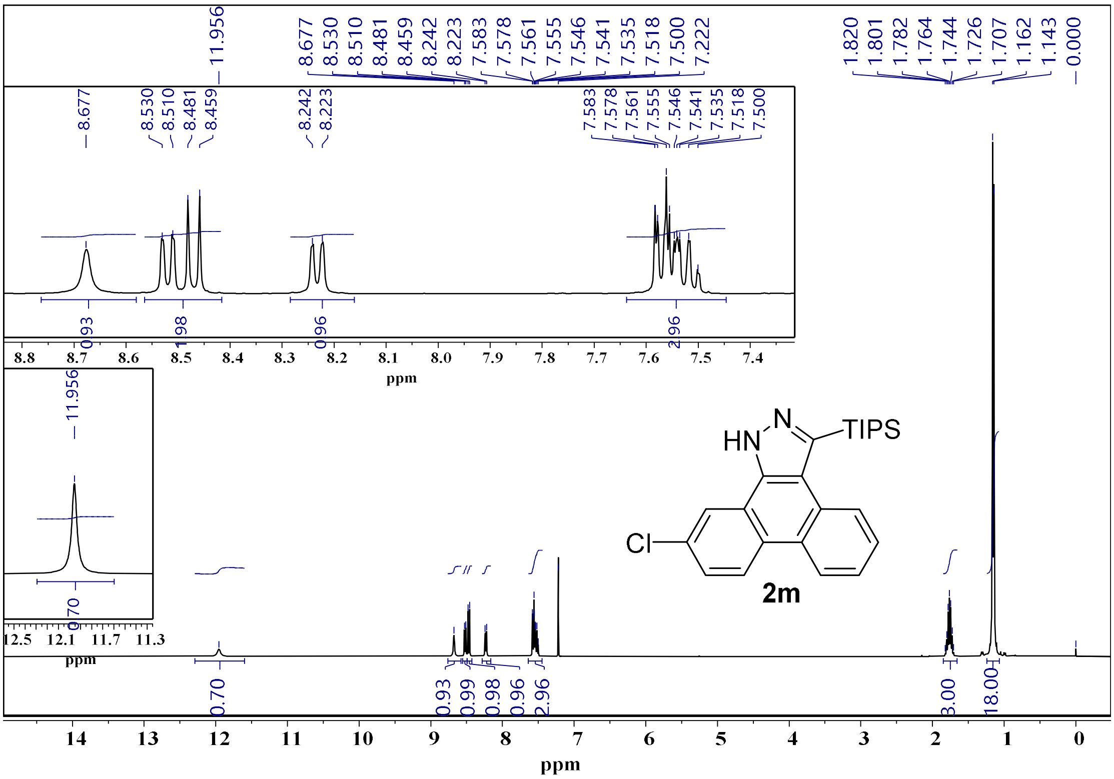

$^{13}C\{^{1}H\}$ NMR (101 MHz, $CDCl_3$) chart of **2m**

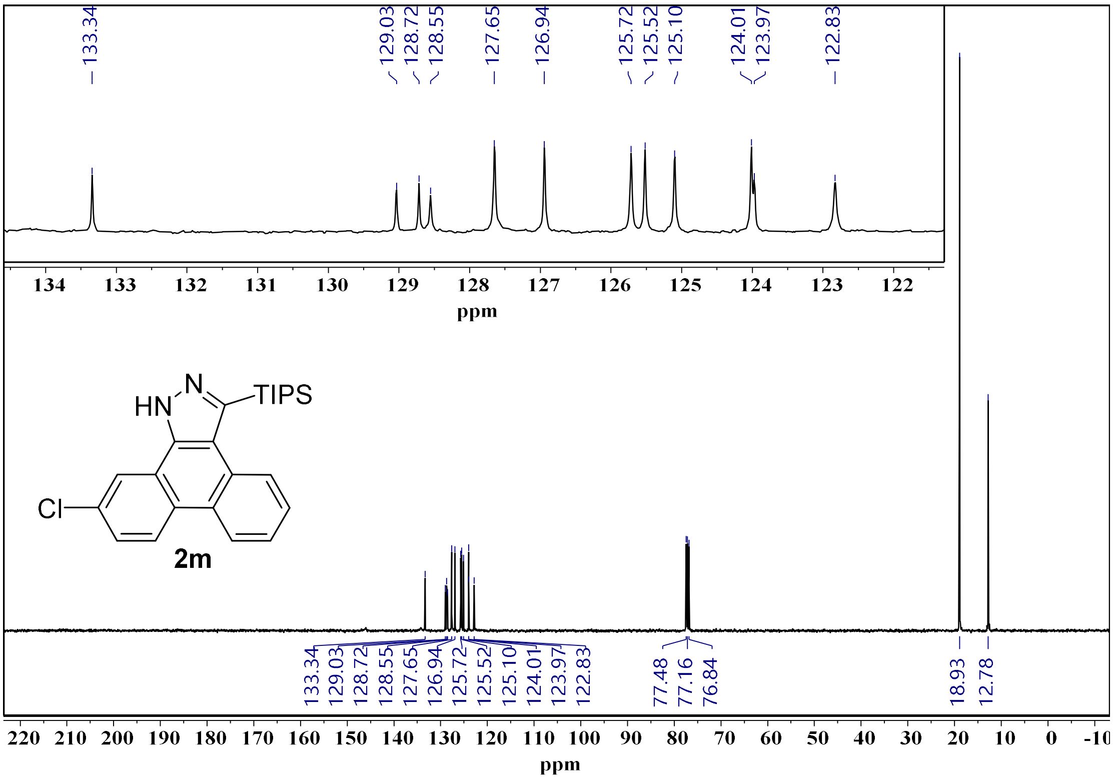

^{1}H NMR (400 MHz, DMSO-d_6) chart of **2n**

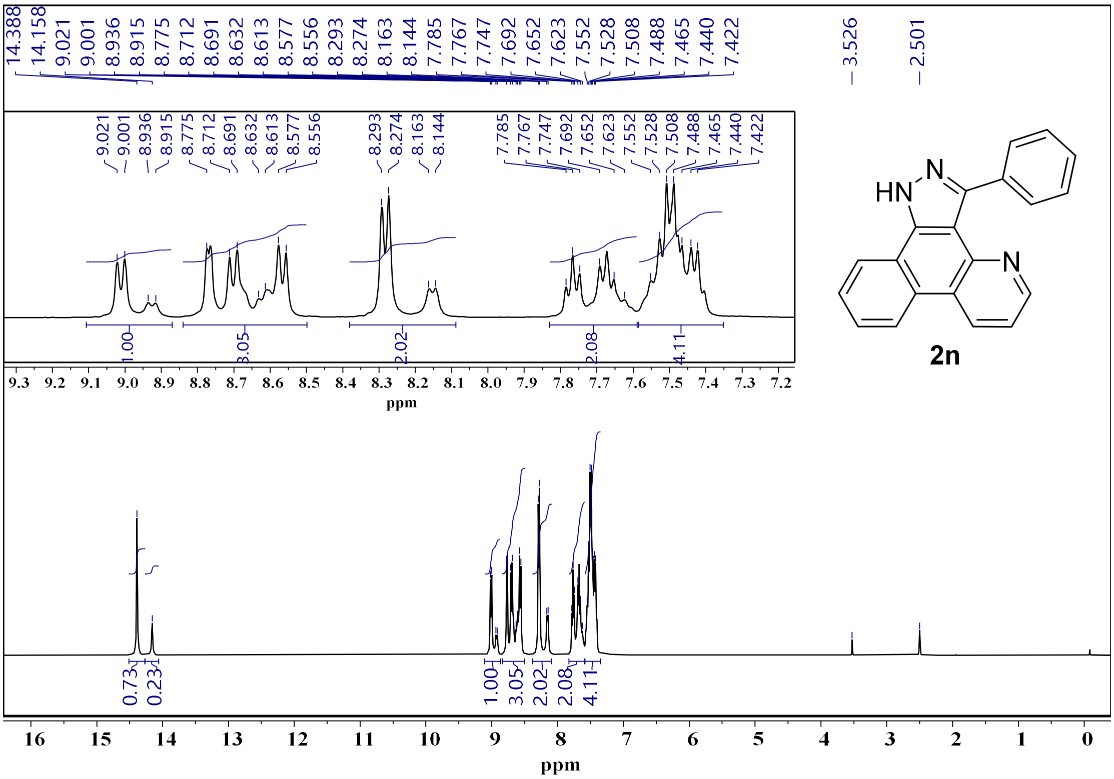

^{13}C{^{1}H} NMR (101 MHz, DMSO-d_6) chart of **2n**

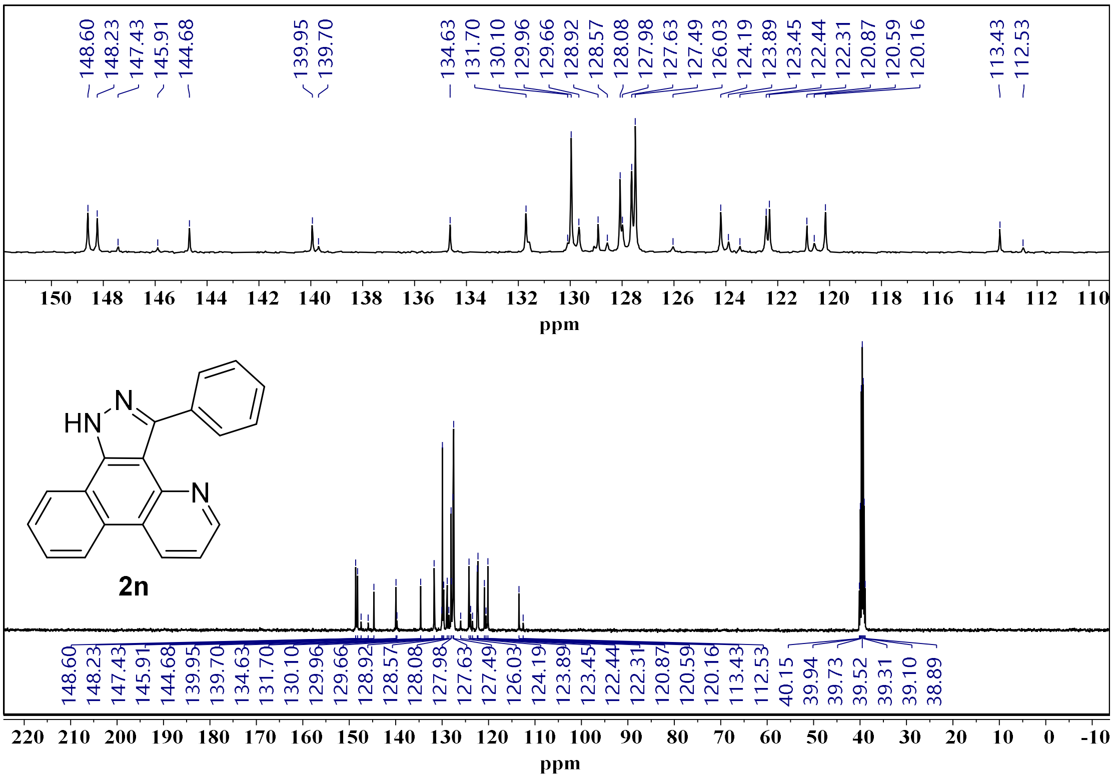

^{1}H NMR (400 MHz, DMSO-d_6) chart of **2o**

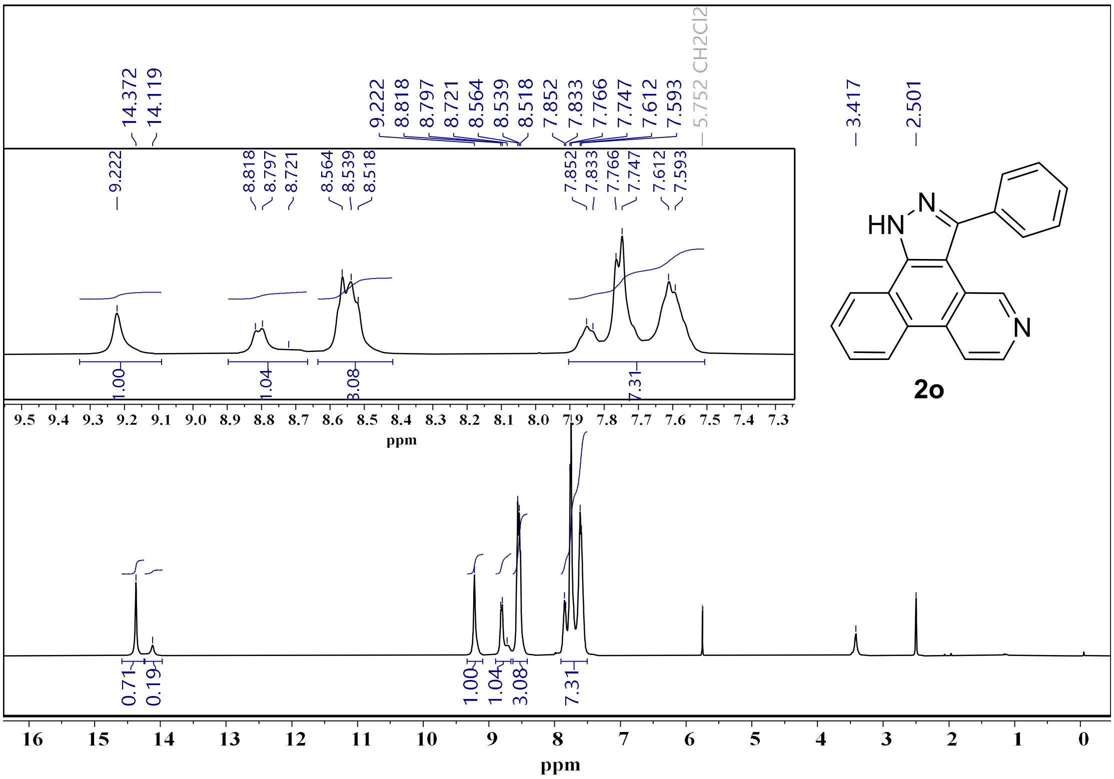

^{13}C{^{1}H} NMR (101 MHz, DMSO-d_6) chart of **2o**

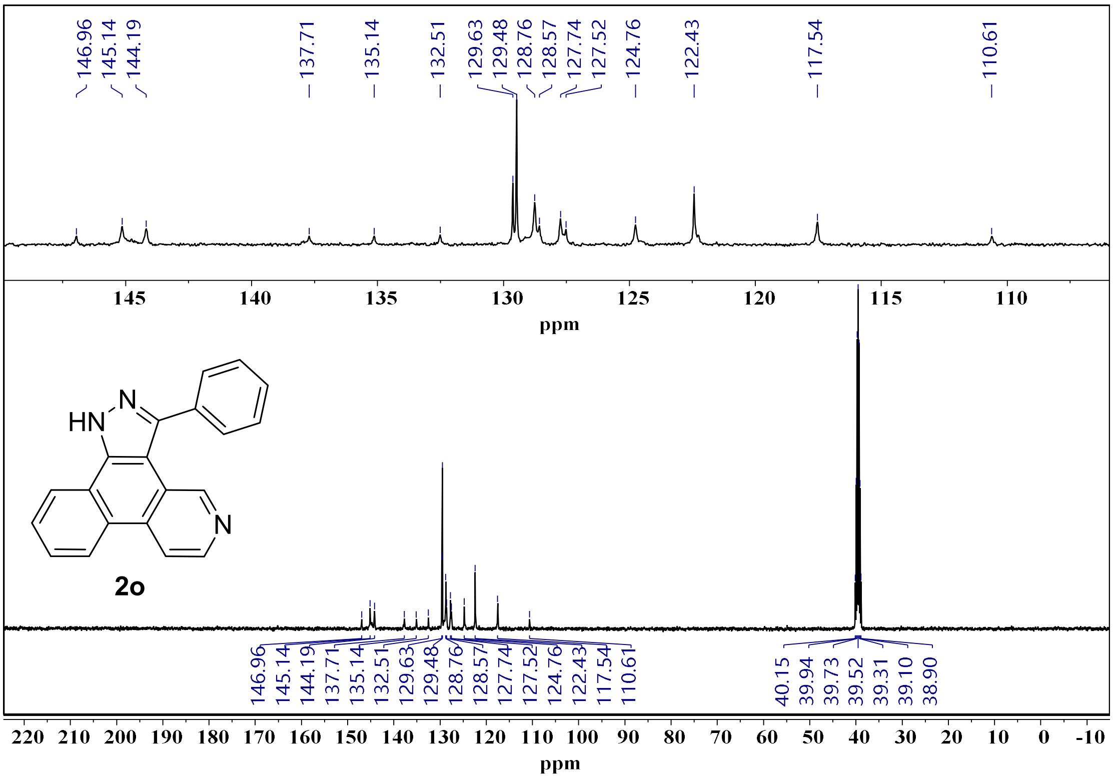

^{1}H NMR (400 MHz, DMSO-d_6) chart of **2p**

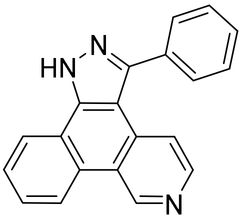

2p

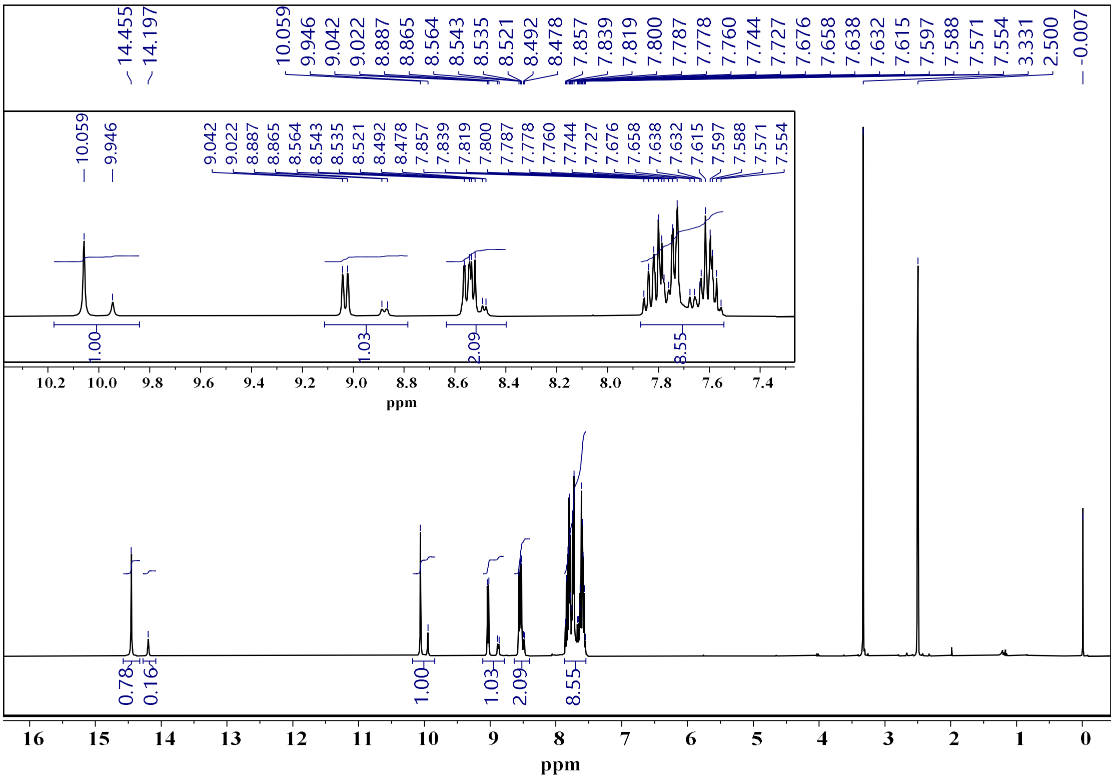

Supplement: Supplementary file 1 [file molecules-28-08061-s001.zip › molecules-2679963-supplementary.pdf]
